# GAMOLA2, a Comprehensive Software Package for the Annotation and Curation of Draft and Complete Microbial Genomes

**DOI:** 10.3389/fmicb.2017.00346

**Published:** 2017-03-23

**Authors:** Eric Altermann, Jingli Lu, Alan McCulloch

**Affiliations:** ^1^AgResearch Limited, Grasslands Research CentrePalmerston North, New Zealand; ^2^Riddet Institute, Massey UniversityPalmerston North, New Zealand; ^3^AgResearch Limited, Invermay Agricultural CentreMosgiel, New Zealand

**Keywords:** genome annotation, microbial, sequence analysis, stand-alone software, genome visualization, expert curation, Artemis genome viewer

## Abstract

Expert curated annotation remains one of the critical steps in achieving a reliable biological relevant annotation. Here we announce the release of GAMOLA2, a user friendly and comprehensive software package to process, annotate and curate draft and complete bacterial, archaeal, and viral genomes. GAMOLA2 represents a wrapping tool to combine gene model determination, functional Blast, COG, Pfam, and TIGRfam analyses with structural predictions including detection of tRNAs, rRNA genes, non-coding RNAs, signal protein cleavage sites, transmembrane helices, CRISPR repeats and vector sequence contaminations. GAMOLA2 has already been validated in a wide range of bacterial and archaeal genomes, and its modular concept allows easy addition of further functionality in future releases. A modified and adapted version of the Artemis Genome Viewer (Sanger Institute) has been developed to leverage the additional features and underlying information provided by the GAMOLA2 analysis, and is part of the software distribution. In addition to genome annotations, GAMOLA2 features, among others, supplemental modules that assist in the creation of custom Blast databases, annotation transfers between genome versions, and the preparation of Genbank files for submission via the NCBI Sequin tool. GAMOLA2 is intended to be run under a Linux environment, whereas the subsequent visualization and manual curation in Artemis is mobile and platform independent. The development of GAMOLA2 is ongoing and community driven. New functionality can easily be added upon user requests, ensuring that GAMOLA2 provides information relevant to microbiologists. The software is available free of charge for academic use.

## Introduction

The advent and continued rise of Next Generation DNA sequencing has enabled microbiologists to investigate more and more microbes on a genome level. Recent deep sequencing projects have generated metagenomic datasets that reach sufficient coverage to assemble genes, operons and—in some cases—larger contigs and draft genomes (Ross et al., 2016; Sangwan et al., 2016), granting insights into the non-culturable biosphere. One of the primary objectives in the subsequent data analyses is the identification of genes and, where possible, the prediction of their respective biological functions.

In 2003 the prokaryotic genome annotation pipeline GAMOLA (Altermann and Klaenhammer, 2003) was developed with the aim of providing microbiologists with a user friendly system for effective and reliable (draft) genome annotation. The fully localized annotation pipeline enabled the analysis of confidential or otherwise sensitive sequences without the need for remote data access or otherwise transmitting sequences. Since then, a number of other genome annotation systems have been established—ranging widely in scope, functionality and data analysis philosophies. Perhaps the most well-known and elaborate remote data processing system is the Integrated Microbial Genomes (IMG) system developed and hosted by the Joint Genome Institute and the Lawrence Berkeley National Laboratory (Markowitz et al., 2009, 2014, 2015). Other systems provide more specialized services, such as gene syntax analysis (Cruveiller et al., 2005), identifying possible problems in annotated genomes through genomics (Poptsova and Gogarten, 2010), comparative analyses of microbial genomes (Altermann, 2012; Overmars et al., 2013) or suggesting rules and standards for (meta-)genome annotations (Angiuoli et al., 2008). A smaller number of pipelines (e.g., AGeS, MyPro, MEGAnnotator, IGS annotation engine, GIT genomics pipeline, RASTtk, Ergatis, and Prokka) is dedicated to providing the means to analyse microbial genomes using local resources in the same way the original GAMOLA software did (Kislyuk et al., 2010; Galens et al., 2011; Kumar et al., 2011; Seemann, 2014; Brettin et al., 2015; Liao et al., 2015; Lugli et al., 2016).

Here we present the second major release of the localized microbial genome annotation pipeline GAMOLA2. The new release represents a complete re-write of the original command line code and introduces a flexible graphical user interface and a modular concept that facilitates the continuous addition of new tools as requested by the user base. The project was initiated in 2007. New functionalities were added and the output format was refined based on continuous user feedback.

While GAMOLA2 requires a Linux based system to generate the comprehensive genome annotation, the use of a customized version of the Artemis Java application (Rutherford et al., 2000) ensures platform independence for subsequent expert curation and analyses. GAMOLA2 has been tested and validated on a wide range of draft and completed bacterial and archaeal genomes (Ventura et al., 2006; Attwood et al., 2008; Azcarate-Peril et al., 2008; Hagen et al., 2010; Leahy et al., 2010, 2013; Lu et al., 2010; Nelson et al., 2010; Altermann and Klaenhammer, 2011; Cookson et al., 2011; Goh et al., 2011; Yeoman et al., 2011; Altermann, 2012; Crespo et al., 2012; Sturino et al., 2013, 2014; Kelly et al., 2014; Lambie et al., 2014, 2015; Cavanagh et al., 2015). In addition to the core genome annotation functionality, several modules have been implemented to aid in managing and publishing microbial genomes.

The GAMOLA2/Artemis software package is available free of charge for academic use.

## Description

### Objective

GAMOLA2 was developed to provide a comprehensive and relevant automated annotation of draft and completed microbial genomes for microbiologists by assembling a wide array of different analyses. The annotation that is provided should fulfill criteria that represent a consensus of many microbiological teams and users. The most important criteria were that the annotation should provide biological background information wherever possible, be easily accessible and visually congruent, and access to the annotation data must be fast, mobile and as platform independent as possible. Further requests included the facility to deal with confidential/sensitive sequences, track changing draft sequences and the ability to access the full range of results obtained for each predicted gene.

To realize these standards, GAMOLA2 was developed with the aim to provide a completely localized microbial annotation platform that can be executed on small to medium sized computing resources without the need for underlying dependencies such as database systems or web-interfaces. The primary output of GAMOLA2 is a comprehensively annotated Genbank file, supported by a range of text-based data files. A customized version of the Artemis genome viewer (version 16) (Rutherford et al., 2000) has been developed to take advantage of the additional features GAMOLA2 provides and is part of the software distribution.

GAMOLA2 attempts to anticipate the most common user mistakes observed over time and will internally correct them wherever possible or inform the user before proceeding with the analysis. A log file with all errors encountered during the analysis is maintained and can be accessed for detailed troubleshooting.

### Framework

GAMOLA2 primarily represents a wrapper to bring together a wide range of specialized individual software tools. This core functionality is then enhanced by a number of custom routines (such as the intergenic Blast analysis). The pipeline is written entirely in Perl and Perl::Tk and has been developed with the ActivePerl 5.8.8.822 distribution and on CentOS release 6.7, using Xming on a Windows host. Has been further tested a Fedora release 21 virtual box on a Windows host and on an Apple PC running XQuartz connected to a CentOS server. The ActivePerl distribution is included as RPM package and tarball and must be installed if not already present. GAMOLA2 is fully multithreaded and can utilize multiple CPUs and cores to reduce runtimes significantly. Other minor dependencies (i.e., presence of “unrar” and the Java runtime environment) are described in more detail in the GAMOLA2 manual.

Installing software can sometimes be a difficult process, requiring the acquisition of numerous dependencies. The GAMOLA2 distribution comes with all software tools and specialized databases provided (with the exceptions of TMHMM Krogh et al., 2001, and SignalP Dyrlov Bendtsen et al., 2004; Petersen et al., 2011, that must be obtained separately) and, once ActivePerl is available on the system, will perform an automatic installation and compilation of all required tools, folder structures, databases and default thresholds when run for the first time. On subsequent runs, GAMOLA2 will test if all resources required are present before each annotation start and, when necessary, recompile missing tools automatically. Only large databases—such as the non-redundant NCBI databases must be downloaded separately, due to their increasing size. A complete list of software tools used and their respective links can be found in the software manual.

A typical annotation run creates up to 3.5 Gb of data for a 4.5 Mbp genome with ~7,000 predicted genes. Using 30 cores and the NCBI non-redundant Blast database, the annotation run took ~4 days to complete. Selecting a more targeted Blast database (e.g., SwissProt or NCBI RefSeqs) will reduce runtimes considerably. The actual amount of data generated varies based on the size of the predicted gene model and analyses selected. The entire annotation can be compressed into a single archive to simplify its distribution across multiple systems and users.

### Input

GAMOLA2 recognizes FASTA and Genbank files as input formats. Both FASTA and Genbank files may contain multiple entries (msFASTA and msGenbank) and can be combined within an annotation run.

The annotation pipeline is explicitly designed to process draft genomes: individual contigs, input files and combinations thereof can either be treated as separate entities or concatenated using a non-bleeding spacer sequence that prevents genes from bleeding across contig boundaries.

Genbank files that harbor a gene model comprising of “gene” and “CDS” features may either be updated or re-created. It is also possible to combine selected input files into groups that are subsequently concatenated.

In addition, external gene models may be provided to force a specific genome annotation.

### Workflow

**Figure 1:**
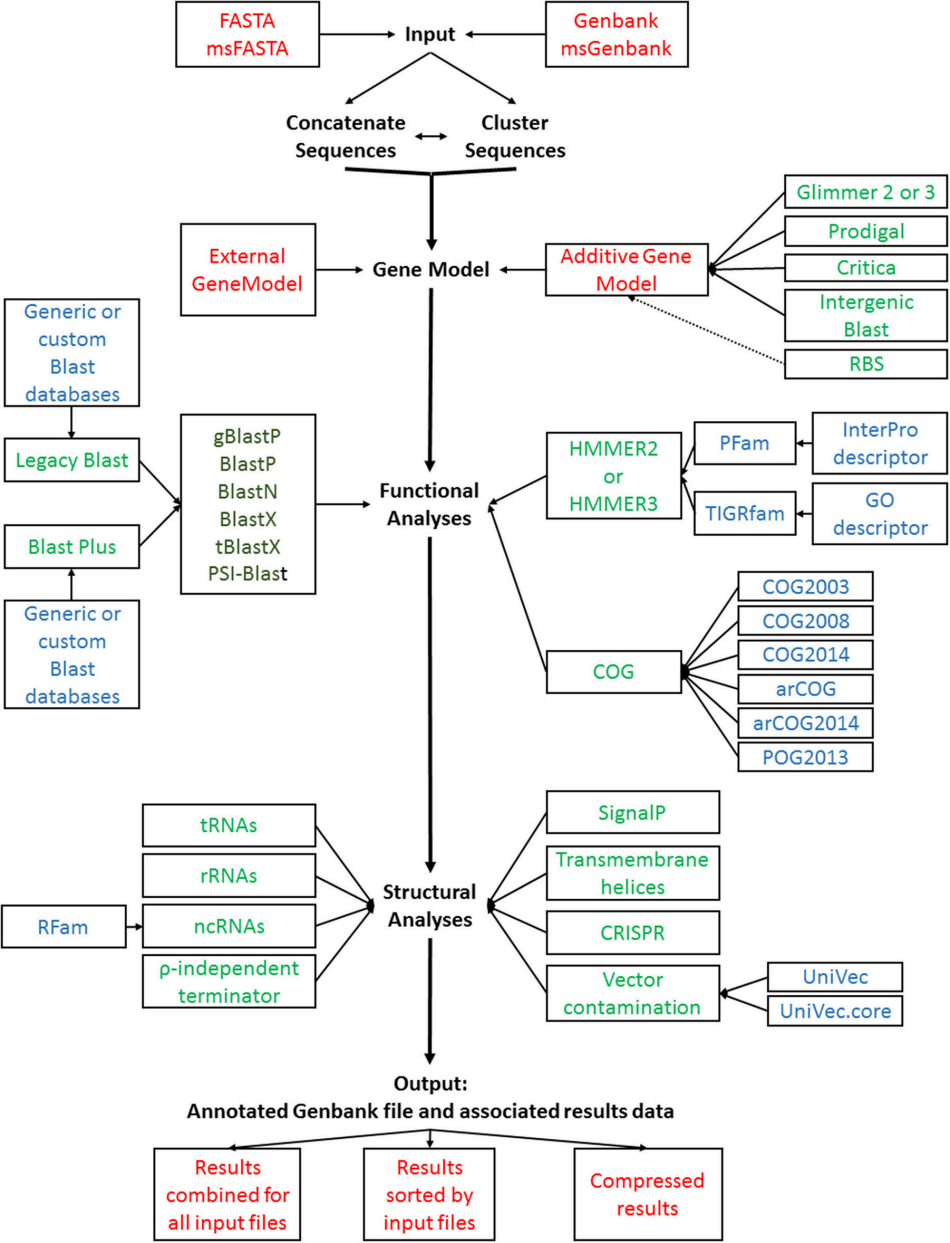
**GAMOLA2 annotation workflow**. A schematic representation of the GAMOLA2 core annotation workflow. Input FASTA and Genbank sequences can be concatenated and/or clustered before submitted to gene model prediction, functional and structural analyses. The final output comprises an annotated Genbank file and associated data that can be viewed in Artemis or other suitable software. For convenience, the results may be compressed into a single archive file. Individual input or output files are shown in red (each analysis generates text output files well which are stored in their respective directories, not shown); programs are shown in green, available Blast flavors are shown in dark green; databases used are indicated in blue.

The increased number of options and parameters offered in the annotation pipeline made the use of a simple command line interface too cumbersome for efficient use. GAMOLA2 therefore now features a graphical user interface (GUI) than leads logically from an initial system parameter setup, to selecting functional and structural analyses, to database selection and input file organization. Once the runtime parameters have been set, the entire configuration can be saved and may be re-used at the next annotation run. Alternatively, default settings can be loaded to restore the original configuration. A general overview of the core options and workflow for GAMOLA is shown in Figure 1.

The following provides a general overview of the GAMOLA2 pipeline and its main options. For more detailed information on individual options, refer to the software manual [provided in the distribution and as a Supplemental File (Supplemental Presentation 1)].

#### Systems setup

Upon invoking GAMOLA2, the system setup offers a number of options to adapt the behavior of the pipeline to the respective system it runs on and the specific annotation outputs. When continuing from a previous or an interrupted run, existing results may be re-used to reduce run-time. Where results are being re-used, existing data files are tested individually for integrity and, when found to be corrupted, are removed and run again. The final annotation may be consolidated by creating a gene model with sequential gapless gene numbers. For convenient transfer the entire output can be archived into a single file (Supplemental Figure 1). Other system options allow users to filter Blast results, providing the option to ignore Blast hits that match specific key words for the annotation (Supplemental Figure 2). Where Genbank files are used as input files, GAMOLA2 can either create a new Genbank file, erasing existing data, or instead update selected analyses (Supplemental Figure 3). Updating existing Genbank files allows all genes to be re-examined against updated or other custom databases, using the embedded gene model. Only selected analyses will be updated, while retaining all other existing features. Existing “gene” and “CDS” annotations can be maintained if manual curation has already been carried out, preventing the loss of expert annotations throughout different rounds of analyses. Default and custom Genbank headers for input files can be built using a point-and-click system and respective field values be pre-configured and saved (Supplemental Figures 4A–C).

#### Main options

The core functionality of microbial genome annotation comprises the determination of a gene model and subsequent analyses of the deduced gene against a selection of databases that provide insights into possible biological function (Supplemental Figure 5A).

GAMOLA2 accepts external gene models in general feature format (GFF) and an internal format in cases where a specific gene model is desired. Genbank input files with an embedded feature list may be updated while preserving the existing gene model. In all other cases, a new gene model is created. Presently, GAMOLA2 supports four different gene callers (Glimmer2 (Delcher et al., 1999) or Glimmer3 (Delcher et al., 2007), Prodigal (Hyatt et al., 2010) and Critica (Badger and Olsen, 1999; Supplemental Figure 5B). In addition, an intergenic Blast can be carried out to identify potential frame shifts, premature stop codons or, in case of fragmented draft genomes, incomplete open reading frames (ORFs) located at contig boundaries (Supplemental Figure 5C). The intergenic Blast is highly customisable and allows users to specify the minimum intergenic ORF (igORF) length and how far a potential intergenic region may reach into existing adjacent genes. Potential ORFs can be determined either via an orientation-aware algorithm (a separate igORF search in sense and antisense orientation, respectively) or by flattening the gene model (igORFs are considered for intergenic regions between all genes). Identified candidate ORFs are then subjected to a BlastP analysis against either standard or custom databases and those with hits below a chosen e-value threshold are added to the gene model. When multiple gene calling algorithms are combined in one annotation run, an additive gene model will be formed, featuring the highest number of the largest potential genes. While this approach increases the potential for false positives, we found that it is more beneficial and faster to remove individual genes or features during expert curation in Artemis than manually investigating regions with potential missed genes.

Once the gene model has been created, genes can be analyzed against Blast (e.g., NCBI nr/nt, NCBI RefSeqs, SwissProt, or other custom Blast databases), COG, PFam, and TIGRfam functional databases. For Pfam and TIGRfam analyses, several levels of verbosity can be selected to include detailed domain descriptions as well as additional Interpro (Pfam) and GeneOntology (TIGRfam) information. Often TIGRfams feature distinct gene names and GAMOLA2 offers an option to preferentially create gene annotations from TIGRfam gene designations for the automated annotation.

In some cases, legacy versions of specific tool may be desired and GAMOLA2 supports legacy Blast, and hmmer2 alongside the recent Blast plus and hmmer3 distributions.

#### Supplemental structural analyses

Aside from the core functional databases, structural features—both within a gene and located in intergenic regions-can provide valuable information on gene context and protein function. Where applicable, analyses can be adapted to specific genome requirements by changing the default parameters.

Transfer RNAs (tRNA) are determined using tRNAscan-SE (Lowe and Eddy, 1997), while non-coding RNAs (ncRNA) are detected using Infernal (Griffiths-Jones et al., 2003). Ribosomal RNAs (rRNA) can either be predicted via Infernal or deduced by a custom build database (provided with the distribution). In the latter case, Blast alignments are analyzed and full length rRNA genes extrapolated based on respective alignment positions (Supplemental Figure 6A).

The location of proteins within a cell can be of importance and may give first clues in cases of conserved hypothetical genes. The prediction of transmembrane helices (Krogh et al., 2001) and signal peptide cleavage sides (Petersen et al., 2011) has been incorporated into GAMOLA2. The transmembrane helix analysis may further be configured to display the position and length of individual helices within a gene (Supplemental Figure 6B).

Other DNA structures such as rho-independent terminators (Kingsford et al., 2007) or CRISPR repeats (Bland et al., 2007) may provide additional information on potential operon structures and genome plasticity, respectively (Supplemental Figure 6C).

Vector contamination may occur, particularly in draft genomes and metagenomes when filter steps had to be avoided. GAMOLA2 can detect such contaminations by screening sequences against the UniVec or UniVec_core databases (http://www.ncbi.nlm.nih.gov/tools/vecscreen/univec/, Supplemental Figure 6D).

A current known limitation of GAMOLA2 is the absence of microbial promoter prediction.

#### Databases

Most functional and some structural analyses require dedicated databases. For Blast, both standard public databases (such as the non-redundant Blast database maintained by the National Center for Biotechnology Information (NCBI)) as well as custom Blast databases (see below) can be used. Depending on the selected Blast flavor, GAMOLA2 will test if the correct type of Blast database has been chosen and prompt the user in cases of incompatible selections.

Clusters of Orthologous Groups of proteins (COGs) are widely used to provide a high level classification of genes or to summarize the genome. GAMOLA2 supports six different COG databases that are provided with the distribution: COG 2003 (Tatusov et al., 2003), 2008 and 2014 (Galperin et al., 2015), archaeal COGs 2007 (Makarova et al., 2007) and 2014 (Makarova et al., 2015), and the 2013 phage COGs (Kristensen et al., 2013). Where possible, individual COG codes are translated into human readable descriptors during the annotation process and are employed both in the annotated Genbank file(s) as well as in individual COG result files.

By default, the standard Pfam and TIGRfam databases are used for analysis. In some cases, multiple databases may be chosen (e.g., Pfam-A and Pfam-B) for the annotation. If multiple PFam or TIGRfam databases were selected, GAMOLA2 investigates the first selected database (e.g., Pfam-A) and, if at least one hit below the selected threshold was found, moves on to the next gene without analysing subsequent selected databases (e.g., ignores PFam-B). Additional databases are analyzed in the order selected (e.g., Pfam-B), only in cases where no significant hits in the previous database (e.g., Pfam-A) were detected (Supplemental Figure 7).

#### Configuring input sequences and starting the annotation

The annotation of draft and complete genomes and other genetic elements often requires a flexible approach on how input files and embedded entries are processed. Draft genomes may consist of many individual contigs, sometimes across multiple data files, whereas multiple completed genomes are to be analyzed as separate entities within a single annotation run. GAMOLA2 provides a high level of flexibility in the way input files can be combined or disassembled (Supplemental Figure 8).

Draft phase genomes may consist of hundreds of individual contigs and assembled metagenomes often comprise thousands of small sequence fragments. While annotating each contig or fragment individually is possible with GAMOLA2, a more common approach is to concatenate entries in the order given by the input files using a defined spacer sequence that is easily identifiable and prevents ORF-bleeding across contig boundaries by introducing stop codons across all six reading frames (5′-NNNNNNNNNNTTAGTTAGTTAGNNNNNNNNNN-3′). This concatenation can be carried out for both FASTA and Genbank input files, whereby existing gene models for multiple Genbank files are discarded and a new gene model built. Similarly, the presence of “N”s in the nucleotide sequence may represent known gaps and GAMOLA2 can be set to replace those “N”s with the non-bleeding spacer sequence, albeit without breaking the contig. This approach ensures that predicted genes are not allowed to span these undefined regions, increasing the reliability of the gene model.

Finally, multiple input files may be combined into annotation groups that are concatenated into a single entity. This option allows the easy combination of fragmented draft genomes distributed across multiple input files or the merger of multiple replicons of a microbe.

#### Automated annotation

Once all selected analyses have been carried out, GAMOLA attempts to provide an automated annotation for each predicted gene. Automated annotations are generated based on Blast and TIGRfam results. If neither Blast nor TIGRfam is selected in an annotation run, each gene will be annotated as “unknown.” If Blast is selected, gene annotations will be based on the best Blast hit that features an e-value below the user defined threshold. If only Blast hits above the threshold were detected, the gene will be annotated as “conserved hypothetical.” If no Blast hits were found, gene annotation will be set to “unknown.” TIGRfam hits often have well curated gene names and descriptors. If selected, the best TIGRfam hit below the selected e-value threshold will be chosen to override the Blast-based automated annotation for both “gene” and “CDS” features. When selected, E.C. numbers will be added to the “CDS” feature.

#### GAMOLA2 output files

**Figure 2:**
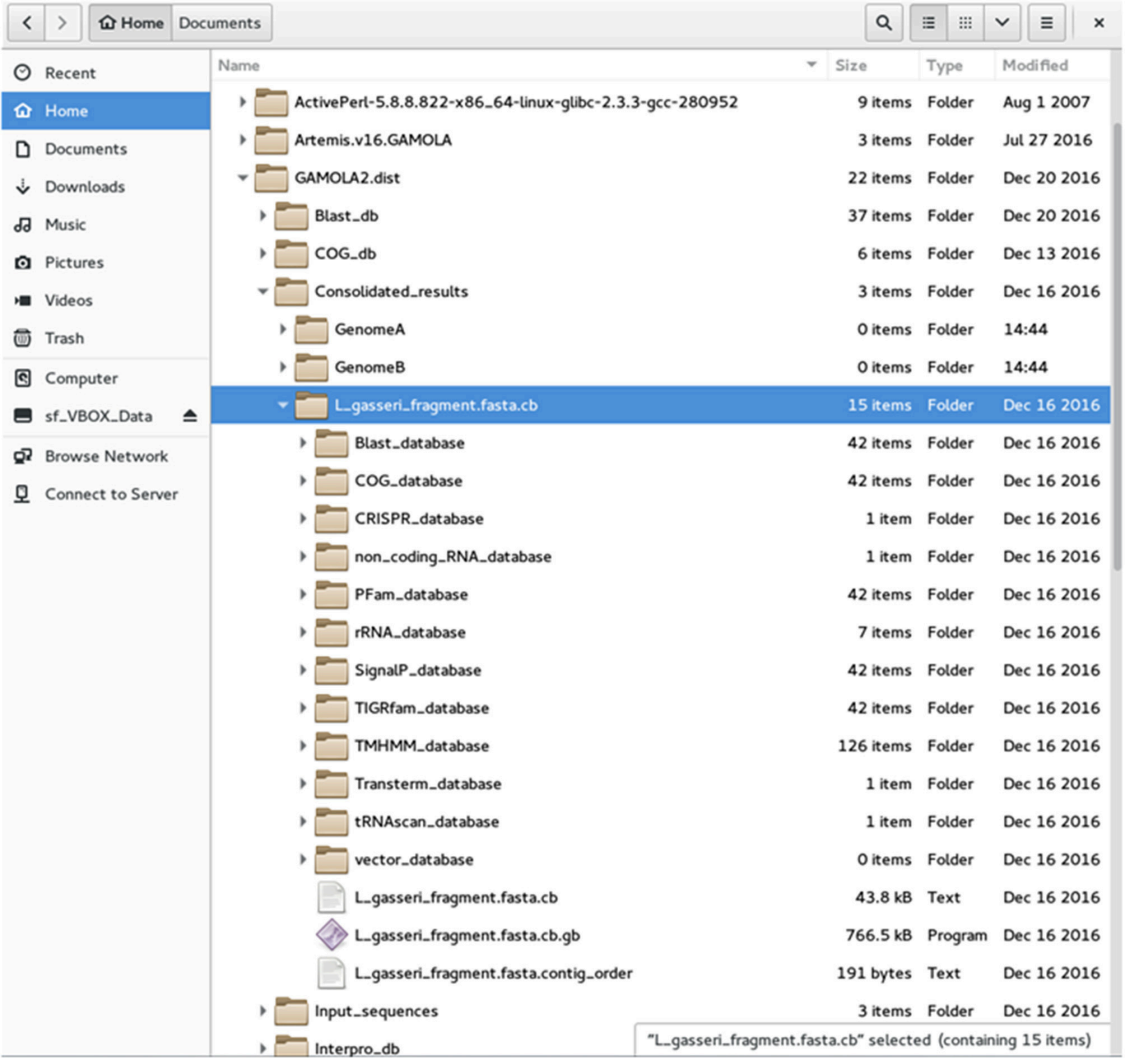
**File structure of the GAMOLA2 output**. Screenshot of the GAMOLA2 file and directory arrangement. Upon completion of an annotation run, GAMOLA2 can sort results of individual entries into separate directories that comprise the main annotated Genbank file, the underlying FASTA sequence file and, where appropriate, the contig order of the concatenated sequence. Further, the full dataset for the entire genome is available in their respective folders and can be easily retrieved for a more detailed analysis and background information.

Once the GAMOLA2 annotation run has finished, several outputs will be available:

- Results for all input files are saved in the “Results” directory and are accessible in individual, analysis-specific directories. These are considered the original data, based on the gene model created.
- GAMOLA2 offers the option to sort individual input files and save respective input-specific results into separate folders (Figure 2). A separate directory is created for each annotation entity which harbors all information created for that annotation. Individual data for each gene are saved in respective analysis-type folders (e.g., Blast_database, COG_database, etc). A FASTA file of the (concatenated) nucleotide sequence and a text file with the contig order (providing contig names and respective start and stop positions) are accompanying files to the annotated Genbank file. The Genbank file harbors all features selected in the GAMOLA2 annotation run. Genes are represented as both a gene and CDS feature for annotation purposes. Further, they are given a unique and sequential gene number that is used to retrieve underlying raw data in Artemis (see below).
- When selected, GAMOLA2 compresses all results into two archive files: – “object_results” contains the raw unsorted data that can be used to re-populate result folders in case analyses need to be re-run, which will reduce runtime. – “consolidated_results” holds the separated and sorted result files for each entity used in the annotation run. This is the archive that will be used for further analyses and curation in Artemis.
- An error log file is created for each annotation run and saved in the home directory. In this file are listed all errors that were encountered during the annotation process. It provides many pointers to problems within the gene model and often proves useful in identifying problems in the input sequences.

#### Genome visualization and curation in the modified artemis genome browser

**Figure 3:**
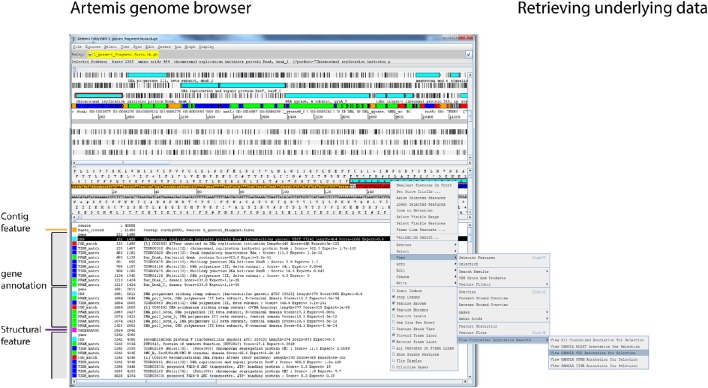
**Genome visualization in Artemis**. Screenshot of the modified Artemis genome browser displaying a GAMOLA2 annotated sequence. The Artemis genome browser is a Java based application that is platform independent and can, once the Genbank file is loaded, traverse along the genome and display information for individual genes in real-time. Annotations for individual genes are presented in individual feature blocks that always begin with the “gene” and “CDS” features (gray boxes). Additional features are shown based on their respective genome location. Each feature has a defined color code, creating a consistent user experience. Changing gene annotations is achieved by modifying the “gene” qualifier in the “gene” and “CDS” features, whereby “gene” features display a short gene name and “CDS” features a verbose description (Supplemental Figure 9A). Names of functional domains are often cryptic and do not directly contribute to the deciphering of the biological role of a given gene. Each feature in a GAMOLA2 annotation therefore contains additional information to explain the respective biological role (where known) or provide additional qualitative details (Supplemental Figure 9B). Genes that lack a close characterized homolog or well-known domains often remain annotated as “conserved hypotheticals.” Investigating all functional and structural information above the selected thresholds often reveals common biological themes that lead to a putative annotation. The modified Artemis genome browser can retrieve the underlying full results for Blast, COG, PFam, and TIGRfam for each gene as long as the original file and folder structure is maintained (Supplemental Figure 9C).

Working with microbial genomes should be fast, flexible, and intuitive. Often, genes are investigated in their wider context and distant loci are frequently targeted when carrying out functional analyses. The Artemis Genome Browser (Rutherford et al., 2000) has been under continuous development since 2000 and still represents one of the best and most flexible genome browsers designed to date. Artemis is a Java-based application and therefore platform independent with no further dependencies required, making it the ideal companion for the GAMOLA2 annotation. We have created a modified version of Artemis with added functionality to take advantage of the GAMOLA2 annotation output. In particular, additional feature keys have been incorporated and given defined color values that create a coherent visual layout for each gene (Figure 3, shaded boxes). Each gene consists of a “gene” and a “CDS” feature with the same start-stop positions, enabling researchers to specify both a short gene name and a more biologically-interpretable description of the prediction function (Supplemental Figure 9A, highlighted qualifier). Within the gene boundaries, functional and structural hits are shown in their assigned color codes, displaying relevant information (e.g., biological roles, *e*-values, alignment lengths and scores) of the respective best hits found. The next gene starts again with the “gene” and “CDS” features. Using this system, it is straightforward to perceive common biological themes across all hits for a given gene and verify or correct the automatic annotation. Where further information is required on the role or composition of individual features, relevant information embedded in the Genbank file can be retrieved directly from within Artemis (Supplemental Figure 9B). A second modification in Artemis provides direct access to underlying Blast, COG, PFam, and TIGRfam results. By selecting a “gene” or “CDS” feature, all or individual analysis results can be retrieved, enabling a more comprehensive insight in biological roles and the presence of homologs (Figure 3 and Supplemental Figure 9C). In particular for poorly characterized genes, investigating functional hits above the selected threshold across all databases may reveal common biological “themes” enabling at least a putative annotation. Where concatenated sequences are present, individual contigs are marked by features in alternating colors, emphasizing contig boundaries to prevent erroneous assumptions on gene synteny across contigs.

The GAMOLA2-Artemis software package enables individual researchers to routinely curate 200 to 250 genes per day. An annotation guideline that suggests an optimized annotation workflow has been added to the user manual and can be easily adapted to respective team requirements.

### Supplemental modules

Microbial genome annotation requires a number of flexible tools to implement specialized analyses and data interpretation. GAMOLA2 offers a range of supplemental modules that extend its functionality beyond that of a pure annotation pipeline. These modules have been developed for GAMOLA2 based on user feedback and real-world requirements regularly experienced, and are given here.

#### Creating custom blast databases

Creating custom Blast databases is required where specific data analysis is required, e.g., comparing a query microbial genome against other known genomes of the same strain/species. GAMOLA2 provides such a module, creating nucleotide and amino-acid Blast databases from (ms)Genbank to (ms)FASTA files. These databases can be rapidly built and then used in subsequent annotation runs (Supplemental Figure 10). To ensure that custom Blast databases are of high integrity, GAMOLA2 tests input files for errors and inconsistencies during the parsing process.

#### Rotating genbank files

Assembled sequences often present a random genome location as starting points. By convention, complete genomes often begin at agreed anchor points, such as origins of DNA replication (e.g., the chromosomal replication initiator protein DnaA) and genomes are routinely re-oriented before submission into sequence depositories. GAMOLA2 features the ability to rotate annotated Genbank files to new starting points, shifting all features accordingly while retaining the original gene numbers (Supplemental Figure 11). When preparing such a rotated Genbank file for submission via Sequin (see below), respective locus tags may then be reset to start with “0001.”

#### Preparing genbank files for submission

Submitting extensively annotated Genbank files is often a time intensive process. One of the most commonly used tools for submission to NCBI is Sequin (http://www.ncbi.nlm.nih.gov/Sequin/) which accepts both manual entry of features as well as a batch submission using a tabulated input file. The genome submission preparation module of GAMOLA2 was developed to minimize the time required to submit a genome to NCBI using Sequin (Supplemental Figure 12). The module supports the preparation of both complete and draft phase genomes and can generate AGP scaffold information data for the latter (https://www.ncbi.nlm.nih.gov/assembly/agp/AGP_Specification/). Where a submission is comprised of multiple entities (e.g., a multi-replicon genome architecture), these can be either linked via locus tags or be treated as individual sequences. While a wide range of features can be selected to be incorporated into the submission, a minimum set consisting of “gene,” “CDS,” and “rRNA” features is recommended. CDS features may be further customized to include specific supplemental qualifiers. The output of this module consists of a FASTA file, the Sequin feature table and, where applicable, the AGP information file.

#### Annotation transfer between genomes

Working with early and advanced draft phase genomes poses the problem of ongoing changes in the assemblies and, consequently, in generated gene models. Expert curation will often start with early draft phase genomes and continue until the genome is closed and validated. The problem, however, is that due to changes in the assembly, curated annotations may not be directly transferrable between assembly versions. GAMOLA2 addresses this problem by enabling a transfer of gene annotations between different assembly versions (Supplemental Figure 13). Both “gene” and “CDS” annotation can be transferred. As a first approach, genes with identical sequences will be captured and annotation transferred. Similarly, genes that have been extended or truncated, will be identified in a second pass. Finally, where amino acid sequences have changed between draft versions, a Blast search is carried out to determine the best fit. The sensitivity of the Blast analysis can be adjusted by changing the minimum percent identify threshold required for the alignment. Ambiguous matches (e.g., multiple gene copies as found for integrases) or no matches between genome versions for a given gene will be recorded in a separate log file and can be validated manually.

#### Custom metagenome analysis

One of the advantages of the new modular structure of GAMOLA2 is the ability to rapidly develop and implement new analyses and customized modules. One such example is the examination of metagenome reads against custom Blast databases with the aim of obtaining a comparative high-level overview of the distribution and levels of similarity against specific protein/enzyme families (Supplemental Figure 14). The purpose of the module is not to provide a detailed and comprehensive analysis of a given metagenomic dataset, but to enable an assessment of the frequency and respective levels of similarity of individual metagenomic reads against a thematic (i.e., a custom Blast database comprising entries of a similar function) Blast database. A known limitation of this type of analysis lies within the Blast algorithm and the calculation of the e-value with respect to database size and query length. Custom Blast databases of very different sizes may impact the e-value while read lengths may vary within a dataset and between different sequencing platforms. Care should be taken when comparing results and read lengths should be filtered for length were possible. Further, input data should be adjusted and have undergone a quality control step before being analyzed.

The module was designed to investigate metagenomic reads in FASTA format (based on the 454-FLX sequencing platform) that are blasted (BlastN, BlastX, or tBlastN) against standard or custom Blast databases. Identical reads may be collapsed (only one representative read will be submitted to Blast, reducing the overall number of queries) and their respective frequencies is reported. An upper e-value threshold can be set to define a minimum level of similarity to subject hits. The analysis provides a number of output files, including the original Blast output, two tab-delimited summary files that provide information on hit frequencies for two respective Blast e-values ranges, and a detailed results overview that can be imported into Excel for detailed data mining. The provided data can be used to create comparative graphical representations between one or more metagenomes and respective custom databases (Supplemental Figure 15). A metagenomic analysis using a custom Blast database comprising 69,869 entries, with a query metagenome (source: IMG genome ID: 3300000524 or NCBI BioProject database: PRJNA244109: 609,709 unassembled nucleotide reads, Ciric et al., 2014) took 70 min utilizing 60 cores on a CentOS server. A comparison between results for specific enzyme classes between IMG/M and GAMOLA2 is shown in Supplemental Table 1. For a chosen e-value threshold of 1e-50 GAMOLA2 results were in general agreement with IMG/M data for most enzyme classes. Differences in results (e.g., for arabinosidases) may result from the underlying Blast database makeup.

### Comparison to other annotation systems

A number of other annotation system have been published over the last decade and perhaps most notable for local microbial annotations are Prokka (Seemann, 2014), ConsPred (Weinmaier et al., 2016), and RAST/myRAST (Aziz et al., 2008). A comparison between all four platforms (Table 1) revealed that each system offers different features that are indicative of respective purposes and philosophies.

**Table 1:** **Feature comparison between GAMOLA2 and ***three*** other annotation suites**.

	**GAMOLA2**	**PROKKA**	**ConsPred**	**RAST/myRAST**
**RUNTIME ENVIRONMENT**				
GUI	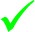	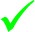 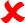	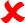	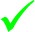
Visualization of annotation	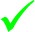 ^(a)^	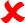	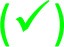 ^(b)^	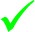 ^(c)^
Local PC	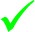	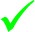	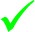	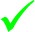
Server-client/terminal	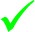	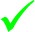	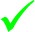	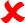
Cloud	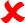	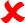	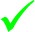	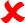
Web-based	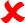	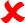	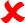	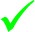
Multi-threaded	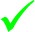	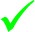	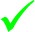	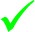
Off-line capability	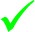	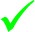	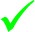	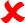
Re-use previous results	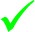	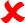	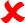	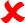
Filter Blast results	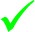 ^(d)^	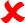	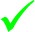 ^(e)^	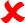
**GENE MODEL PREDICTION**				
Glimmer 2/3	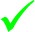	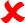	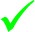	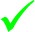
Prodigal	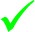	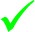	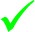 ^(f)^	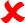
Critica	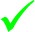	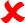	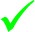	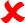
GeneMark	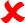	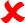	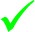	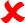
RAST	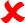	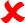	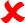	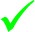
Intergenic Blast	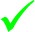	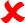	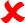	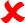
Blast Homology based gene prediction	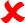	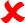	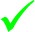	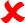
RBS	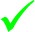	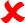	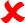	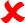
External gene model	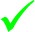 ^(g)^	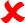	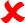	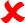
Additive prediction	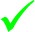	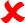	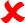	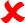
Rule based prediction	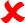	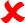	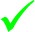	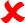
**FUNCTIONAL ANALYSES**				
Blast/Blast-Plus	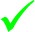	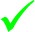	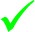	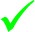
Multiple Blast flavors	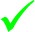	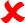 ^(h)^	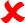 ^(h)^	
Custom Blast databases	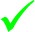	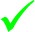	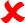 ^(i)^	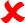
COG	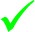		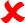	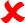
Multiple COG databases	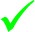 ^(j)^	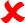	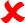	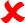
eggnog	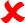 ^(k)^	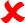	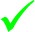	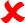
Pfam (HMMER2/3)	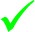	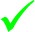	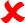	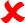
TIGRfam	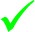	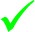	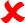	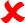
FIGfam	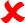	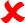	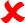	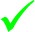
Selection of multiple databases	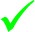 ^(l)^	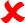	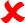	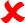
Gene Ontology descriptor	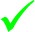	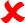	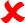	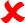
InterPro descriptor	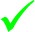	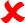	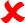	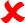
InterProScan	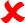	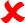	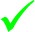	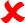
EC number	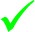	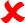	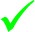	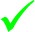
KEGG	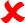	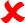	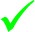	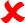
Metabolic reconstruction	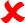	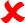	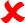	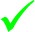
**STRUCTURAL ANALYSES**				
tRNA	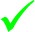	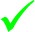	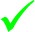	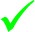
rRNA	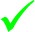	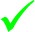	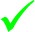	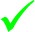
Non-coding RNAs	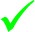	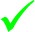	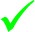	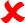
Transmembrane helices		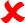	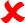	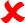
Signal Peptide Cleavage Sites	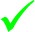	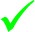	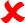	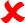
Rho-independent terminators	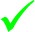	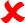	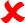	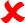
CRISPRs	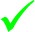	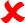	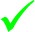	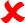
Vector screen	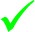	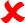	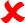	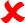
**INPUT FORMATS AND SEQUENCE HANDLING**				
FASTA	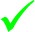	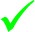	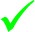	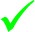
msFASTA	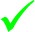	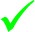	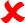	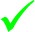
Genbank	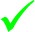	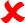	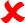	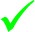
msGenbank	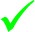	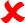	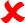	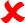
Concatenate sequences	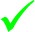	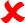	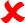	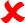
Create concatenated sequence clusters	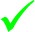	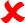	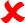	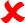
Prevent gene model bleeding across contigs	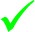	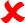	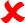	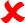
Update Genbank files	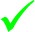	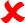	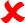	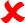
**OUTPUT**				
Genbank	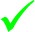	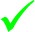	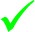 ^(m)^	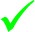
GFF tracks	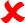	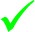	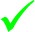	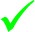
EMBL	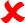	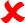	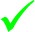	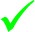
All features displayed	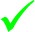	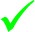	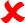	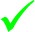
Embedded feature descriptors	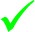	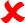	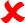	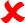
Feature csv/tsv file	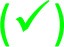 ^(n)^	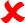	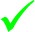	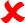
Feature Excel file	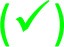 ^(n)^	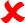	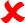	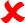
Log and Error files	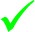 ^(o)^	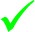	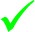	
Statistic file	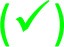 ^(n)^	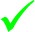	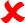	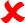
**OTHER FEATURES**				
Create custom Blast databases	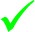	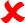	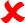	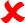
Rotate Genbank files	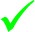	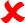	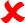	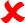
Prepare for Sequin submission	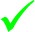	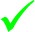	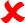	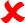
Annotation transfer	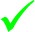	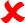	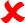	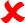
Functional Metagenome analysis	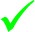	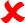	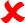	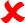

(a) Via enhanced Artemis genome viewer;

(b) static HTML sites to KEGG and KO;

(c) custom browser;

(d) custom filter;

(e) automatic;

(f) default;

(g) internal and GFF format;

(h) blastp only;

(i) NCBI nr only;

(j) COG2003, COG2008, COG2014, arCOG, arCOG2014, POG2013;

(k) planned in next release;

(l) for Pfam and TIGRfam;

(m) partial feature only;

(n) available as separate software, integration in the next release update;

(o) *Gamola2 creates verbose error logs*.

For example, Prokka delivers extremely fast annotation results, even on a typical desktop computer. Prokka achieves this fast turnaround by focusing on curated databases (e.g., UniProt, Pfam and TIGRfam) and by limiting custom databases to finished bacterial genomes of the same genus. In contrast, GAMOLA2 follows the opposite philosophy by providing as much verbose information for each predicted gene as possible. Rather than implying a given gene annotation, GAMOLA2 aims at creating a comprehensive dataset that enables rapid and confident expert curation.

ConsPred features a novel rule-based algorithm to predict the most accurate gene model, while GAMOLA2 builds an additive gene model that may also include partial genes to provide the most inclusive gene model—acknowledging the inclusion of false positives in the gene model that will then be removed during expert curation. In particular for fragmented draft phase genomes, it is easier and faster to delete false positives from the gene model than to detect, analyse, and add missing genes.

Similarly, RAST/myRAST focus on metabolic network reconstruction and is built on a unique datasets (e.g., FIGfam) and cross-platform access (e.g., SEED database) that highlights biochemical pathways present within bacterial genomes.

Each of these platforms provides a different focus for microbial genome annotations. Which system will ultimately be most suited for a given genome project will depend on the respective requirements at and purpose of the resulting annotation.

## Summary

The GAMOLA2/Artemis ecosystem provides a comprehensive, user-friendly and readily accessible framework for microbiologists to work with and curate draft and completed genomes. Specific emphasis was given to providing functional and structural analyses in a stand-alone environment that does not require remote access or rely on other underlying dependencies (other than the ActivePerl distribution and Java). GAMOLA2 utilizes recognized tools with known performance parameters that are combined into a single source of information. The main output comprises an annotated Genbank file with additional features and descriptive qualifiers that, in combination with the Artemis Genome viewer, create an intuitive and responsive environment to rapidly assess individual database hits for each gene in their totality and create expert curations. The supplemental modules in GAMOLA2 further increase the flexibility for genome annotations and provide assistance for tracking draft phase genomes, and the submission of genomes to depositories.

GAMOLA2 is continuously being developed and new functionality and additional supplemental modules will be integrated based on user-feedback.

## Availability

The GAMOLA2/Artemis distribution is freely available for academic use and can be downloaded from Google Drive (Table 2 lists to respective URLs to download all GAMOLA2 components). The distribution already contains most software tools and some specialized databases. Larger databases and those that are frequently updated require a separate download and can either be downloaded via a snapshot file (Table 2) or manually by following the instructions in the manual. The provided snapshot database file will be updated periodically alongside the GAMOLA2 distribution.

**Table 2:** **Download URLs to the GAMOLA2 distribution**.

**GAMOLA2 software distribution**	**Download link**	**File size [KB]**
Readme	https://drive.google.com/file/d/0B_fIEHIR2oaacWQ1T0Y2dm0wWUk/view?usp=sharing	1
GAMOLA2 Manual (PDF)	https://drive.google.com/file/d/0B_fIEHIR2oaaczBQT2dYQUw4TUE/view?usp=sharing	3,947
GAMOLA2 distribution	https://drive.google.com/file/d/0B_fIEHIR2oaabVlzcF9NUTlnbjQ/view?usp=sharing	1,376,676
Customized Artemis 16	https://drive.google.com/file/d/0B_fIEHIR2oaaM1RndXVpZUl4emc/view?usp=sharing	24,116
GAMOLA2 tutorial dataset	https://drive.google.com/file/d/0B_fIEHIR2oaadVpVZEZuSUZBYkU/view?usp=sharing	5,265
Databases	https://drive.google.com/file/d/0B_fIEHIR2oaaVHlRZmc0cWJBOTA/view?usp=sharing	1,201,277

An example annotation is provided with the distribution package and can be used for training purposes in Artemis.

## Author contributions

EA wrote the GAMOLA2 software, the manual and the manuscript. EA, JL, and AM designed the Artemis modifications. JL programmed the modified version of Artemis. JL and AM reviewed the manual and the manuscript.

### Conflict of interest statement

The authors declare that the research was conducted in the absence of any commercial or financial relationships that could be construed as a potential conflict of interest.

## Supplementary material

Supplemental Figure 1 **Initial system setup, hardware configuration**. Screenshot of the GAMOLA2 graphical user interface (GUI) Systems Setup for hardware configuration and data management. The system can be set-up to re-use or erase existing data from a previous annotation run, the number of CPUs or cores available defined and result data may be sorted into individual parent folders and archived.

Supplemental Figure 2 **Initial system setup, blast properties**. Screenshot of the GAMOLA2 GUI Systems Setup for Blast result refinement. The best Blast result shown in the assembled Genbank file can be filtered for unwanted entries, the maximum number of Blast results displayed and the appropriate translation table be defined, Blast results directly obtained through the COG database can be ignored and a Blast summary (a separate text file) may be created.

Supplemental Figure 3 **Initial system setup, Genbank updates**. Screenshot of the GAMOLA2 GUI Systems Setup for Genbank file updates. Where Genbank files are used as input files, either a new Genbank file may be created based on the analyses selected or the existing file be updated, retaining, or replacing selected features.

Supplemental Figure 4A–C **Initial system setup, custom Genbank, and FASTA headers**. Screenshot of the GAMOLA2 GUI Systems Setup for custom Genbank header configurations. **(A,C)** New Genbank headers can be created (Genbank and FASTA input files) or existing ones re-used (Genbank input files). **(B)** The point-and-click interface to build a new Genbank header. Fields and sub-fields can be selected and field values entered.

Supplemental Figure 5A–C **Gene models and functional analysis options**. Screenshot of the GAMOLA2 GUI main options: **(5A)** Gene models, Blast, COG, PFam, and TIGRfam analyses can be selected individually. Further customisation enables legacy support, the level of verbosity and the number of domains shown in the annotated Genbank file. **(5B)** Supported gene callers currently available to generate an additive gene model. Glimmer 2 or 3 can be chosen alternatively and combined with Prodigal, Critica and an intergenic Blast output. To reduce run-time, intergenic Blast results can be re-used from previous runs, as long as the respective input file remains unchanged. Ribosomal binding sites may be predicted using RBSfinder (Suzek et al., 2001). **(5C)** The Intergenic Blast setup supports default or custom Blast databases for the identification for putative intergenic ORFs (igORFs). The algorithm can be adjusted by setting a minimum igORF length and by how far a predicted ORF may reach into an existing one. igORFs may be determined either based on ORF orientation (i.e., only genes on the sense or anti-sense direction are considered when defining the respective intergenic regions, resulting in two separate igORF predictions) or by flattening the gene model (i.e., genes in both orientations will be considered for the determination of intergenic regions).

Supplemental Figures 6A–D **Structural analyses**. Screenshot of the GAMOLA2 GUI structural analysis options. A range of structural and non-coding analyses can be carried out to supplement and enhance the existing gene model and its annotation. The most relevant for any given analysis can be adjusted to the respective input files. **(6A)** tRNA, rRNA, and non-coding RNAs, **(6B)** transmembrane helices and signal peptide cleavage sites, **(6C)** rho-independent terminator structures and CRISPRs and **(6D)** vector contamination.

Supplemental Figure 7 **Database selection**. Screenshot of the GAMOLA2 GUI database selection options. Training files for Glimmer can be provided or the self-train option be selected. Databases for BLAST, Pfam, and TIGRfam can be selected, multiple databases may be chosen for PFam and TIGRfam analyses. Six different COG databases are currently supported and can be chosen via a drop-down menu.

Supplemental Figure 8 **Configuring input files**. Screenshot of the GAMOLA2 GUI input file configuration page. Dealing with fragmented draft genomes or multiple entry files requires flexibility in the way sequences are associated with each other. GAMOLA2 can concatenate msFASTA and msGenbank files as well as replace internal ambiguities with a non-bleeding spacer sequences, preventing gene callers from creating false positives (left panel). Current input files are shown in the central panel and the directory content can be refreshed on-the-fly. Associated groups of input files can be created that will be concatenated and treated as a single entity, based on the available input sequences (right panel).

Supplemental Figure 9A–C **Genome Annotation in Artemis**. Screenshots of Genbank-embedded and associated information that can be retrieved in Artemis. **(9A)** Annotating CDS and gene features, **(9B)** InterPro and GO information in a TIGRfam feature, **(9C)** COG result data file retrieved with Artemis.

Supplemental Figure 10 **Supplemental modules, custom blast databases**. Screenshot of the GAMOLA2 GUI for creating custom Blast databases. Fasta and Genbank files are accepted as input files and both amino-acid and nucleotide Blast databases can be created. Where desired, relevant intermediary files can be retained.

Supplemental Figure 11 **Supplemental modules, rotating Genbank files**. Screenshot of the GAMOLA2 GUI for rotating Genbank files. This module enables forward and backward rotation of a Genbank file by defining the new start position. This feature is mostly used to align genomes using a common anchor point (e.g., dnaA).

Supplemental Figure 12 **Supplemental modules, prepare for Sequin submission**. Screenshot of the GAMOLA2 GUI for parsing Genbank files for Sequin. This module will generate Sequin (https://www.ncbi.nlm.nih.gov/Sequin/) compliant sequence files, feature tables and AGP files for completed and draft phase genomes.

Supplemental Figure 13 **Supplemental modules, transfer annotation**. Screenshot of the GAMOLA2 GUI for transferring annotation from one genome version to another. Manual curation efforts can be transferred between draft genome versions. Gene and product annotation as well as locus tags can be retained between versions. Separate information files are created to highlight genes that were not identified or were ambiguous (e.g., gene duplications).

Supplemental Figure 14 **Supplemental modules, metagenome analysis**. Screenshot of the GAMOLA2 GUI for a high-level metagenomics analysis. This module is intended to provide an overview of functionality present in a metagenome. Ideally used with custom Blast databases, the output allows to investigate hit frequencies and levels of similarities. Results can then be visualized using dedicated graphing software.

Supplemental Figure 15 **Example of a comparative metagenome analysis**. Three metagenomes (Ciric et al., 2014), representing different fractions of a biological ecosystem, were compared against seven custom functional Blast databases comprising specific enzymes classes (X-axis). Hits of reads against each database were grouped by e-value (Y-axis) and respective frequencies in the metagenomes (Z-axis). Distinct differences can be observed for the number of metagenome reads and their respective levels of similarity against different enzyme classes (e.g., different distribution profiles between the three fractionated metagenomes at an e-value of 0 for arabinosidases, acetyl xylan esterases, and Rubisco). This high-level overview grants insights into possible biological differences between different ecosystems or distinct microbial communities within the same ecological niche.

Supplementary Table 1 **Comparison of metagenomic hits between IMG/M and GAMOLA2 on an unassembled metagenome**.

Supplemental Presentation File 1 **Manual for GAMOLA2**. The manual in PDF format describes in detail the intallation process, and individual features of the GAMOLA2 annotation software. Further, installation and use of the modified Artemis genome browser is explained. Finally, an annotation guide is provided with suggestions on how to efficiently curate and manage (draft) genome annotations.
