# Supplementary figures and images for: GAMOLA2, a Comprehensive Software Package for the Annotation and Curation of Draft and Complete Microbial Genomes
Source: Front Microbiol. 2017 Mar 23;8:346. doi: 10.3389/fmicb.2017.00346 (PMC5362640; doi:10.3389/fmicb.2017.00346)

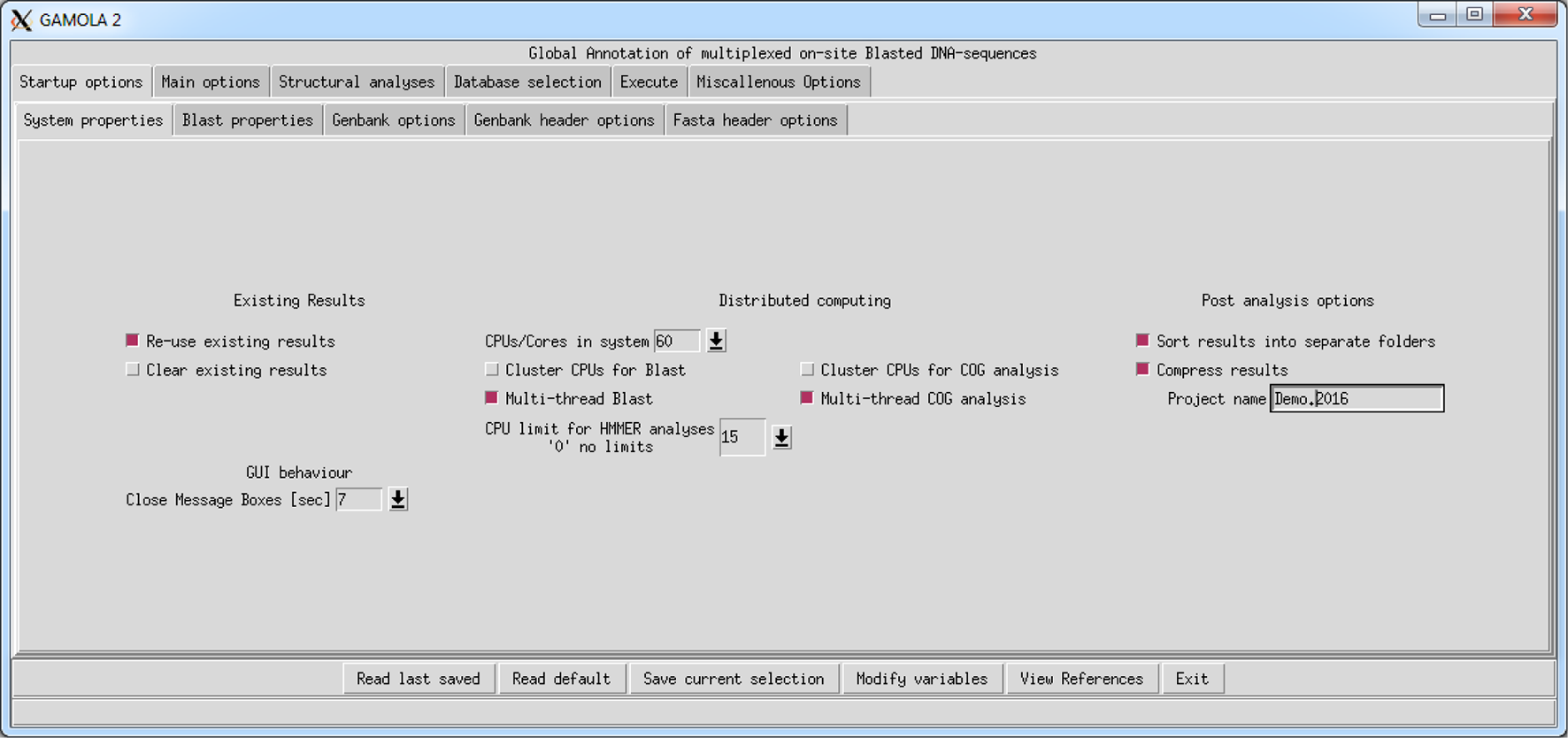

Supplement: Supplemental Figure 1 — Initial system setup, hardware configuration. Screenshot of the GAMOLA2 graphical user interface (GUI) Systems Setup for hardware configuration and data management. The system can be set-up to re-use or erase existing data from a previous annotation run, the number of CPUs or cores available defined and result data may be sorted into individual parent folders and archived. [file Image1.TIF]

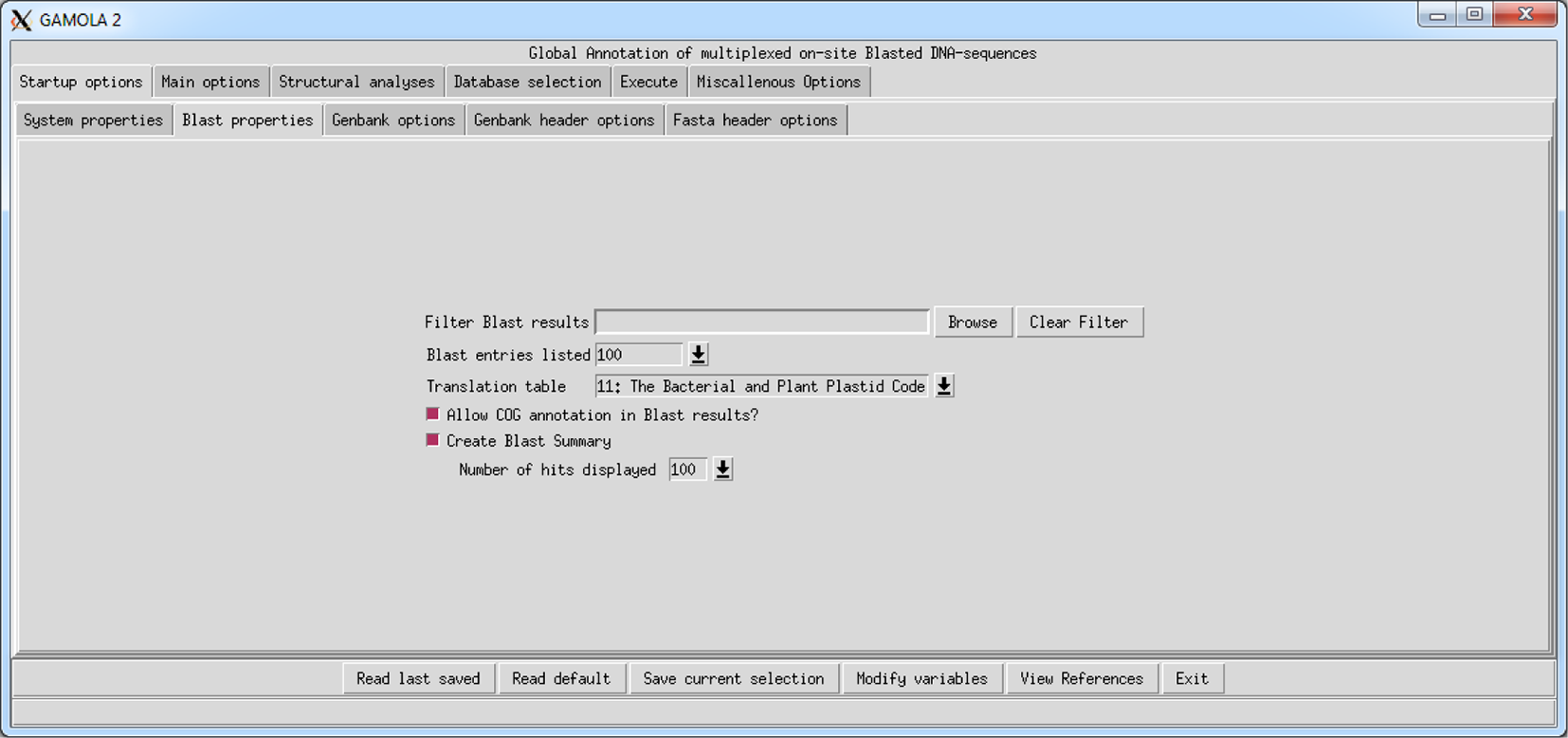

Supplement: Supplemental Figure 2 — Initial system setup, blast properties. Screenshot of the GAMOLA2 GUI Systems Setup for Blast result refinement. The best Blast result shown in the assembled Genbank file can be filtered for unwanted entries, the maximum number of Blast results displayed and the appropriate translation table be defined, Blast results directly obtained through the COG database can be ignored and a Blast summary (a separate text file) may be created. [file Image2.TIF]

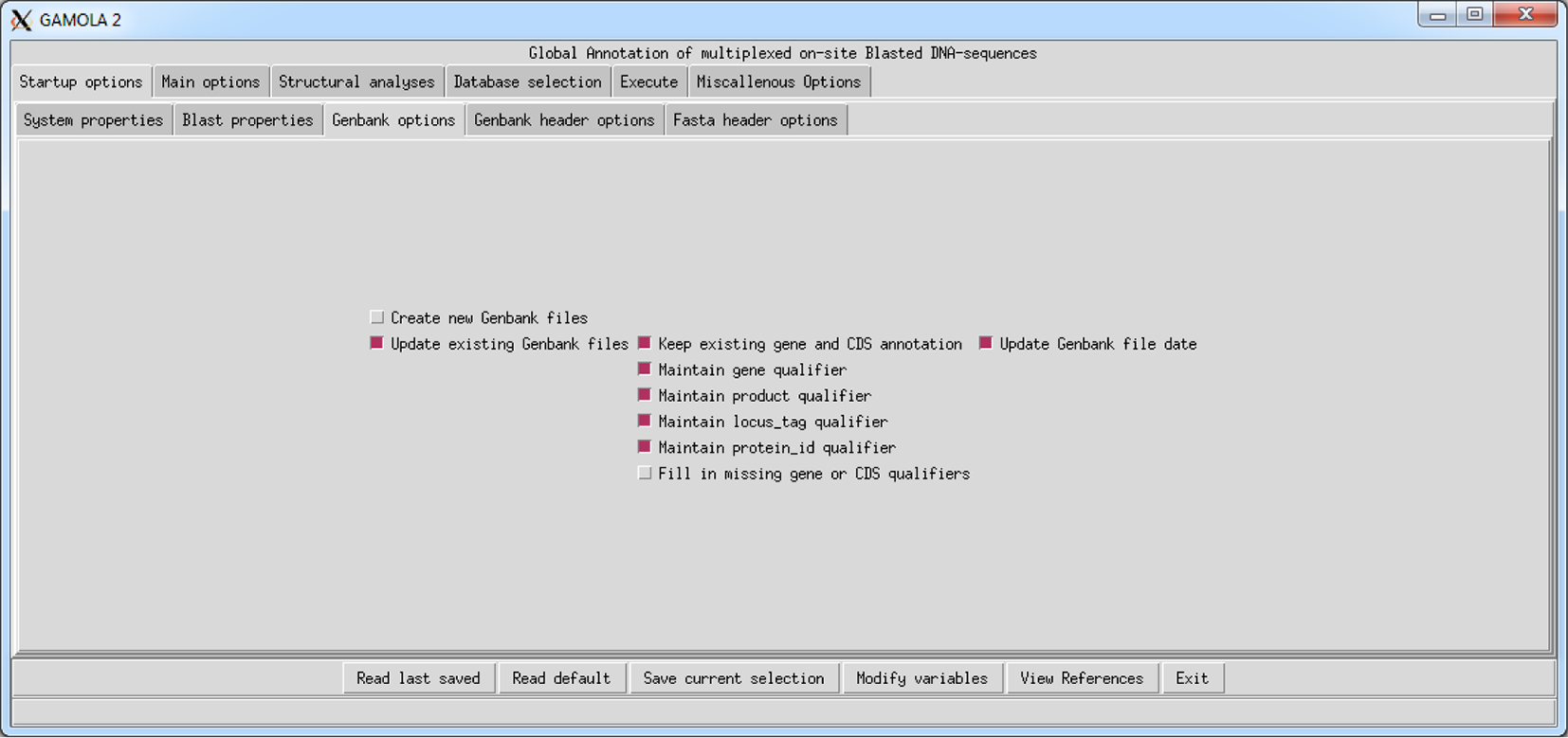

Supplement: Supplemental Figure 3 — Initial system setup, Genbank updates. Screenshot of the GAMOLA2 GUI Systems Setup for Genbank file updates. Where Genbank files are used as input files, either a new Genbank file may be created based on the analyses selected or the existing file be updated, retaining, or replacing selected features. [file Image3.TIF]

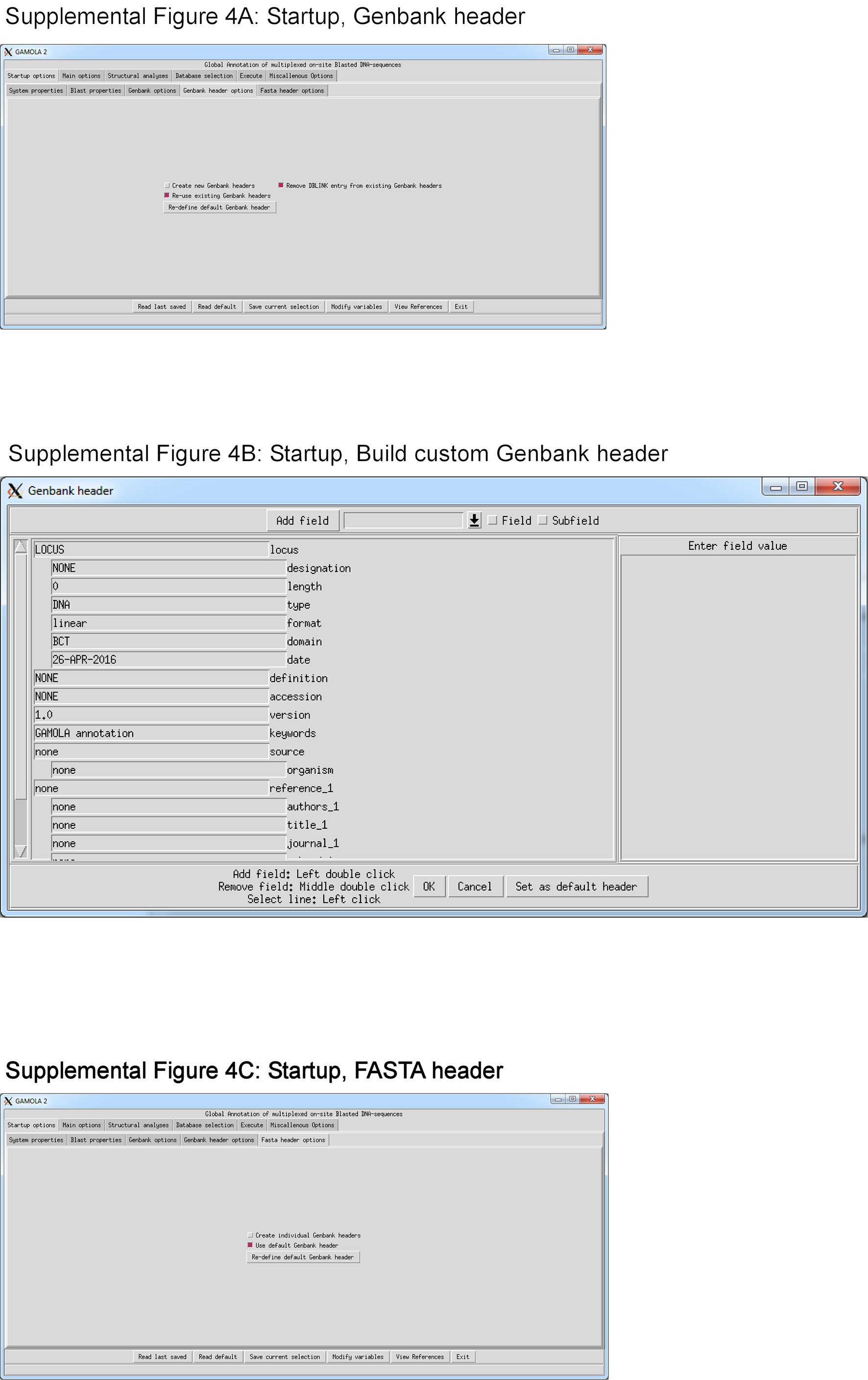

Supplement: Supplemental Figure 4A–C — Initial system setup, custom Genbank, and FASTA headers. Screenshot of the GAMOLA2 GUI Systems Setup for custom Genbank header configurations. (A,C) New Genbank headers can be created (Genbank and FASTA input files) or existing ones re-used (Genbank input files). (B) The point-and-click interface to build a new Genbank header. Fields and sub-fields can be selected and field values entered. [file Image4.tif]

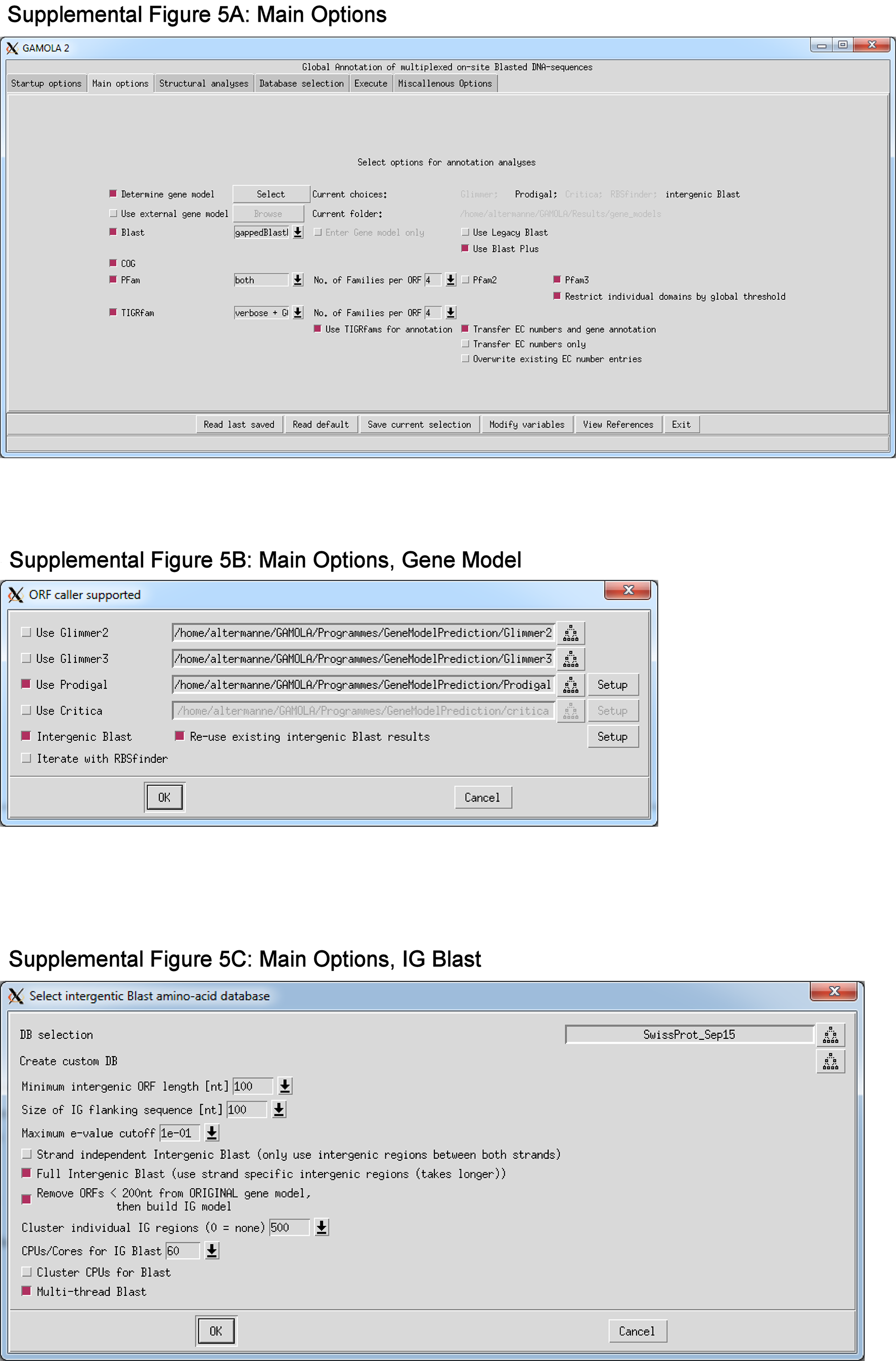

Supplement: Supplemental Figure 5A–C — Gene models and functional analysis options. Screenshot of the GAMOLA2 GUI main options: (5A) Gene models, Blast, COG, PFam, and TIGRfam analyses can be selected individually. Further customisation enables legacy support, the level of verbosity and the number of domains shown in the annotated Genbank file. (5B) Supported gene callers currently available to generate an additive gene model. Glimmer 2 or 3 can be chosen alternatively and combined with Prodigal, Critica and an intergenic Blast output. To reduce run-time, intergenic Blast results can be re-used from previous runs, as long as the respective input file remains unchanged. Ribosomal binding sites may be predicted using RBSfinder (Suzek et al., 2001). (5C) The Intergenic Blast setup supports default or custom Blast databases for the identification for putative intergenic ORFs (igORFs). The algorithm can be adjusted by setting a minimum igORF length and by how far a predicted ORF may reach into an existing one. igORFs may be determined either based on ORF orientation (i.e., only genes on the sense or anti-sense direction are considered when defining the respective intergenic regions, resulting in two separate igORF predictions) or by flattening the gene model (i.e., genes in both orientations will be considered for the determination of intergenic regions). [file Image5.tif]

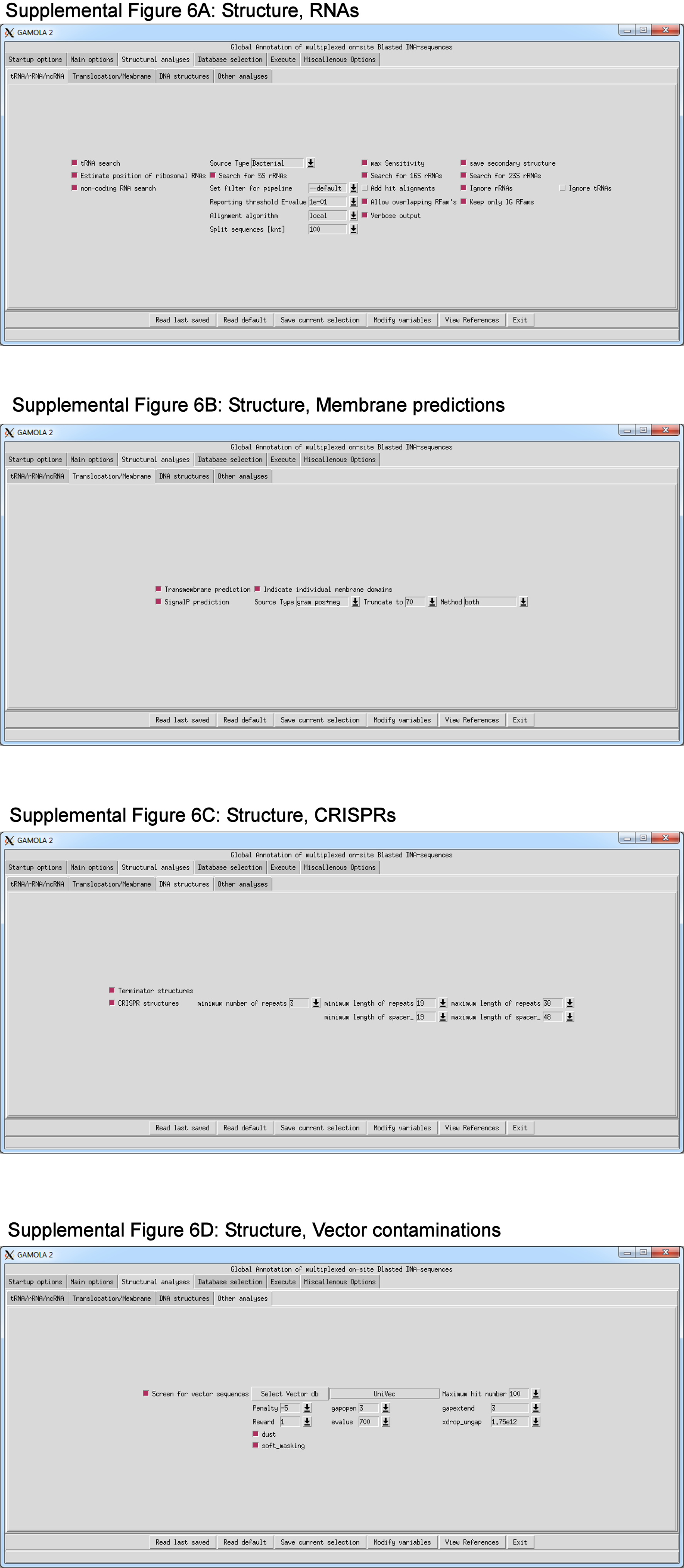

Supplement: Supplemental Figures 6A–D — Structural analyses. Screenshot of the GAMOLA2 GUI structural analysis options. A range of structural and non-coding analyses can be carried out to supplement and enhance the existing gene model and its annotation. The most relevant for any given analysis can be adjusted to the respective input files. (6A) tRNA, rRNA, and non-coding RNAs, (6B) transmembrane helices and signal peptide cleavage sites, (6C) rho-independent terminator structures and CRISPRs and (6D) vector contamination. [file Image6.tif]

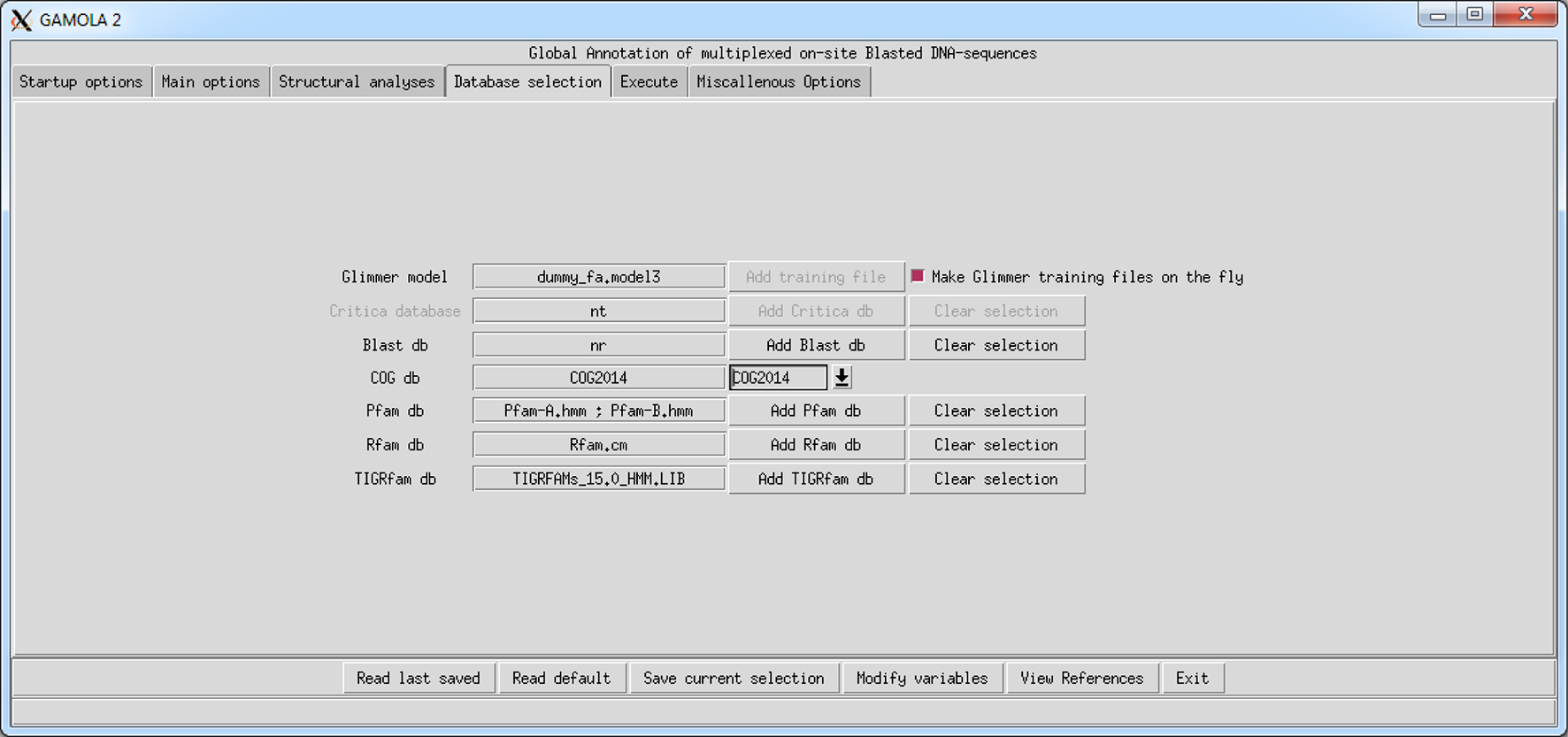

Supplement: Supplemental Figure 7 — Database selection. Screenshot of the GAMOLA2 GUI database selection options. Training files for Glimmer can be provided or the self-train option be selected. Databases for BLAST, Pfam, and TIGRfam can be selected, multiple databases may be chosen for PFam and TIGRfam analyses. Six different COG databases are currently supported and can be chosen via a drop-down menu. [file Image7.tif]

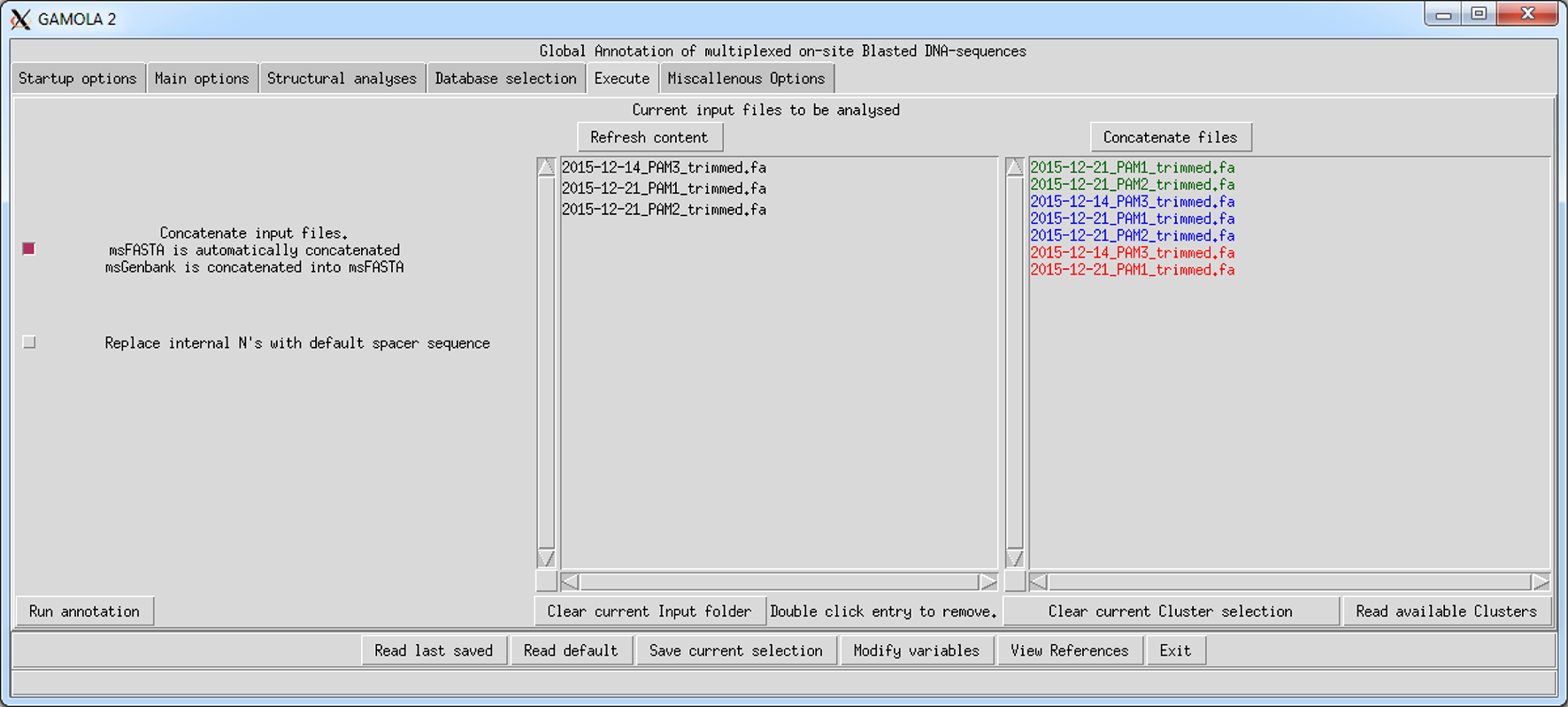

Supplement: Supplemental Figure 8 — Configuring input files. Screenshot of the GAMOLA2 GUI input file configuration page. Dealing with fragmented draft genomes or multiple entry files requires flexibility in the way sequences are associated with each other. GAMOLA2 can concatenate msFASTA and msGenbank files as well as replace internal ambiguities with a non-bleeding spacer sequences, preventing gene callers from creating false positives (left panel). Current input files are shown in the central panel and the directory content can be refreshed on-the-fly. Associated groups of input files can be created that will be concatenated and treated as a single entity, based on the available input sequences (right panel). [file Image8.tif]

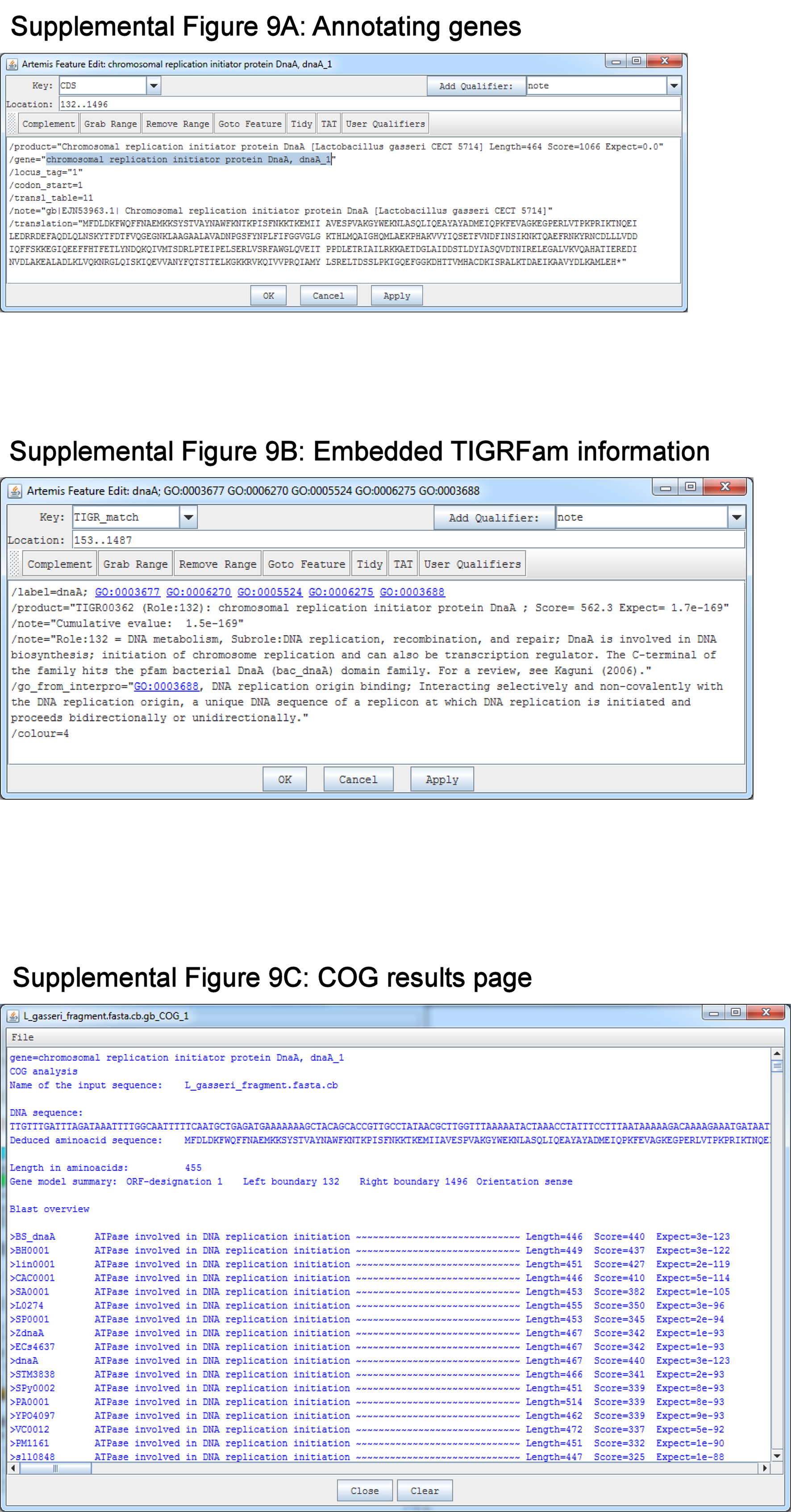

Supplement: Supplemental Figure 9A–C — Genome Annotation in Artemis. Screenshots of Genbank-embedded and associated information that can be retrieved in Artemis. (9A) Annotating CDS and gene features, (9B) InterPro and GO information in a TIGRfam feature, (9C) COG result data file retrieved with Artemis. [file Image9.tif]

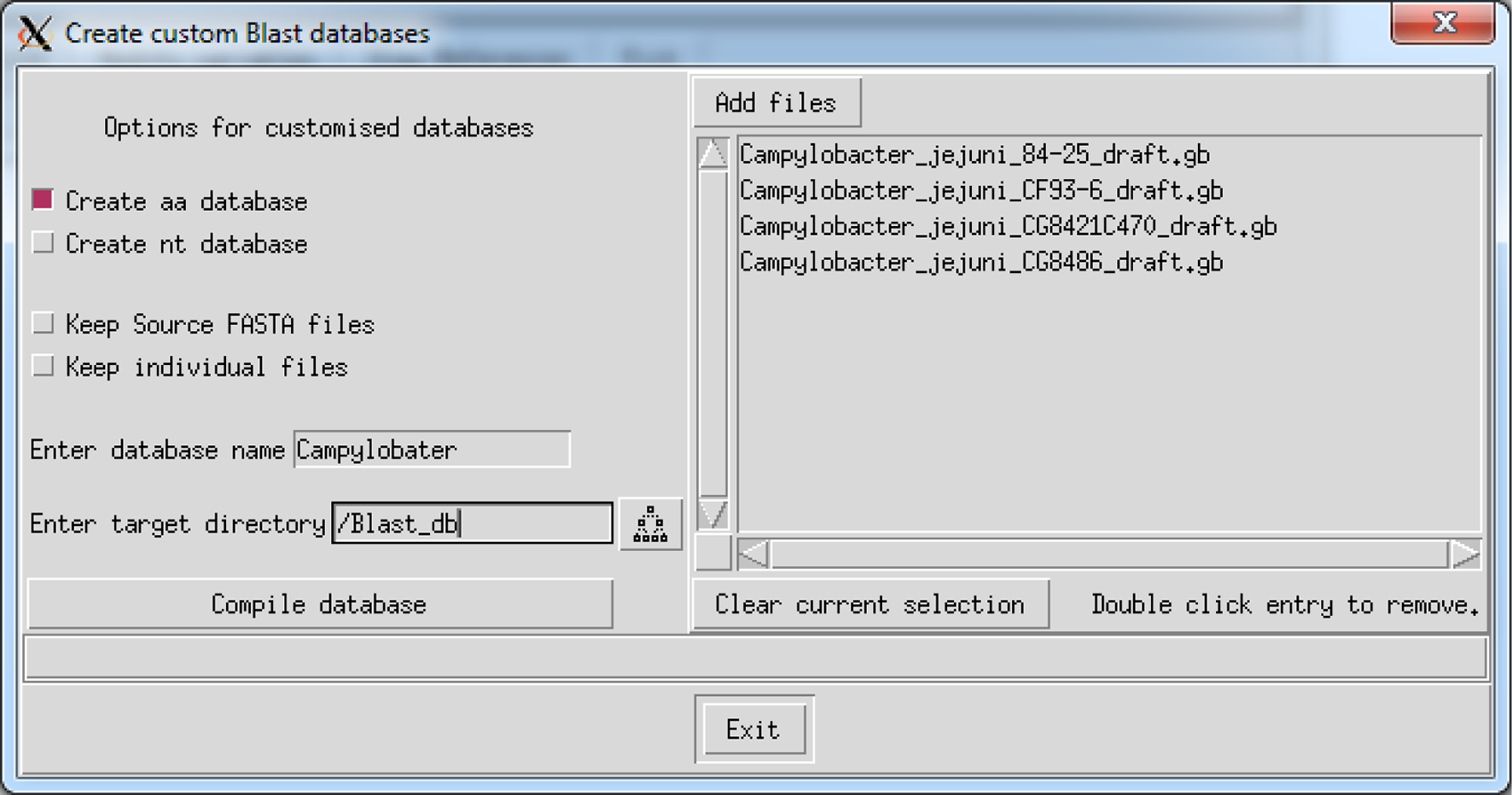

Supplement: Supplemental Figure 10 — Supplemental modules, custom blast databases. Screenshot of the GAMOLA2 GUI for creating custom Blast databases. Fasta and Genbank files are accepted as input files and both amino-acid and nucleotide Blast databases can be created. Where desired, relevant intermediary files can be retained. [file Image10.tif]

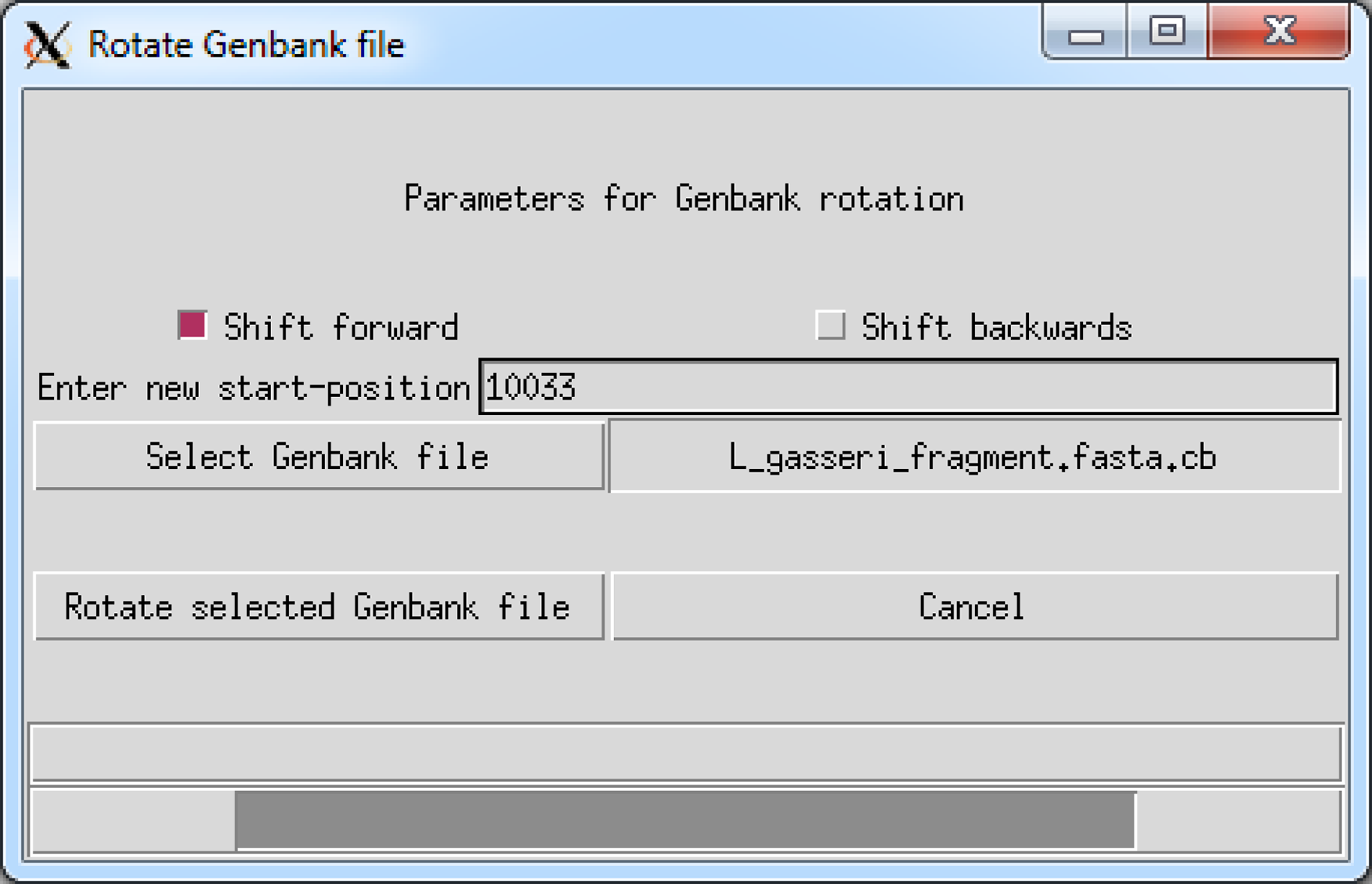

Supplement: Supplemental Figure 11 — Supplemental modules, rotating Genbank files. Screenshot of the GAMOLA2 GUI for rotating Genbank files. This module enables forward and backward rotation of a Genbank file by defining the new start position. This feature is mostly used to align genomes using a common anchor point (e.g., dnaA). [file Image11.tif]

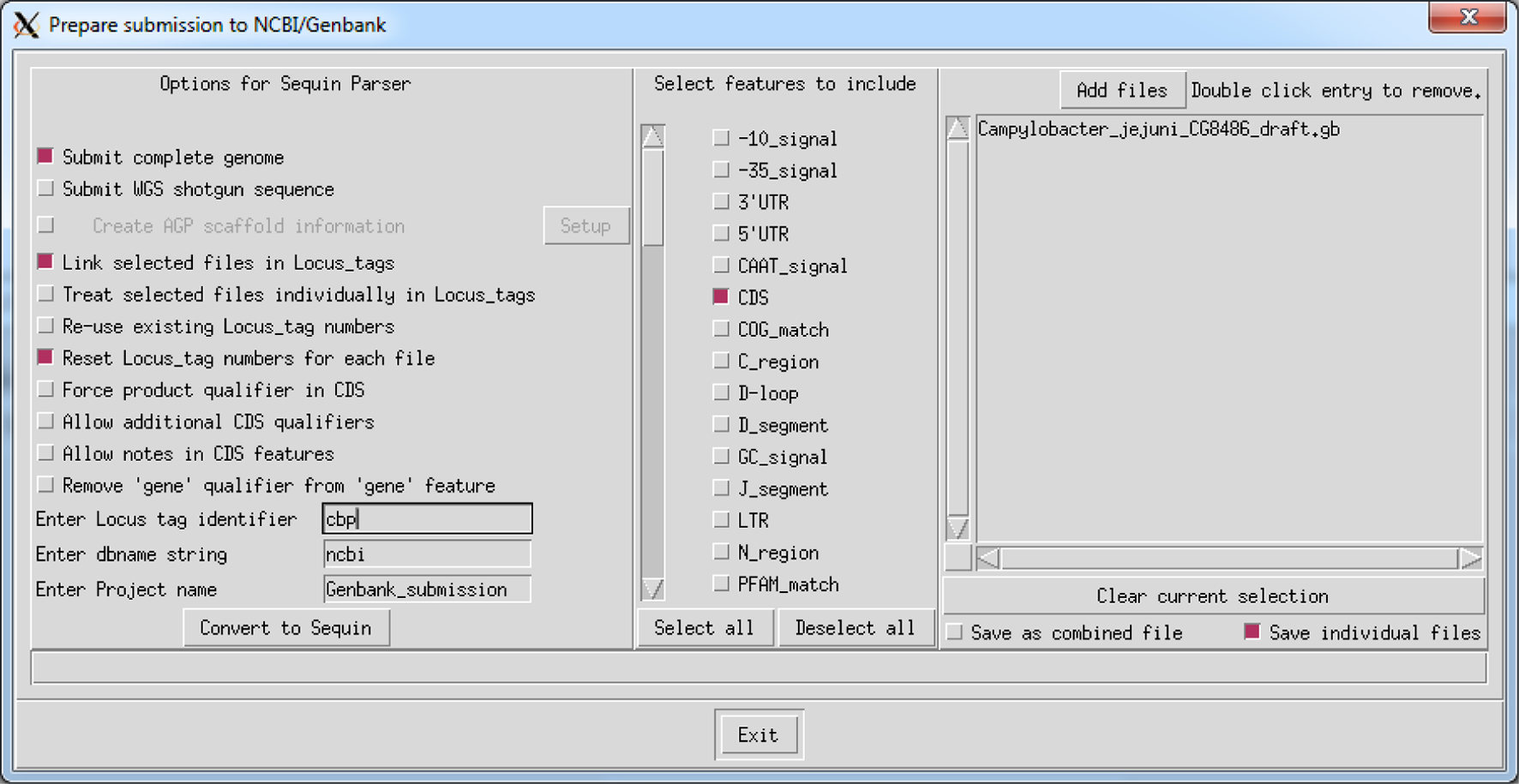

Supplement: Supplemental Figure 12 — Supplemental modules, prepare for Sequin submission. Screenshot of the GAMOLA2 GUI for parsing Genbank files for Sequin. This module will generate Sequin (https://www.ncbi.nlm.nih.gov/Sequin/) compliant sequence files, feature tables and AGP files for completed and draft phase genomes. [file Image12.tif]

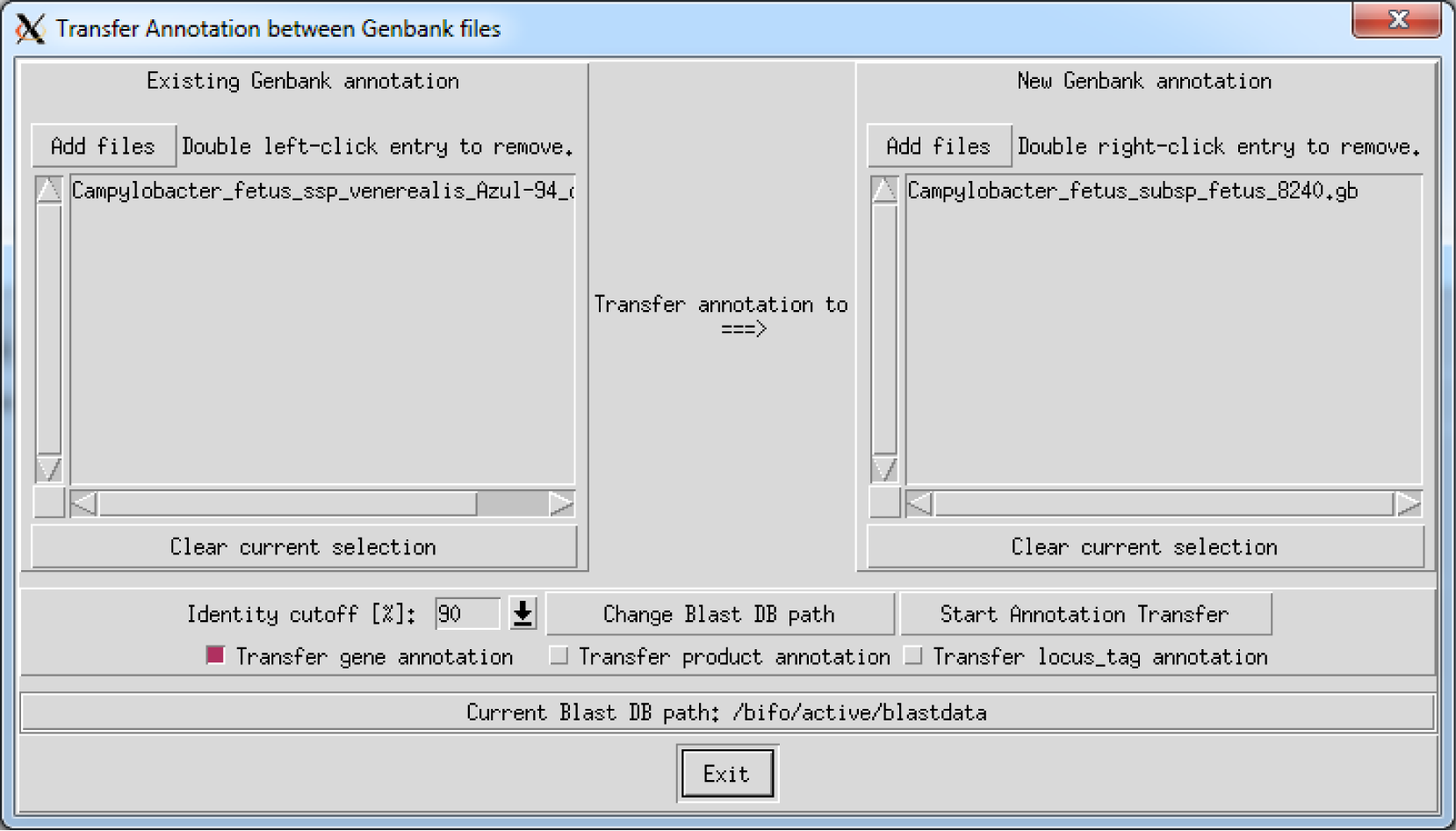

Supplement: Supplemental Figure 13 — Supplemental modules, transfer annotation. Screenshot of the GAMOLA2 GUI for transferring annotation from one genome version to another. Manual curation efforts can be transferred between draft genome versions. Gene and product annotation as well as locus tags can be retained between versions. Separate information files are created to highlight genes that were not identified or were ambiguous (e.g., gene duplications). [file Image13.tif]

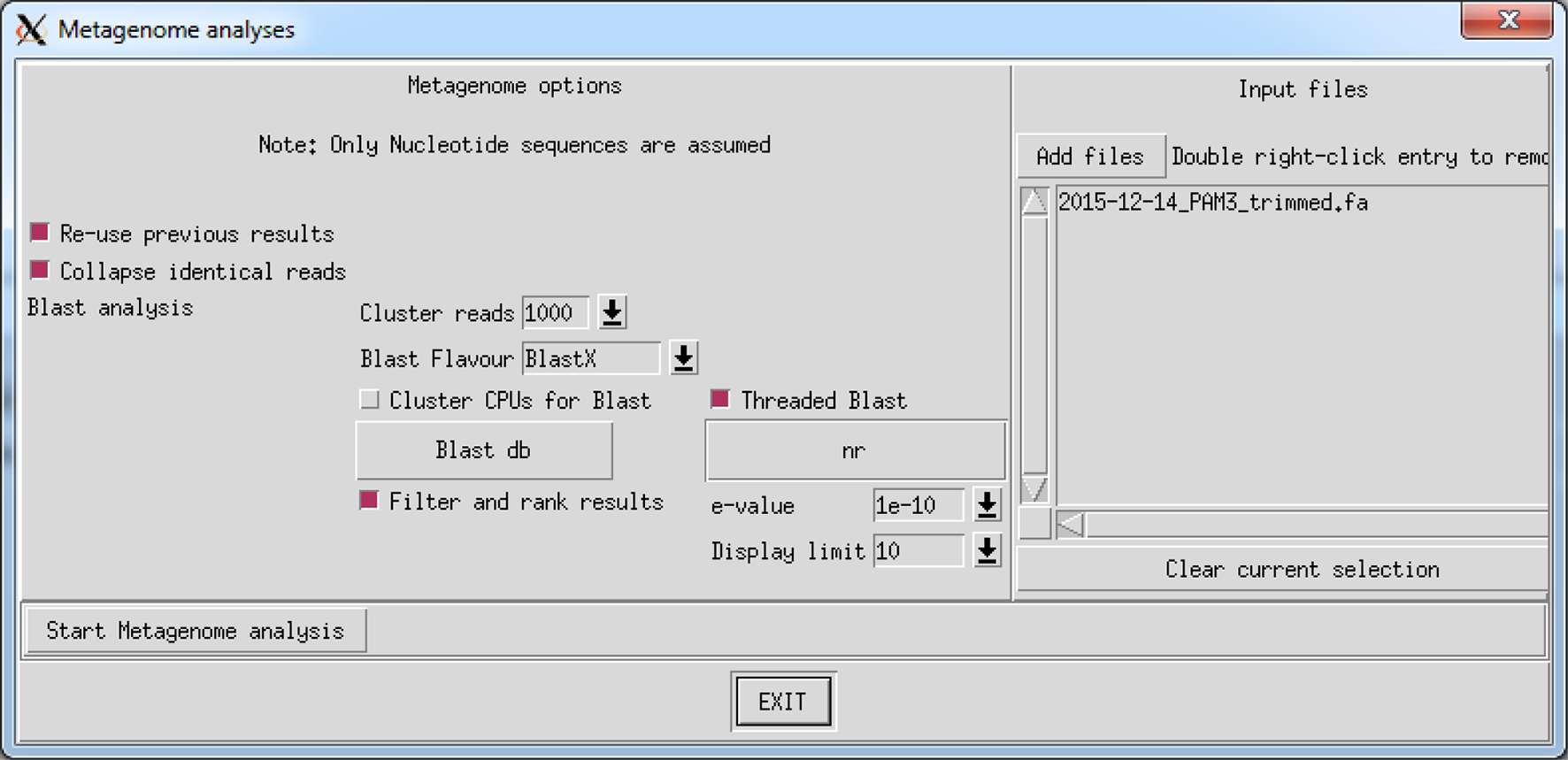

Supplement: Supplemental Figure 14 — Supplemental modules, metagenome analysis. Screenshot of the GAMOLA2 GUI for a high-level metagenomics analysis. This module is intended to provide an overview of functionality present in a metagenome. Ideally used with custom Blast databases, the output allows to investigate hit frequencies and levels of similarities. Results can then be visualized using dedicated graphing software. [file Image14.tif]
