# Supplementary material for: GAMOLA2, a Comprehensive Software Package for the Annotation and Curation of Draft and Complete Microbial Genomes
Source: Front Microbiol. 2017 Mar 23;8:346. doi: 10.3389/fmicb.2017.00346 (PMC5362640; doi:10.3389/fmicb.2017.00346)
Supplement: Supplementary Table 1 — Comparison of metagenomic hits between IMG/M and GAMOLA2 on an unassembled metagenome. [file Table1.PDF]

Supplemental Table 1: Comparison of metagenomic hits between IMG/M and GAMOLA2 on an unassembled metagenome.

| | IMG/M | 722/723 Associated | 722 Adherent | 723 Adherent |
|---|---|---|---|---|
| Cellulases | 388 | 453 | 708 | 479 |
| Xylanases | 259 | 207 | 304 | 218 |
| Arabinosidases | 62 | 284 | 434 | 283 |
| Glycosylhydrolases | 531 | 717 | 1119 | 728 |
| RuBisCO | 6 | 38 | 74 | 48 |

The e-value threshold for GAMOLA2 was set a 1e-50.

IMG/M analysis depicts only the combined 722–723 adherent metagenome.
