# Supplementary material for: GAMOLA2, a Comprehensive Software Package for the Annotation and Curation of Draft and Complete Microbial Genomes
Source: Front Microbiol. 2017 Mar 23;8:346. doi: 10.3389/fmicb.2017.00346 (PMC5362640; doi:10.3389/fmicb.2017.00346)
Supplement: Supplemental Presentation File 1 — Manual for GAMOLA2. The manual in PDF format describes in detail the intallation process, and individual features of the GAMOLA2 annotation software. Further, installation and use of the modified Artemis genome browser is explained. Finally, an annotation guide is provided with suggestions on how to efficiently curate and manage (draft) genome annotations. [file Presentation1.PDF]

GAMOLA 2 – a microbial annotation and visualisation suite

Instruction Manual

Contents

1 Introduction 5

2 Installation of GAMOLA2 6

2.1 License Requirements 6

2.2 Step-by-step installation guide 7

(1) Download the GAMOLA2 distribution files 7

(2) Extract the GAMOLA2 archive 8

(3) Install the ActivePerl distribution. 8

(4) Initialisation of GAMOLA2 (commercial users) 9

(5) Initialisation of GAMOLA2 (academic users) 9

(6) Setup of local databases 10

(7) Final steps 12

3 Running a GAMOLA2 annotation 13

3.1 Design concept of GAMOLA2 GUI 13

3.2 Startup options 13

3.2.1 System properties 13

3.2.2 Blast Properties 16

3.2.3 Genbank options 18
3.2.4 Genbank header options 19
3.2.5 Fasta header options 21
3.3 Main options 22
3.3.1 Determine gene model: 22
3.3.2 Use external gene model 26
3.3.3 Blast 26
3.3.4 COG 27
3.3.5 Pfam 28
3.3.6 TIGRfam 29
3.4 Structural Analyses 30
3.4.1 tRNA/rRNA/ncRNA 30
3.4.2 Protein Translocation/Membrane proteins 34
3.4.3 DNA structures 36
3.4.4 Other analyses 37
3.5 Database selection 39
3.5.1 ‘Glimmer model’: 39
3.5.2 ‘Critica Database’: 39
3.5.3 ‘Blast Database’ 39
3.5.4 ‘COG Database’ 40
3.5.5 ‘Pfam Database’ 40
3.5.6 ‘Rfam database’ 40

3.5.7 'TIGRfam database' ... 40

3.6 Execute ... 41

3.6.1 Input sequences ... 42

3.6.2 Concatenate input files ... 43

3.6.3 Start the Annotation Run ... 46

4 GAMOLA2 output ... 47

4.1 The Genbank file ... 47

4.2 Genbank associated files ... 47

4.3 Genbank associated data directories ... 48

4.4 Results sorted into individual directories ... 48

4.5 Archive files ... 48

5 Recommended Genome Browser ... 49

5.1 Installation of Artemis ... 49

5.1.1 Quick Guide to increase Java memory allocation (for Windows machines): ... 50

5.2 Working with Artemis and a GAMOLA annotated sequence ... 51

5.2.1 Color codes for recognised GAMOLA2 features: ... 51

5.2.2 Feature Overview ... 52

5.2.3 Retrieving underlying Results ... 69

6 Additional GAMOLA2 functionality ... 72

6.1 Databases ... 72

6.1.1 Create Custom databases ... 72

6.2 Genbank files ... 75

6.2.1 Rotate single Genbank file 75

6.2.2 Prepare Genbank files for Sequin submission 77

6.3 Annotation 86

6.3.1 Transfer annotation between Genbank files 86

6.4 Metagenome 90

6.4.1 Metagenome analysis 90

7 Annotation Guidelines (suggestion only) 98

1 Introduction

The first version of GAMOLA was published in 2003 (1) and was designed to provide locally annotated and easily accessible microbial genomes. Over time the number of available and useful analyses for functional microbial genome annotation has increased and necessitated an updated version of GAMOLA. GAMOLA2 is a complete re-write of the original software package and now features a graphical user interface, includes a much larger number of available analyses and provides a range of post-annotation tools.

The basic premise for GAMOLA2, however, remains unchanged: to provide a system that comprehensively annotates draft and completed microbial genomes locally (i.e. completely offline) generating GenBank files and associated data that can be analysed without the need to be online and use only minimal computer requirements. To achieve the latter, the Artemis genome browser (14) was chosen for genome visualisation and further enhanced to display new custom GAMOLA2 features and retrieve underlying raw data for each predicted open reading frame (ORF).

The modular architecture of GAMOLA2 facilitates the incorporation of new functional modules to further add information to the annotation. GAMOLA2 is written entirely in PERL and requires the presence of the PERL interpreter. The software has been tested extensively on several Unix systems using ActivePerl distribution 5.8.8.822.

The customised version of the Artemis genome viewer (14) requires Java to be installed.

GAMOLA2 is free for academic non-profit use. For commercial application a licence is required, please contact the author for further information (eric.altermann@agresearch.co.nz). Similarly, most software packages incorporated into GAMOLA2 are available for free, but some require licenses to be used. A list of external tools is listed below – please check which licence agreements are required for your respective circumstances.

Nothing is ever perfect and if you find any bugs or have suggestions to improve or extend the GAMOLA2 functionality, please contact me under eric.altermann@agresearch.co.nz.

Happy Annotations, 19 January 2017

2 *Installation of GAMOLA2*

GAMOLA2 runs entirely off-line which makes it an ideal system for unpublished and/or confidential sequence information. This approach however, necessitates that tools and databases are present locally and thus need to be downloaded from the appropriate sources before the first analysis.

2.1 *License Requirements*

Before GAMOLA2 is installed on your system, please make sure that you have checked and obtained licenses for the external software tools used. Do NOT use GAMOLA2 without those licenses. Here is the list of tools to check for:

Software tool	Contact Information	Reference
BLAST & BLAST+	http://blast.ncbi.nlm.nih.gov/Blast.cgi?CMD=Web&PAGE_TYPE=BlastDocs&DOC_TYPE=Download	(2, 5)
Critica	http://www.ttaxus.com/files/99critica.pdf	(3)
CRISPR Recognition Tool	http://www.ncbi.nlm.nih.gov/pmc/articles/PMC1924867/	(4)
Glimmer 2 & 3	https://ccb.jhu.edu/software/glimmer/	(6)
HMMER 2 & 3	http://hmmer.janelia.org/	(8)
Infernal	http://infernal.janelia.org/	(9)
Prodigal	https://github.com/hyattpd/prodigal/releases/	(10)
SignalP	http://www.cbs.dtu.dk/services/SignalP-3.0/<br>http://www.cbs.dtu.dk/cgi-bin/nph-sw_request?signalp	(7)
TMHMM	http://www.cbs.dtu.dk/services/TMHMM-2.0/	(11)
TransTerm	http://transterm.cbcb.umd.edu/	(12)
tRNAscan-SE	http://lowelab.ucsc.edu/tRNAscan-SE/	(13)

It is the users' responsibility to ensure all required licenses have been obtained.

For academic use, the distribution contains already most installation packages, but individual licences must still be obtained where necessary. Commercial users will have to obtain the tools manually.

Note 1: SignalP and TMHMM installation files must be obtained via the links shown above and copied into the '/lib/Archives' folder after unpacking the GAMOLA2 distribution and before the first

installation. Expected file names are 'tmhmm.rar' and 'signalp-3.0.Linux.tar.Z', 'signalp-4.1b.Linux.tar.Z', or 'signalp-4.1e.Linux.tar.Z'.

Note 2: The tool CRT requires that Java is present. Most systems have Java installed by default, but if not, you must install it manually (e.g. 'sudo yum –y install java').

Note 3: Glimmer 2 requires the g++ compiler to be present. If you experience an error message during the initial installation of GAMOLA2 that Glimmer 2 could not compile, it is most likely due to the absence of g++. You can easily install the compiler via 'sudo yum -y install gcc-c++'. After installation of g++, restart the installation of GAMOLA2.

Note 4: Legacy Blast may require a specific library to run. If you receive an error message similar to "...bad ELF interpreter", please install the glibc.i686 library (i.e. sudo yum –y install glibc.i686)

2.2 *Step-by-step installation guide*

The following is a step-by-step guide for a the installation of GAMOLA2 on your *nix system. GAMOLA2 detects and supports both 32 and 64bit versions of tools, depending on your OS.

(1) *Download the GAMOLA2 distribution files*

Download the GAMOLA2 distribution and related files.

The manual, the GAMOLA2 distribution and the Customised Artemis16 archives are required downloads.

The tutorial dataset and the database files are optional, but highly recommended.

The database archive harbors most databases that change frequently (with the exception of NCBI's nr/nt database) and will be updated frequently. Databases included are: PFam, RFam, TIGRFam, the Interpro descriptor and the SwissProt Blast database.

The table below lists the respective download links for the GAMOLA2 distribution files.

**GAMOLA2 software distribution**	**Download link**	**File size [KB]**
Readme	https://drive.google.com/file/d/0B_fIEHIR2oaacWQ1T0Y2dm0wWUk/view?usp=sharing	1

GAMOLA2 Manual (PDF)	https://drive.google.com/file/d/0B_fIEHIR2oaaczBQT2dYQUw4TUE/view?usp=sharing	3,829
GAMOLA2 distribution	https://drive.google.com/file/d/0B_fIEHIR2oaabVlzcF9NUTlnbjQ/view?usp=sharing	1,376,676
Customised Artemis 16	https://drive.google.com/file/d/0B_fIEHIR2oaaM1RndXVpZUl4emc/view?usp=sharing	24,116
GAMOLA2 tutorial dataset	https://drive.google.com/file/d/0B_fIEHIR2oaadVpVZEZuSUZBYkU/view?usp=sharing	5,265
Databases	https://drive.google.com/file/d/0B_fIEHIR2oaaVHlRZmc0cWJBOTA/view?usp=sharing	1,201,277

(2) ***Extract the GAMOLA2 archive***

Extract the GAMOAL2 rar file into a directory of your choice.

unrar x Gamola2.rar

By default you should see

the "Gamola_xx.pl" perl script,

the default setup file "Gamola.default" and

the 'lib' directory.

In case unrar has not been installed yet, you can obtain it as a free rpm (e.g. https://www.rpmfind.net/linux/rpm2html/search.php?query=unrar) for most systems or you can use 'yum' or 'dnf' to install it directly (e.g. 'sudo yum install unrar').

(3) ***Install the ActivePerl distribution.***

ActivePerl 5.8.8.822 is provided as a tarball and can be found in the '/lib/Archive' directory. Alternatively, you can install version 5.8.8.824 as an rpm package. Install ActivePerl according to the instructions found within the archive or ask your sysadmin to do this for you. ActivePerl has a range of standard Perl modules already included. Use of a different (Active)Perl distribution may require you to manually install any missing modules.

Extract the .tar.gz archive

change into the created directory

type ‘sudo sh install.sh’ and follow the instructions

(4) *Initialisation of GAMOLA2 (commercial users)*

Academic users can skip this step, but may need to check licence requirements.

Obtain all required software tools and copy them into the ‘./lib/Archives’ folder. Continue with step (5).

(5) *Initialisation of GAMOLA2 (academic users)*

During the first time GAMOLA2 is started up, a number of variables and directories will be defined and created. Further, external tools will be compiled and made available to GAMOLA2. This initialisation happens only once and can take a few minutes, depending on the system used.

(a) Start up GAMOLA2 with “*perl Gamola_xx.pl*”, whereby ‘xx’ represents the current version number. You may have to specify the path to ActivePerl (i.e. /opt/ActivePerl/bin/perl) to ensure the correct Perl distribution is invoked.

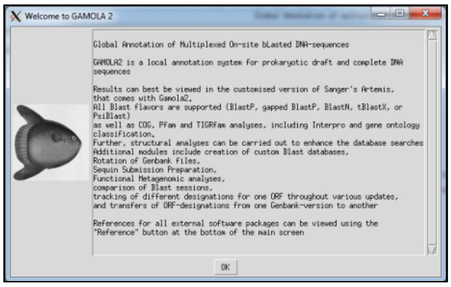

(b) Click “ok” on the welcome screen.

(c) Click on the “Modify variables” button on the bottom row, a new window will pop-up. At any time now or later on you can change threshold values for Blast, COG, Pfam and TIGRfam analyses, as well as modify the location of directories.

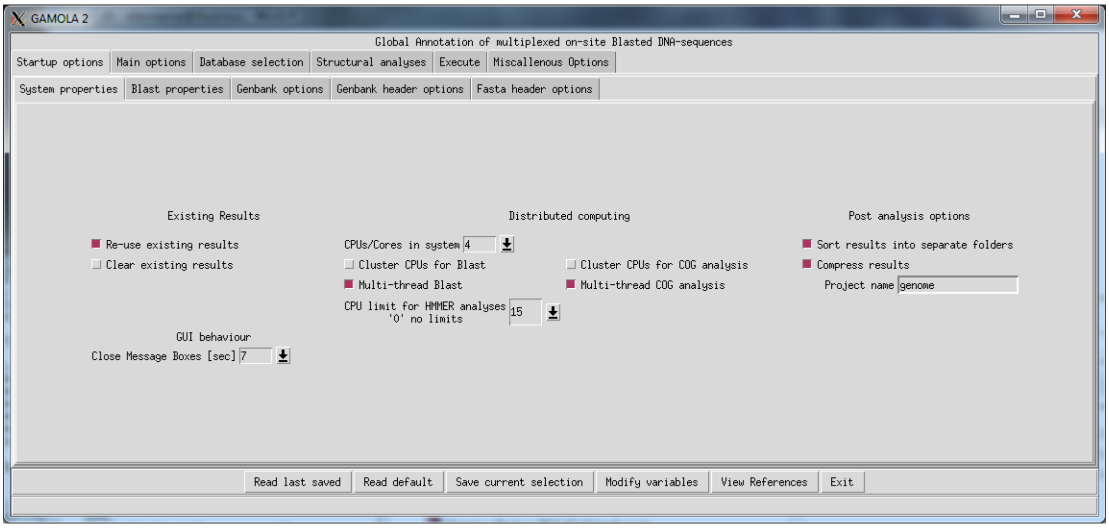

(d) Click on the “Generate Standard Setup” button.

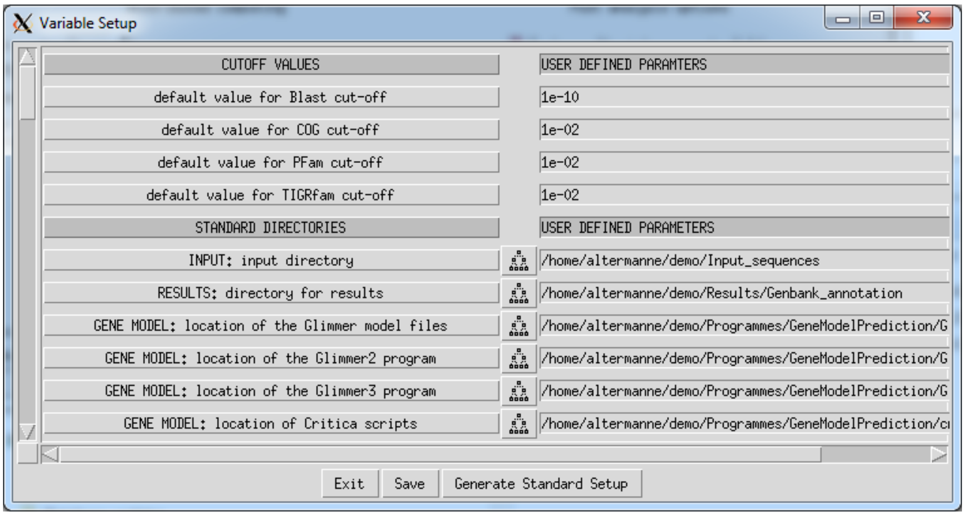

(e) In the new pop-up window, select either “Custom” or “Default” for directory locations. Unless there is a real good reason, I recommend going ahead with the default setup.

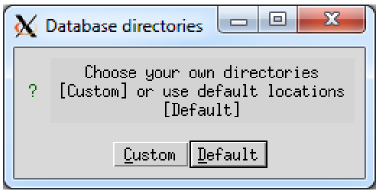

Wait a few minutes while various programmes are uncompressed and compiled. Under some circumstances the compilation of those programmes may fail and where possible a pre-compiled static version will be used instead. If this is the case, execute a test run to ensure that these precompiled programmes do run on your system.

(f) At the end of the compilation process, click “Save” to make all changes permanent in the default GAMOLA variable file.

(g) Change threshold values for Blast, COG, Pfam and TIGRfam to your requirements and click on “Save” and “Save” again.

(6) *Setup of local databases*

GAMOLA2 requires the presence of all relevant databases in the respective local directories. The following section will provide instructions on how to obtain and install these databases. Some smaller databases (e.g. UniVec, COGs, Rfam) are already included in the distribution and will be installed automatically.

Required databases can either be downloaded as part of the GAMOLA2 distribution (via the ‘databases.rar’ file) or downloaded and compiled individually.

If the ‘databases.rar’ file was downloaded (it is expected to be located in the same directory as GAMOLA2), GAMOLA2 will check for the presence of respective databases and, when necessary, extract the ‘databases.rar’ archive and install the individual databases automatically.

If the databases are downloaded individually, then follow the steps below:

(a) **Blast**:

Go to ‘ftp://ftp.ncbi.nlm.nih.gov/blast/db’ and download databases of choice. Minimum recommended is the ‘nr’ database. Copy files into the ‘/Blast_db’ folder.

(b) **COG**:

Six COG databases are installed by default: COG2003 (original), COG2008, COG2014, archaeal COGs, updated archaeal COGs2014 and phage COGs (POG) 2013.

(c) **Pfam**:

Go to ‘ftp://ftp.ebi.ac.uk/pub/databases/Pfam/releases’ and select the latest release. At the time of writing, this is ‘Pfam27.0’.

Download ‘Pfam-A.hmm.gz’ and ‘Pfam-B.hmm.gz’ files, uncompress and use ‘hmmerpress’ to build the indexed Pfam databases.

Download ‘relnotes.txt’.

Go to ‘ftp://ftp.ebi.ac.uk/pub/databases/Pfam/releases/Pfam27.0/database_files’ (use appropriate version number) and download ‘pfamA.txt.gz’ and ‘pfamB.txt.gz’ files. Uncompress.

Copy all Pfam files into the ‘/Pfam_db’ folder.

(d) **TIGRFam**:

Go to ‘ftp://ftp.jcvi.org/pub/data/TIGRFAMs/‘ and download ‘TIGRFAMs_15.0_HMM.LIB.gz’ (use latest version), uncompress, and use ‘hmmpress’ to create the indexed database.

Download ‘TIGRFAMs_15.0_INFO.tar.gz’, uncompress and copy files into a folder named ‘TIGRinfo’.

Download ‘TIGR_ROLE_NAMES’, ‘TIGRFAMS_ROLE_LINK’, ‘TIGRFAMS_GO_LINK’, and ‘RELEASE_NOTE_15.0’ (use appropriate version number).

Go to ‘ftp://ftp.geneontology.org/pub/godatabase/archive/full/2015-02-01’ (select the most recent date stamp directory) and download ‘go_201502-termdb.obo-xml.gz’. Uncompress.

Copy all files and the ‘TIGRinfo’ folder into the ‘/TIGRfam_db’ folder.

(e) **Interpro**:

Go to 'ftp://ftp.ebi.ac.uk/pub/databases/Pfam/releases/Pfam27.0/database_files' (use appropriate version number) and download 'interpro.txt.gz'. Uncompress and copy the file into the '/Interpro_db' folder.

(f) **Rfam**:

Go to 'ftp://ftp.ebi.ac.uk/pub/databases/Rfam/CURRENT' for the latest release. At the time of writing, this is '11.0'. Download 'README', and 'Rfam.cm.gz' (when using Infernal earlier than version 1.1) or 'Rfam.cm.1_1.gz' (when using Infernal 1.1). Uncompress.

Go to 'ftp://ftp.ebi.ac.uk/pub/databases/Rfam/11.0/database_files' (use appropriate version number) and download 'rfam.txt.gz'. Uncompress.

Copy all files into the '/Rfam_db' folder.

(g) **rRNA database**:

Installed by default. This database is precompiled based on selected rRNA sequences and serves an a faster alternative to Rfam.

(h) **UniVec**:

The plasmid databases UniVec and UniVec_core are installed by default into the Blast database directory.

(7) *Final steps*

Before the first annotation run, please double check all parameters, in particular the chosen databases. Quite often you may want to use a different database (or database location) than the defaut one.

After the options for the new run have been chosen, make a habit of saving the configuration via the 'Save current selection' button.

3 Running a GAMOLA2 annotation

Call up GAMOLA2 with ‘perl Gamola_xxx.pl’ where xxx denotes the current version number. Click ‘OK’ on the welcome screen.
Make sure to call the correct ActivePerl version, otherwise some requirements or dependencies may be unresolved.

You may have to specify the path to the ActivePer distribution (e.g. ‘/opt/ActivePerl/bin/perl Gamola_2.00.12.pl’).

3.1 Design concept of GAMOLA2 GUI

The main window of GAMOLA2 is organised in two rows of tabs. The conceptual workflow consists of the user walking along the tabs from left to right until all options are set and then simply hits the ‘run’ button to start the annotation. The upper row denotes the main options, whereas the second row of tabs provides access to additional features. To set up an annotation run, follow the main tabs (upper row) from left to right.

Under the tab “Miscellaneous Options” a range of support features is offered that extend functionality beyond the initial automated annotation.

3.2 Startup options

3.2.1 System properties

This is the main starting screen which provides general setup options that allow you to maximise the speed of the annotation based on available computing resources.

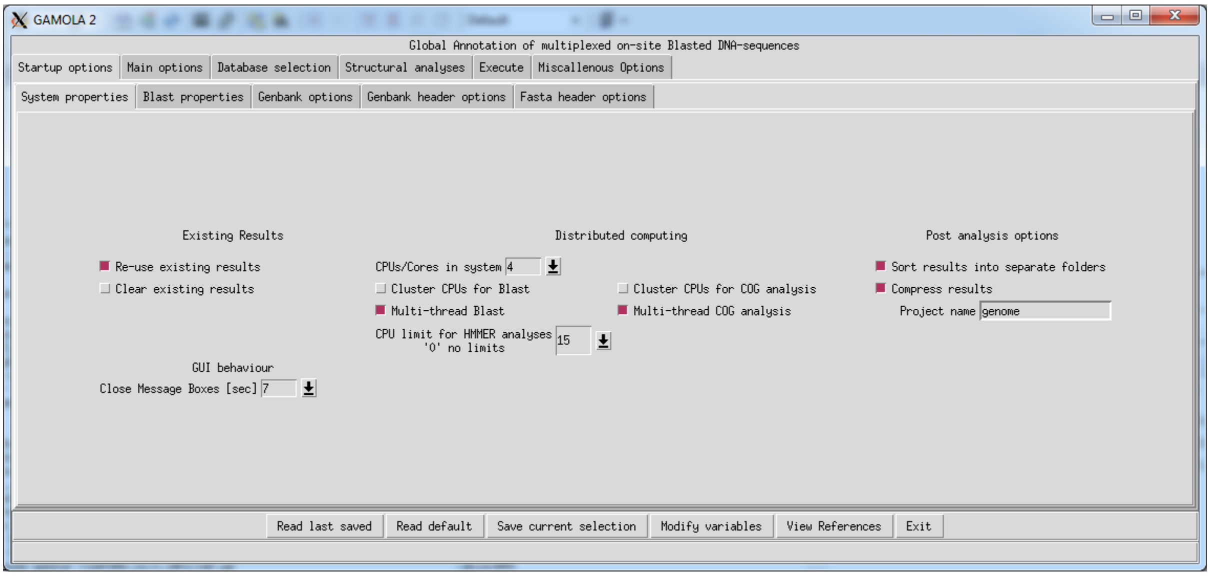

Existing results:

When starting a new run, it is best to make sure no old data files from a previous run are present in any of the directories. In this case, select "Clear existing results".

In other cases, such as resuming an interrupted run or re-running the same input sequence with different threshold levels, it is beneficial to keep existing results. GAMOLA2 will then test for each selected analysis which data files are already present and remove them from the pipeline (they are still used for annotation). Select "Re-use existing results" to recycle previous data sets.

Distributed computing:

Modern computers and server (farms) comprise multiple CPUs and/or cores. GAMOLA2 is highly multi-threaded and can be configured to utilise available resources.

"<u>CPUs/Cores in system</u>": select how many processes to run in parallel.

"<u>Cluster CPUs for Blast(COG) / Multi-threaded Blast(COG)</u>": Clustering CPUs will allocate all threads into one process (e.g. Blast and COG) while multi-threading initiates parallel instances. Default setting is "Multi threaded".

"<u>CPU limit for HMMER analyses</u>": Some versions of HMMER will automatically detect available CPUs/cores and launch a multi-threaded analysis. Limiting the number of parallel instances of HMMER will prevent an over-allocation of resources and a potential computer lockup. Select '0' to use the same CPU number as for Blast.

Post Analysis Options:

"Sort results into separate folders": GAMOLA2 saves results for all input sequences into the 'Results' folder, using the ORF numbers as provided by the final gene model. This option will consolidate the gene model (i.e. make ORF numbers sequential) and copies results for each input sequence into its separate folder within the 'Consolidated_results' folder. This option is selected by default.

"Compress results": Finished GAMOLA2 annotation runs often consist of thousands of individual data files. This option compresses both the original data files and the 'Consolidated data' – if selected – into ZIP archives, reducing the overall size of the data package and creating a convenient medium to transfer the complete annotation run from one location to another. The original result files are archived in the 'name'.object_results.zip archive. These data can also be used to populate the results folder and re-run an existing analysis with different thresholds. The consolidated data files are archived in the 'name'consolidated_results.zip archive. Both archive types can be used to work in Artemis. Enter a custom name for the archive into the entry field; default name is 'genome'. This option is selected by default.

GUI behaviour:

Select the number of seconds until information and error messages disappear automatically. Most errors are captured in error logs during the run and only critical error messages will permanently halt an analysis run.

3.2.2 Blast Properties

Options for Blast run parameters, Blast result files and the GAMOLA2 annotation.

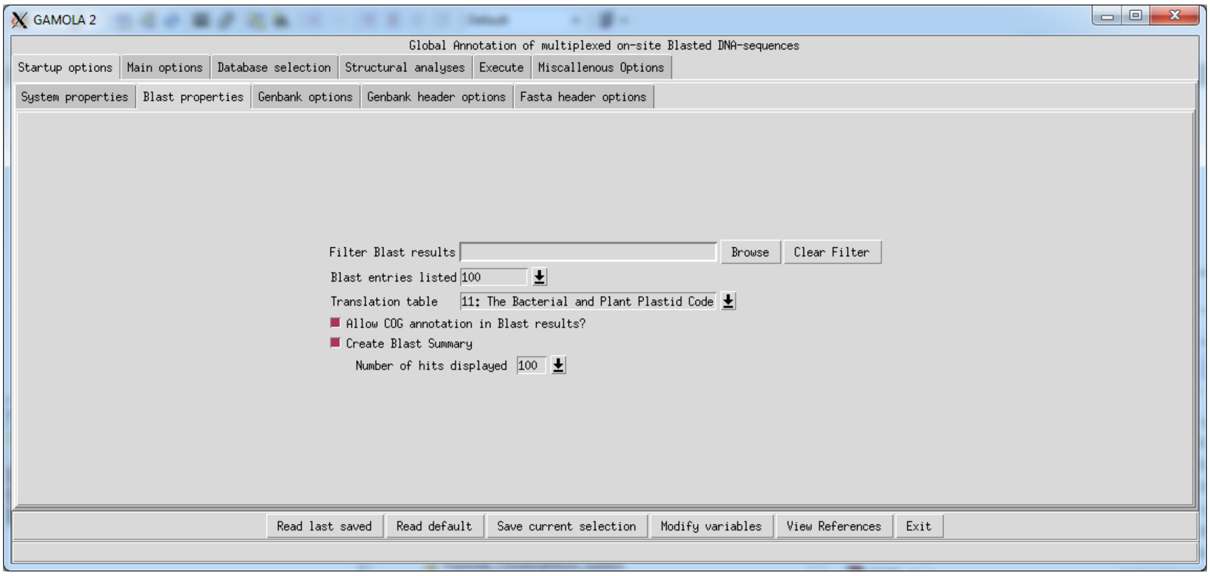

"Filter Blast results": The GAMOLA2 annotation lists the best Blast result in the annotation. To exclude unwanted entries (such as a self-hit or groups or organisms etc.) in the resulting Genbank file, a filter can be applied by typing in key words. For example, the phrase "Lactobacillus" will prevent a Blast hit carrying the "Lactobacillus" organism tag from being considered for the GAMOLA2 annotation. GAMOLA2 will traverse along the Blast hits until one is found that passes through the filter phrases. Should the best blast hit e-value be below the selected Blast threshold, the annotation will be shifted to 'hypothetical protein'. Can no Blast hit be found, the annotation will be 'unknown'. These annotations may later be changed by other selected analyses.

Multiple keywords can be chosen and are entered as a comma separated string. More complex filters or re-occuring phrases can be saved as an ASCII text file and imported using the 'Browse' button.

Remove any filter phrase by clicking on the 'Clear Filter' button.

"Blast entries listed": This option specifies the number of Blast hits shown in the Summary section in each Blast results file.

"Translation table": Select the most appropriate genetic code for the Blast analysis using the pull-down menu. GAMOLA2 is primarily designed for microbial genomes, but other biological origins may be more appropriate for specific annotation runs. This information will be added to the final Genbank file.

"Allow COG annotation in Blast results": On occasion Blast results can consist of straight entries from the COG database. To exclude these results from being listed as in the GAMOLA2 annotation, select this option.

"Create Blast Summary": If selected, this will create a Blast summary file, listing the 'N' best Blast hits for each ORF. This output can then be imported into Excel or similar software for quick data mining.

3.2.3 Genbank options

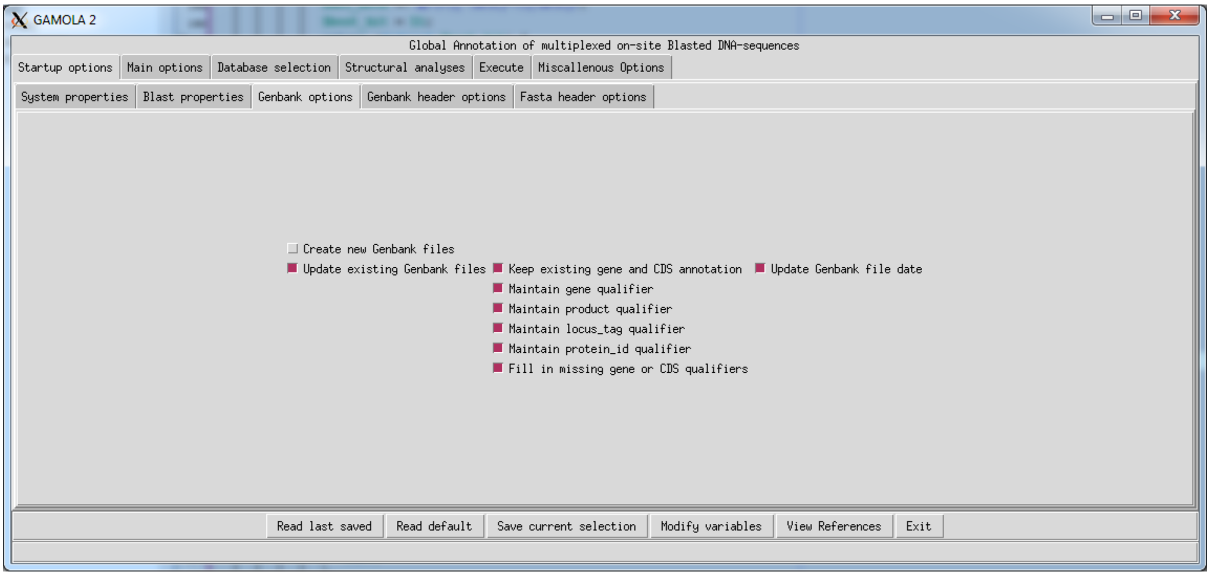

The main output of GAMOLA2 is an annotated Genbank file, created from either a FASTA or Genbank input file(s). If the input file is in FASTA format (or is a concatenated file), a new Genbank file will be created in all cases. If the input file is in Genbank format, the output file can either be a new Genbank file (select option '*Create new Genbank files'*) or an updated version of the input Genbank file:

'*Update existing Genbank files*':

(a) '<u>Keep existing gene and CDS annotation</u>': Check this option if an existing (manual) curation should be retained. Select further options ('*Maintain gene/product/locus_tag/protein_id qualifier*') to maintain individual qualifiers in a feature.

'*Fill in missing gene or CDS qualifiers*': In rare cases 'gene' or 'CDS' features may be missing the 'gene' qualifier. Select this option to add this qualifier to 'gene' and 'CDS' features.

(b) '<u>Update Genbank file date</u>': Select this option to update the Genbank file time stamp.

3.2.4 Genbank header options

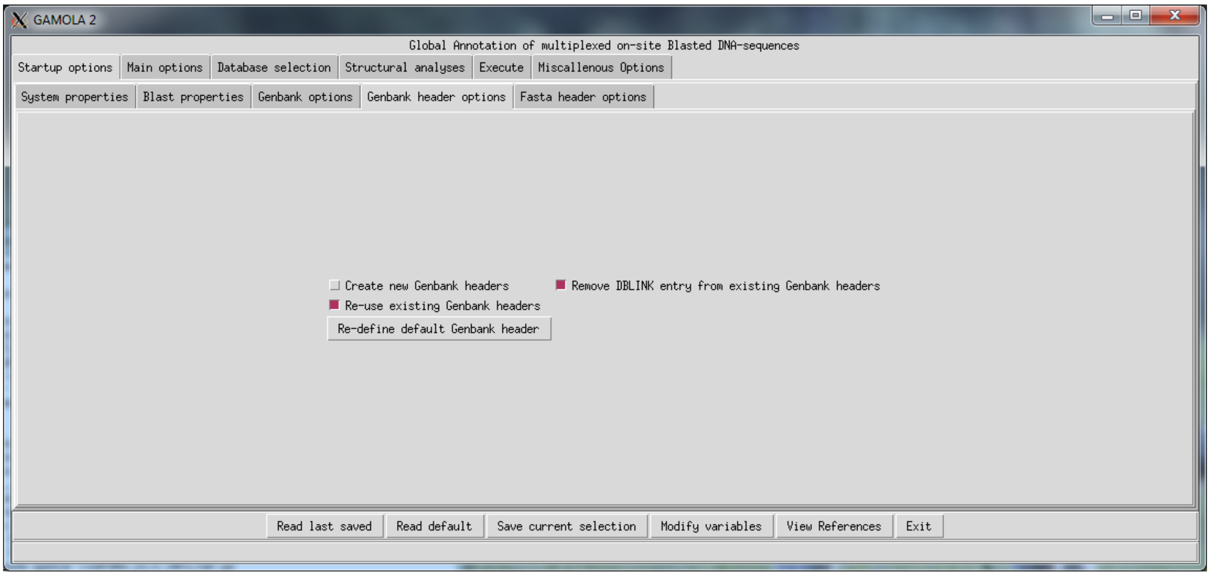

Re-use or create a new Genbank header for GAMOLA2 output files.

If the input file is in Genbank format, the existing Genbank head can be re-used for the new output file. Select '*Re-use existing Genbank headers*'.

If the input file is in FASTA format, or a new Genbank header should be created for the output file, select '*Create new Genbank headers*'.

A default Genbank header is created by default using the following format:

```
LOCUS        filename   bp    DNA   linear  BCT       16-FEB-2015
DEFINITION   filename
ACCESSION    unnamed
VERSION      1.0
KEYWORDS     GAMOLA annotation
SOURCE       none
  ORGANISM   none
REFERENCE    none
  AUTHORS    none
  TITLE      none
  JOURNAL    none
  PUBMED     none
COMMENT      none
FEATURES              Location/Qualifiers
     source
```

This header can be rebuild by clicking on the '<u>Re-define default Genbank header</u>' button

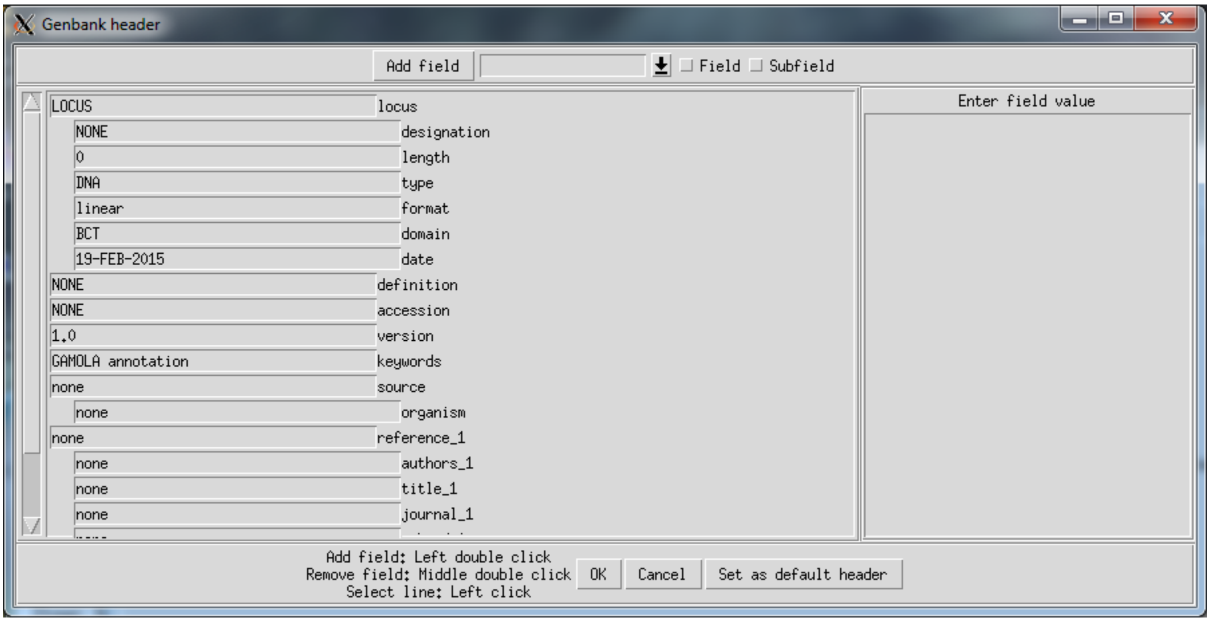

Select a new field from the pull down menu, select '*Field*' or '*Subfield*' and type in the default text in the '*Enter field value*'. Select a position in the existing Genbank header (left click), and the click '*Add field*'. The new entry will be added <u>below</u> the selected position.

Once finished, click '*OK*' for single use or '*Save as default header*' to be used in the future.

'<u>Remove DBLINK entry from existing Genbank headers</u>': Some versions of Artemis are incompatible with this header entry. Select this option to remove the entry from an existing Genbank input file and make the output Genbank file Artemis compatible.

3.2.5 *Fasta header options*

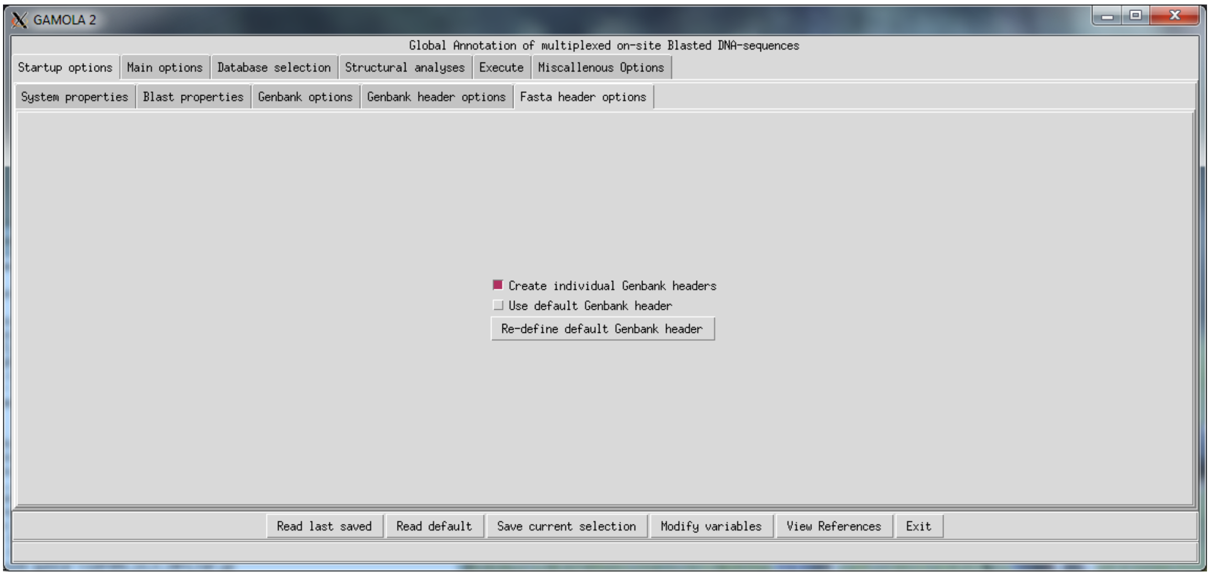

Choose whether FASTA input files will each have an individual header or use a common default header.

'Create individual Genbank headers': For each input FASTA file a Genbank header will be build (see above).

'Use default Genbank header': Each input FASTA file will be subjected to the default Genbank header structure. This option is selected by default.

'Re-define default Genbank header': See previous chapter for details.

3.3 Main options

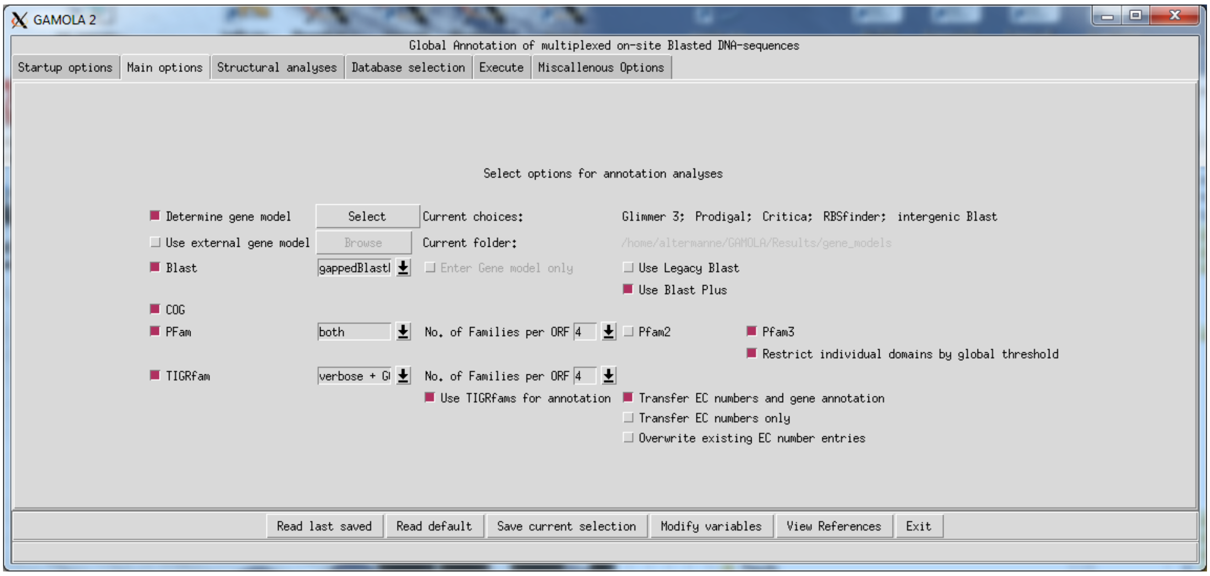

Here, the basic aspects of the annotation run are defined: Gene models, Blast, COG, Pfam and TIGRfam analysis.

3.3.1 Determine gene model:

Standard option for FASTA input files. All FASTA files will undergo a gene model prediction. Genbank input files can either fall back on the incorporated gene model or use ORF callers to create a new gene model (see Page 18). Exceptions include multi entry Genbank files or FASTA-Genbank file concatenations. In such cases Genbank files will be converted to FASTA, concatenated if required, and a new gene model be predicted. Further, if the Genbank file has no gene or CDS features, a new gene model will be built automatically.

Click ‘Select’ to define how the gene model will be predicted. Active choices are highlighted in black.

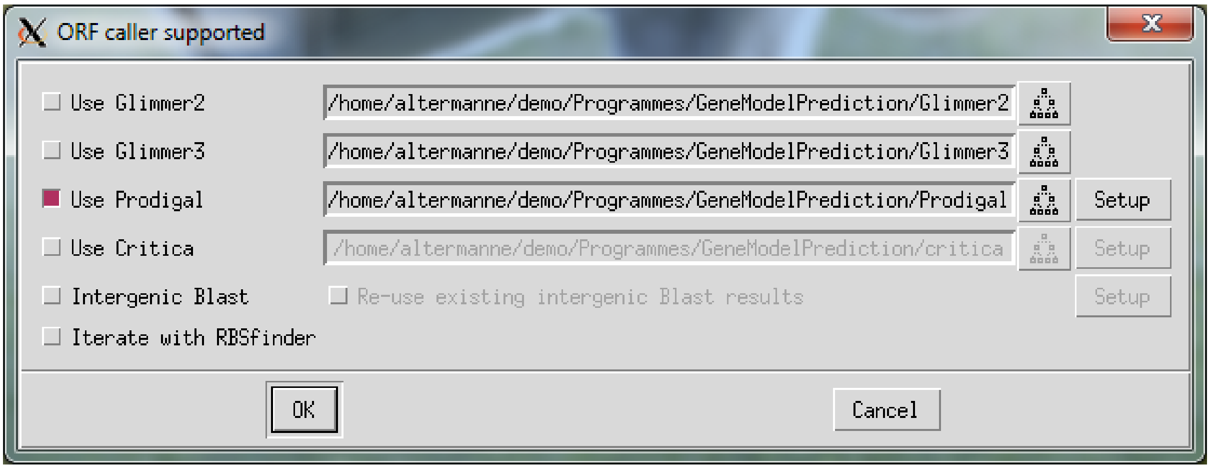

GAMOLA2 currently supports four different gene prediction tools (Glimmer2, Glimmer3, Prodigal, and Critica) plus a custom Intergenic Blast option. Finally, RBS sites can be predicted using a modified version of the RBSfinder package (15) and predicted gene start positions will be adjusted according to predicted ribosome binding sites. Either Glimmer2 or Glimmer3 can be selected.

Prodigal and Critica can be further customised by clicking on the 'Setup' button. Refer to the respective manuals for details on options.

Glimmer, Prodigal and Critica can be selected in parallel. GAMOLA2 then creates an additive gene model based on the results of the respective individual gene callers. The philosophy for choosing an additive model is that false positives can easily be deleted during the manual curation, but false negatives usually remain undiscovered unless they are very obvious.

'**Intergenic Blast'**: Gene models, in particular for fragmented early draft genomes, may omit ORF fragments (e.g. from frameshifts or due to contig boundaries). Select this option to identify partial ORFs that exhibit homology to known ORFs present in a Blast database and add them to the existing gene model. Intergenic Blast carries out a BlastP analysis. Intergenic ORFs are considered to be the maximum distance between two stop codons (or end of contig) on the same frame.

Click on 're-use existing intergenic Blast results' to use data from a previous run with the same input sequence(s).

Click on 'Setup' to customise the intergenic Blast:

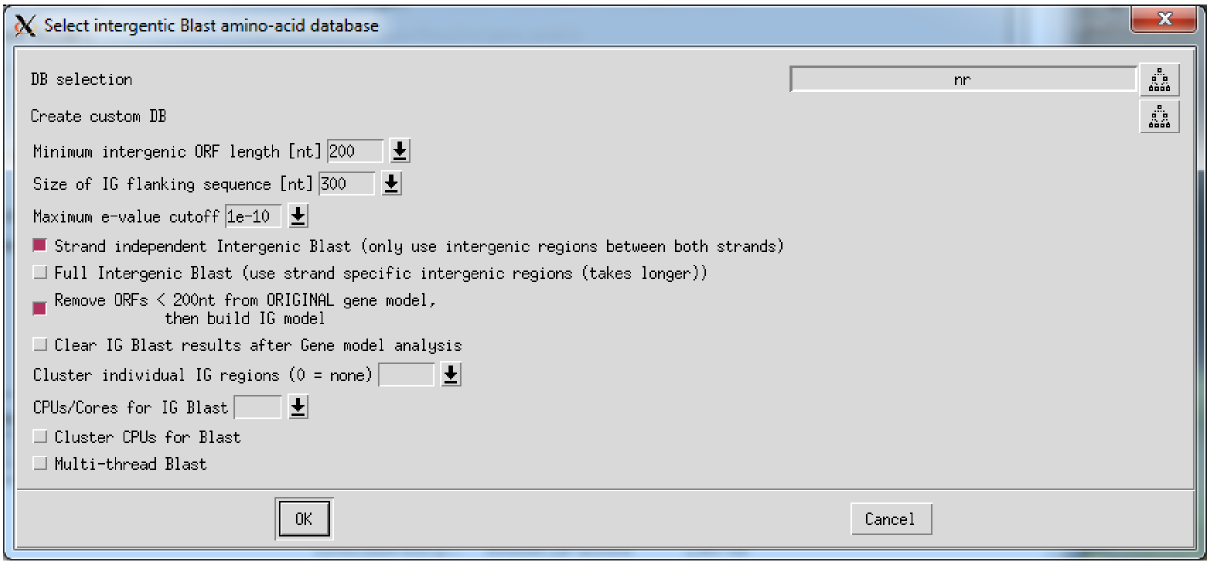

'<u>DB selection</u>': click on the file tree icon to browse and select the Blast database to be used for the intergenic Blast analysis. The default non-redundant Blast database will give the most comprehensive results, but will also require the most runtime. Custom BLAST databases can be created in GAMOLA2 and used for more targeted searches (e.g. use closely related genomes to identify frameshifts or incomplete ORFs).

'<u>Create custom DB</u>': click on the tree icon to create a custom amino acid Blast database.

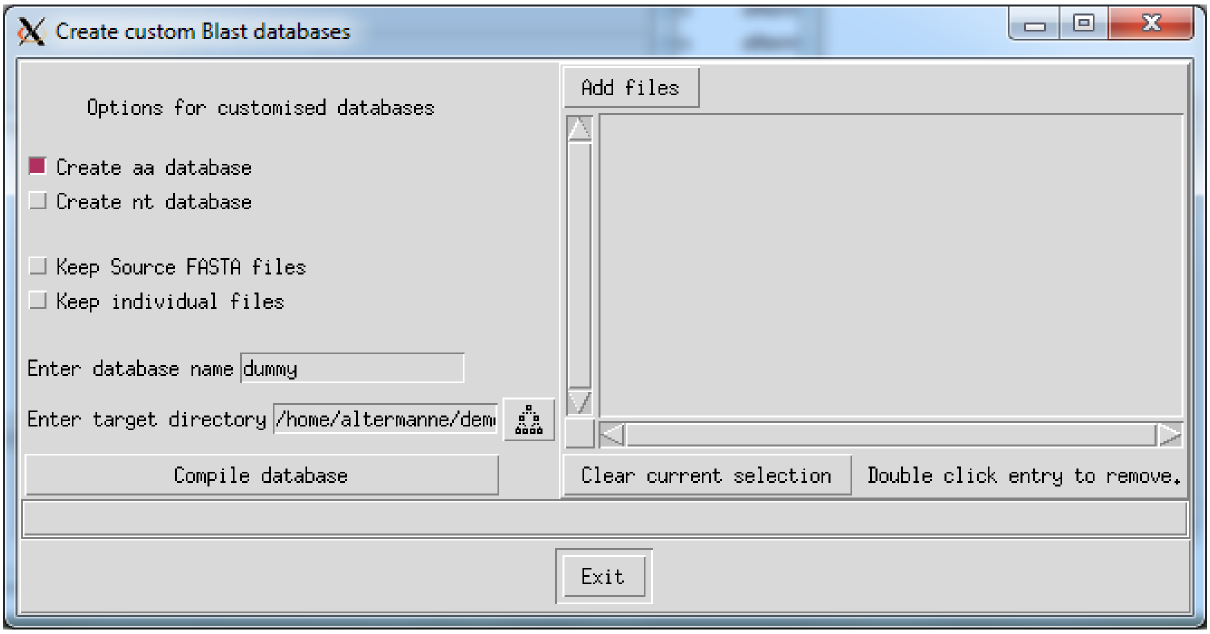

'Minimum intergenic ORF lenth [nt]': This sets the minimum length of an intergenic region.

‘Size of IG flanking sequence [nt]’: This sets the extend to which intergenic ORFs may overlap with adjacent ORFs determined by the additive gene model.

‘Maximum e-value cutoff’: This sets the upper threshold for Blast e-values. Only e-values below the threshold will be considered in the intergenic analysis.

‘Strand independent Intergenic Blast’: The gene model will be collapsed (remove ORF orientation). Intergenic regions are calculated I between non-coding regions between both sense and anti-sense ORFs.
or
‘Full Intergenic Blast’: This retains ORF orientation information. Intergenic regions are calculated between ORFs of the same orientation. This results in more intergenic regions to be analysed.

‘Remove ORFs < 200nt from ORIGINAL gene model, then build IG model’: In rare instances, gene callers specify very small ORFs that are likely to represent artefacts. This options removes all ORFs <200nt from the original additive gene model before determining intergenic regions.

‘Clear IG Blast results after Gene model analysis’: This option deletes the Blast results files from the intergenic analysis. De-active this option if results are likely to be re-used or the option ‘Re-use existing intergenic Blast results’ is selected.

‘Cluster individual IG regions’: This option submits ‘N’ intergenic regions to one Blast instance. While this will reduce runtime significantly, it may also lower sensitivity in some instances. Default value is ‘0’, no sequences are clustered.

‘CPUs/Cores for IG Blast’: sets the number of threads carried out in parallel. By default this is set to the system value. As a safety precaution, the value is reset to the system value each time the setup is invoked.

‘Cluster CPUs for Blast’ or ‘Multi-thread Blast’: choose whether resources are allocated to a single Blast instance or if one Blast instance per CPU/core is invoked.

‘**Iterate with RBSfinder**’: Select to predict potential ribosomal binding sites. This feature is currently considered experimental. Please report any errors or anomalies.

3.3.2 Use external gene model

Gene models created previously for the same input file can be re-used using this options. Alternatively, external gene model can be imported for a given input sequence. Supported formats are GFF and the internal GAMOLA2 format. File name conventions are: input_file_name.gff and input_file_name.combined.

Gene model files are comprised of space delimited lines with four columns: ORF_number, start position, stop poisiton, orientation:

1 1315 1196 [-1
2 1534 1355 [-1
3 1700 1975 [+1

Similarly, the expected format for GFF is a tab delineated line: ORF, Left boundary, right boundary, orientation ('+' or '-').
Gene model files must be placed in the gene model folder '/Results/gene_modes'.

3.3.3 Blast

Blast is still considered to be the basic workhorse of annotation. Check this tickbox to include a Blast analysis. If deselected, only the gene model will be entered into the output to serve as template for all other analyses.

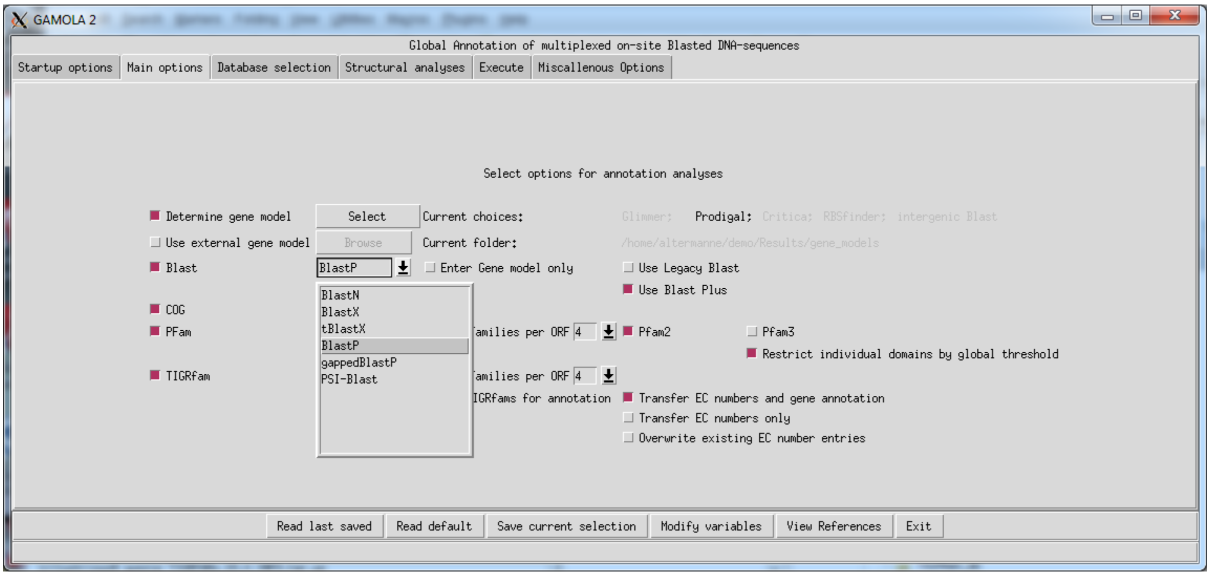

Supported Blast flavours include BlastN, BlastX, tBlastX, BlastP, gappedBlastP and PSI-Blast. Both Legacy Blast and Blast plus packages are supported. Default is Blast plus.

3.3.4 COG

Select the COG checkbutton to include a COG analysis.

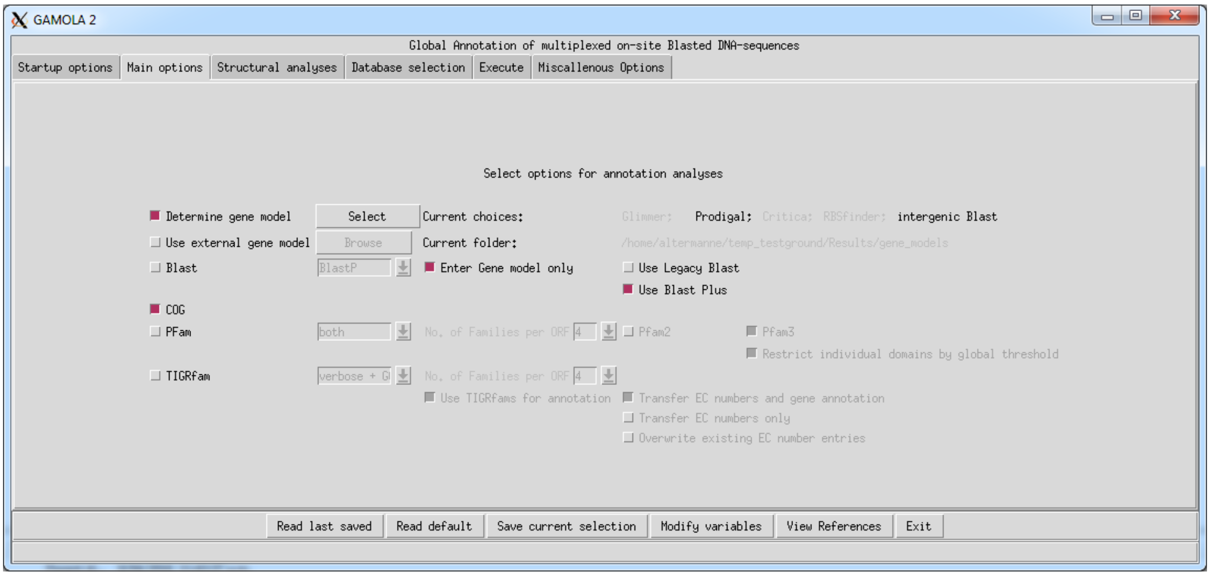

3.3.5 *Pfam*

Select the Pfam checkbutton to include a Pfam analysis. Both Pfam2 and Pfam3 are supported.

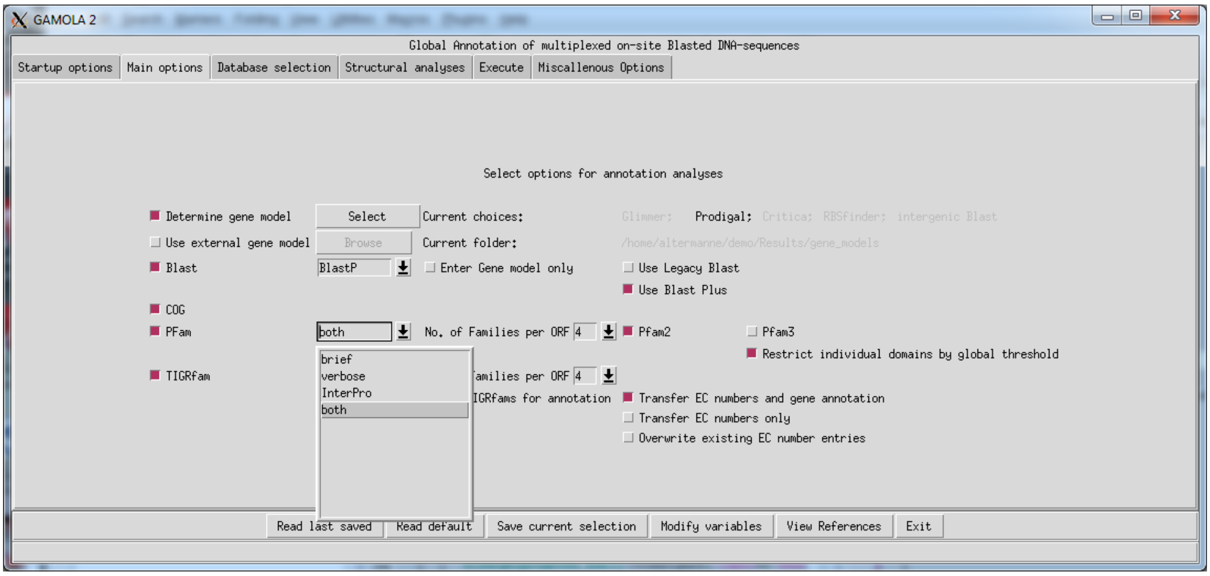

Choose the verbosity of the annotation:

'brief': only a minimal annotation is provided for Pfam domains.

'verbose': The full Pfam descriptor is added to the annotation.

'InterPro': The InterPro descriptor for the Pfam domain is added to the annotation.

'both': Both Pfam and InterPro descriptors are added to the annotation.

'No. of Families per ORF': This option limits the number of Pfam families listed per ORF. Families are chosen by decreasing significance.

'Restrict individual domains by global threshold': Takes into account only the full sequence e-value of HMMER3. Individual domains with weaker e-values are still shown.

3.3.6 *TIGRfam*

Select the TIGRfam checkbutton to include a TIGRfam analysis.

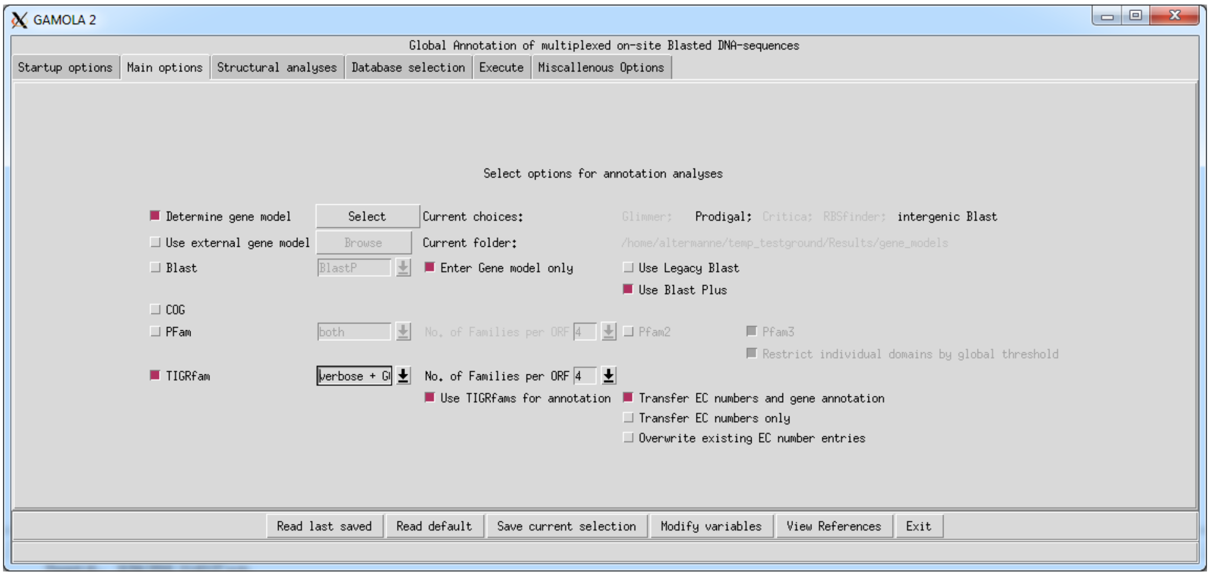

Choose the verbosity of the annotation:

'brief': only a minimal annotation is provided for TIGRfam domains.

'verbose + GO': The full TIGRfam descriptor is added to the annotation plus Gene Ontology information where available.

'No. of Families per ORF': This option limits the number of TIGRfam families listed per ORF. Families are chosen by decreasing significance.

'Use TIGRfams for annotation': Often TIGRfams contain well curated domain descriptors and gene names. Selecting this option overwrites previous Blast annotation in favour of the most significant TIGRfam hit and its respective annotation. Where possible, gene names are utilised in the Genbank gene features. This option is selected by default.

'Transfer EC numbers and gene annotation': Where available, EC numbers are added as a qualifier to CDS features and gene annotations be used in gene qualifiers for gene and CDS features.

'Transfer EC number only': Only EC numbers are added to the annotation as qualifier in the CDS feature.

'Overwrite existing EC number entries': This option will overwrite existing EC number qualifiers with the TIGRfam EC number.

3.4 Structural Analyses

Structural analyses provide additional information for protein function and for non-coding structures. This information adds an additional layer of information to the functional annotation, helping to deduce or narrow down the biological function of a predicted ORF.

3.4.1 tRNA/rRNA/ncRNA

3.4.1.1 tRNA:

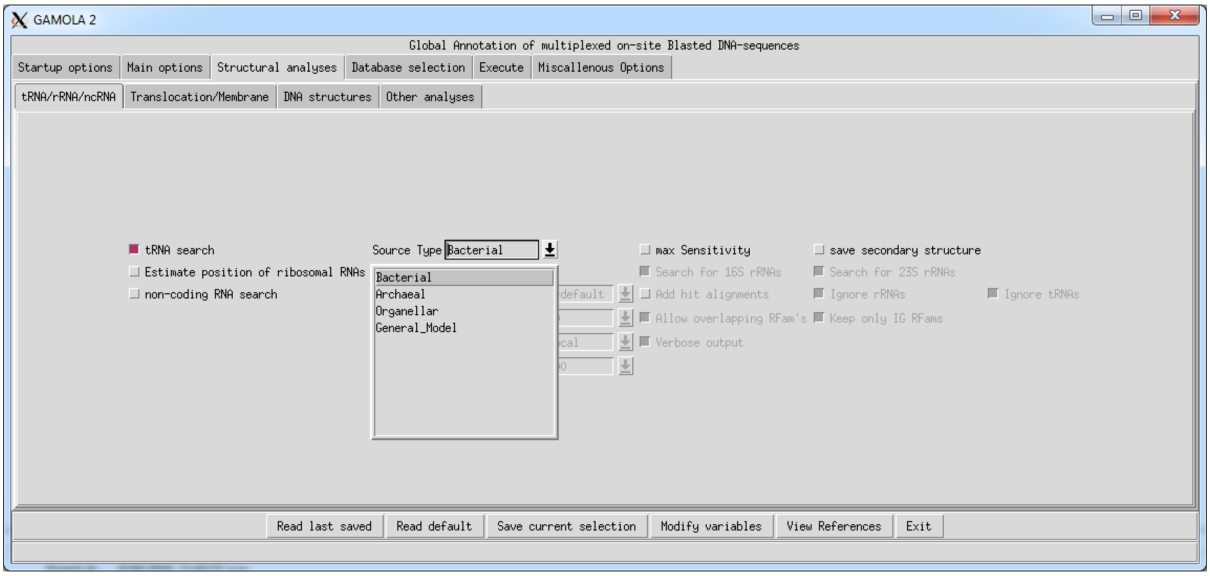

Transfer ribonucleic acid (tRNA) is a type of RNA molecule that helps decode a messenger RNA (mRNA) sequence into a protein. A microbial genome typically features between 30 and 50 tRNAs. Specific settings for bacterial and archaeal genomes, for sequences of organellar origin and a general model can be selected from the “Source Type” pull down menu. Optional settings are: ‘max Sensitivity’ and ‘save secondary structure’. ‘max Sensitivity’ increase the rate of detection with a time penalty cost. ‘save secondary structure’ will create an additional output file in the tRNAscan results folder “xxx.structure” that will show the predicted secondary structure of tRNAs.

3.4.1.2 Estimate position of ribosomal RNAs

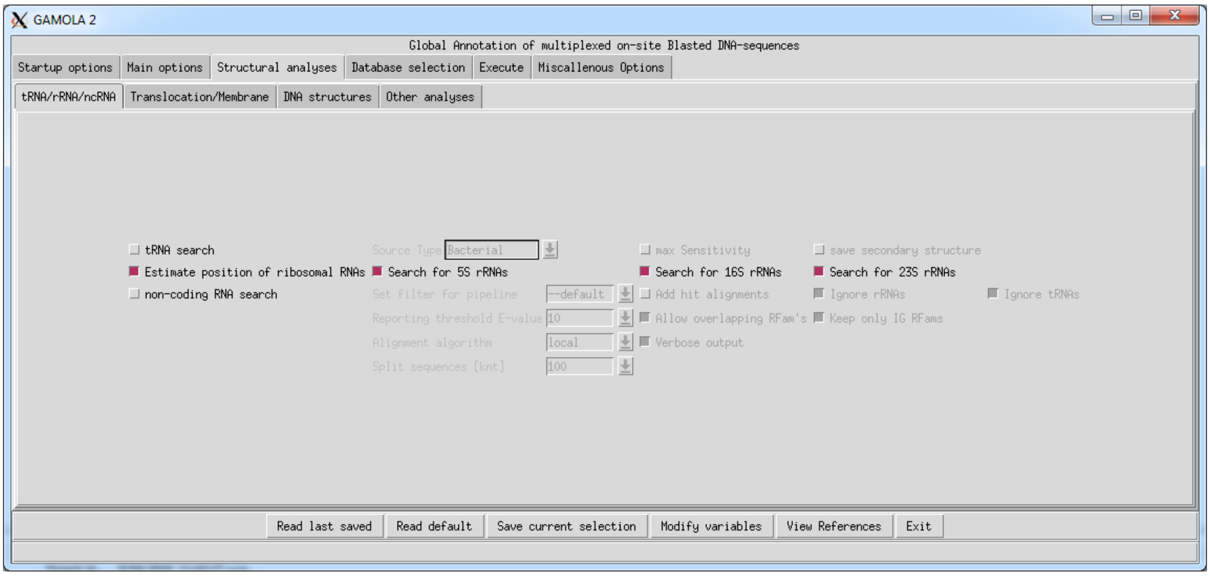

This option aims to predict the rough positions of 5S, 16S and 23S rRNAs based on a custom Blast database harbouring respective microbial rRNA sequences. Individual rRNA positions are calculated based on their respective Blast alignments and are indicative only. This feature is particularly helpful when assembling draft genomes, as it will reveal partial rRNA genes at contig boundaries.

3.4.1.3 Non-coding RNA search

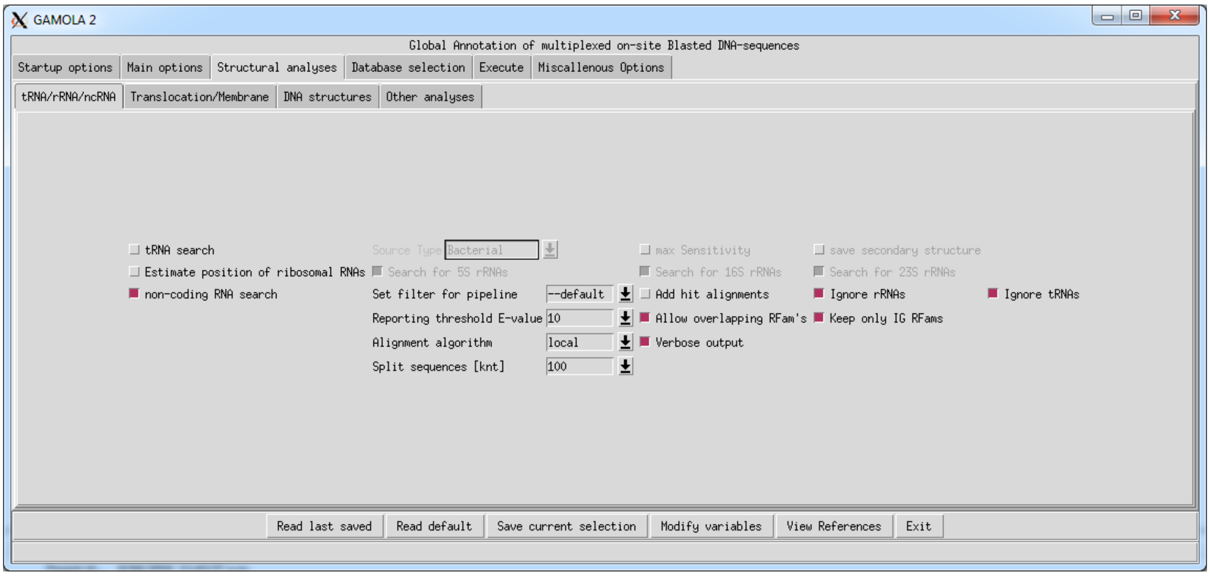

A non-coding RNA (ncRNA) is an RNA molecule that is not translated into a protein. Non-coding RNA genes include highly abundant and functionally important RNAs such as transfer RNAs (tRNAs) and ribosomal RNAs (rRNAs), as well as RNAs such as snoRNAs, microRNAs, siRNAs, snRNAs, exRNAs, piRNAs and scaRNAs and the long ncRNAs that include examples such as Xist and HOTAIR (see here for a more complete list of ncRNAs) [grabbed from Wikipedia].

Several parameters can be set for ncRNA search (short descriptors grabbed from Infernal manual, for more details refer to full manual):

- Set filter for pipeline: There are five options for controlling the general filtering level. These options are, in order from least strict (slowest but most sensitive) to most strict (fastest but least sensitive): -max, -nohmm, -mid, -default, -rfam and -hmmonly.
- Reporting threshold e-value
- Alignment algorithm (local/glocal): The glocal alignment algorithm is global with respect to the query model and local with respect to the target database. By default, the local alignment algorithm is used which is local with respect to both the target sequence and the model.
- Split sequences [knt]: sequences that are too long may omit some hits as reporting thresholds are reached.
- Add hit alignments: This option adds respective sequence alignments to the output files
- Allow overlapping RFam's: overlapping ncRNA sequences will be shown in the output Genbank file. Deactivating this option will retain only the ncRNA with the highest score for a given group of overlapping subjects.

- Verbose output: Checking this option will provide a more verbose ncRNA descriptor in the output Genbank file.
- Ignore rRNAs/tRNAs: ncRNAs also include rRNA genes and tRNAs. Checking these two options will remove rRNA and tRNA hits from the result list and avoids duplication of these features in the output Genbank file if the separate tRNA/rRNA options from above were selected.
- Keep only IG RFams: ncRNAs are identified independent of a genemodel. Selecting this option will remove all ncRNA hits that would fall within a predicted coding sequence.

3.4.2 Protein Translocation/Membrane proteins

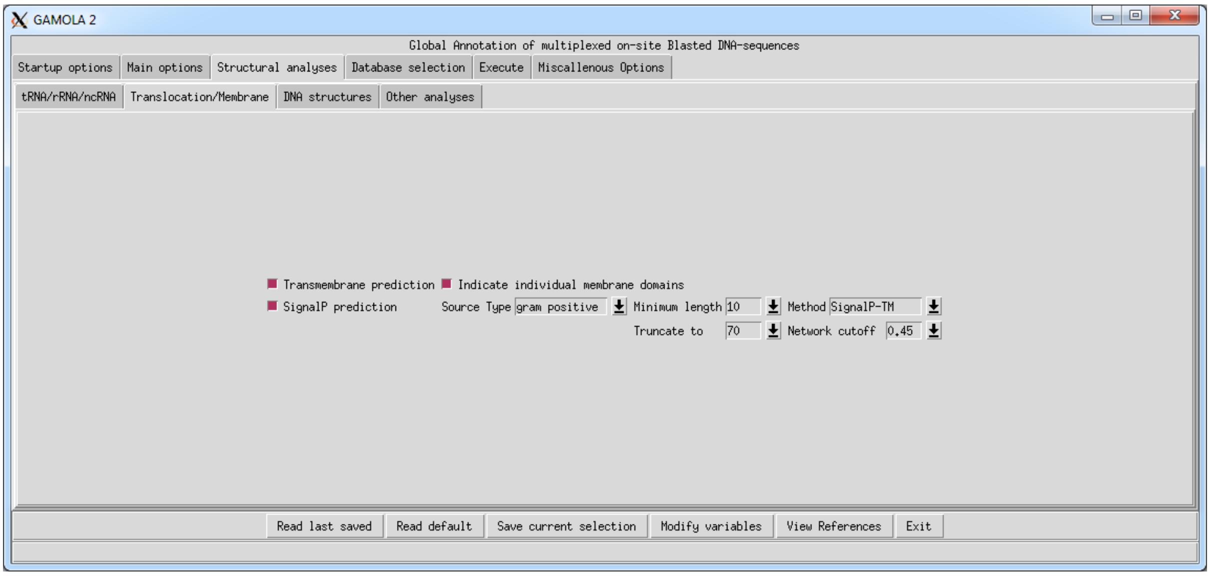

Note: Both TMHMM and SignalP are NOT part of the GAMOLA2 distribution package, due to the licensing system implemented by CBS. Please read instructions above to download both packages individually.

3.4.2.1.1 Transmembrane prediction:

Prediction of transmembrane helices in proteins. Selecting the ‘Indicate individual membrane proteins’ options will resolve individual TMH helices within a given ORF in the output Genbank file.

3.4.2.1.2 SignalP prediction

The prediction of presence and location of signal peptide cleavage sites in amino acid sequences. GAMOLA2 supports both SignalP v3.0 and v4.1b. On first installation of GAMOLA2, SignalP v4.1b is assumed (see figure above). Is SignalP v3.0 be found and compiled instead, then exit GAMOLA2 after the installation process has finished and restart to adjust the GUI to SignalP v3.0 parameter setup.

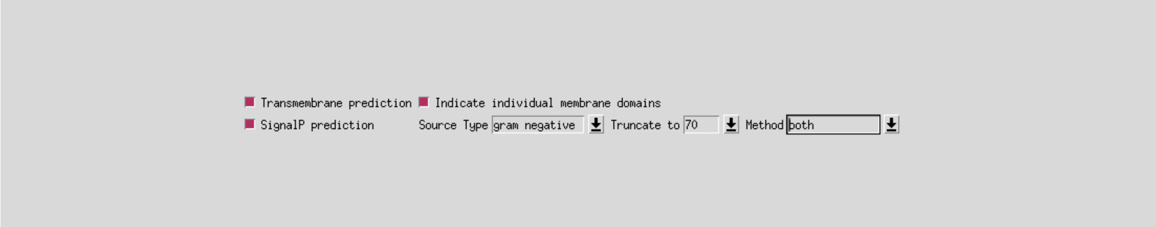

Alternative SignalP v3.0 GUI

Options for SignalP v4.1b are:

- Source Type: Search gram positive, gram negative or choose the eukaryotic model.
- Minimum length: Determines the minimum amino acid length for a signal peptide.
- Truncate: This option sets the maximum length from the N-terminus for the cleavage site position.
- Method: Set the algorithm to deliver best results using neural networks predictions from either SignalP-TM or SignalP-noTM networks. For gram positive bacteria, always chose 'SignalP-TM'.
- Network cutoff: Defines user specified D-cutoffs for TM and noTM networks. Values are automatically set to default values for any given organism-network combination, but may be adjusted manually in the entry field. Default values are for recommended SignalP v4.1b cutoffs. To simulate SignalP v3.0 values, adjust the respective cutoffs according to the table below.

Suggested D-value cutoffs to simulate SignalP v3.0

D-cutoffs for TM and noTM networks	
Euk	0.34
Gram -	0.42
Gram +	0.42

Options for SignalP v3.0 are:

- Source Type: Search gram positive, gram negative or both microbial cell types or choose the eukaryotic model.
- Truncate: This option sets the maximum length from the N-terminus for the cleavage site position.
- Method: Limit the algorithm to use either neural networks or hidden Markov models. Default option is to use both types for maximum sensitivity.

3.4.3 DNA structures

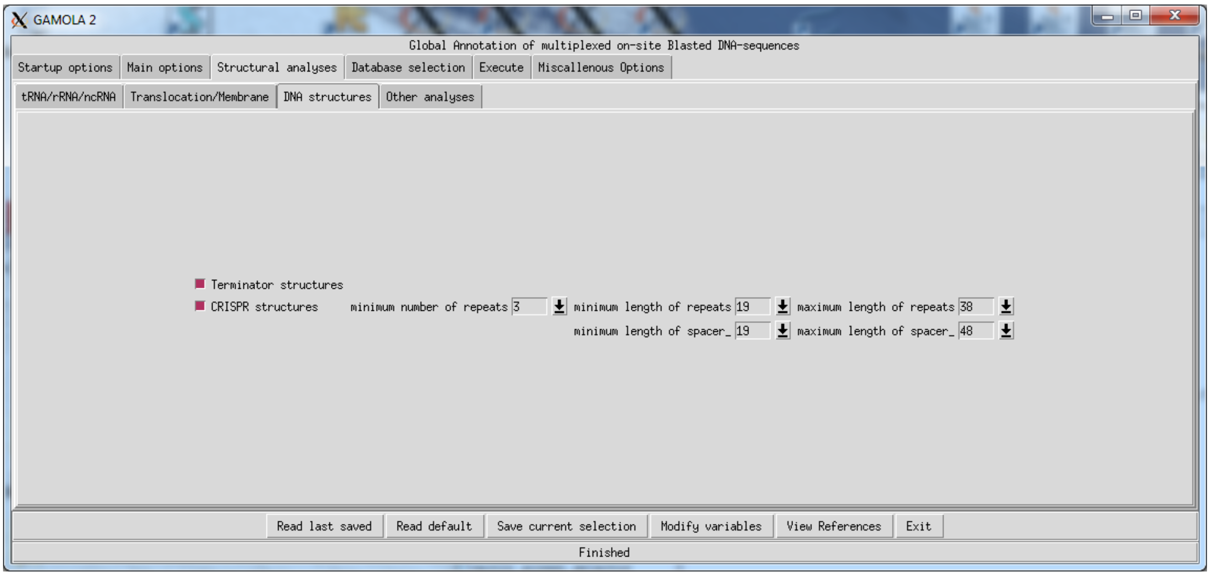

3.4.3.1.1 Terminator structures:

Rho-independent terminator structures stop the RNA transcription in prokaryotes by forming a stem-loop structure, usually between 7 to 20 basepairs in length. Checking this option will investigate the query sequences for the presence of rho-independent terminator structures and add them to the output Genbank file.

3.4.3.1.2 CRISPR structures

Clustered regularly-interspaced short palindromic repeats (CRISPR) are segments of prokaryotic DNA containing short repetitions of base sequences. A number of options are available to fine-tune the detection limits:

- Minimum number of repeats
- Minimum/maximum length of a single repeat
- Minimum/maximum length of a single spacer

Values can be changed from the dropdown menu or by manually entering values.

3.4.4 *Other analyses*

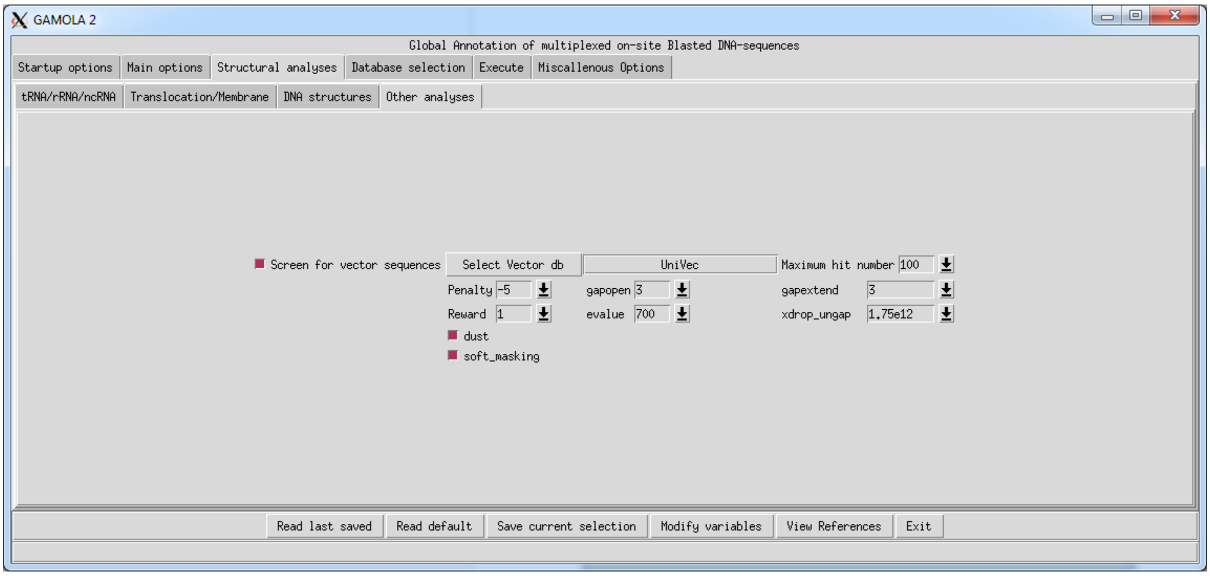

Presently, this section offers the option to screen query DNA sequences for the presence of known vectors or vector fragments, which can occasionally contaminate draft genome sequences if the read filtering was flawed. Two relevant NCBI vector databases are installed by default: UniVec and UniVec_Core. These two databases can be found in the Blast database directory. To increase the hitrate of vector sequences, concatenated sequences using the default GAMOLA spacer will be broken up into their respective individual contigs. Vector hits are classed as "weak", "moderate" or "strong", depending on their respective scores. BlastN hits with multiple alignment regions are treated as a single joint feature. The maximum length for such a joint feature is 10,000nt. Joint hits exceeding this length will be trimmed to the first alignment. Vector hits are sorted according to their overall score and alignment length. Hit quality is calculated based on the sum of all section alignment scores.

The UniVec database contains the most comprehensive list of vector, linker, adapter, and primer sequences. However, this also increases the risk of false positives and manual curation is strongly recommended. UniVec_Core represents a selected subset of UniVec and, while not as comprehensive as UniVec, may result in less false positive hits.

Available options are:

- Maximum hit number: Number of UniVec or UniVec_core hits displayed across the entire query sequence.
- Penalty: Penalty for a nucleotide mismatch
- Reward: Reward for a nucleotide match
- Gapopen: Cost to open a gap
- Gapextend: Cost to extend a gap

- Xdrop_ungap: X dropoff value for ungapped extensions in bits
- Dust: Filter query sequence with dust
- Evalue: Expectation value (E)
- Soft_masking: Apply filtering locations as soft masks

Default values for BlastN were taken from the NCBI VecScreen system (http://www.ncbi.nlm.nih.gov/tools/vecscreen/about/) and can be adjusted via check boxes and pull down menus.

3.5 Database selection

GAMOLA2 supports a number of different generic and custom databases. Here, specific databases and model files can be selected for a given annotation run.

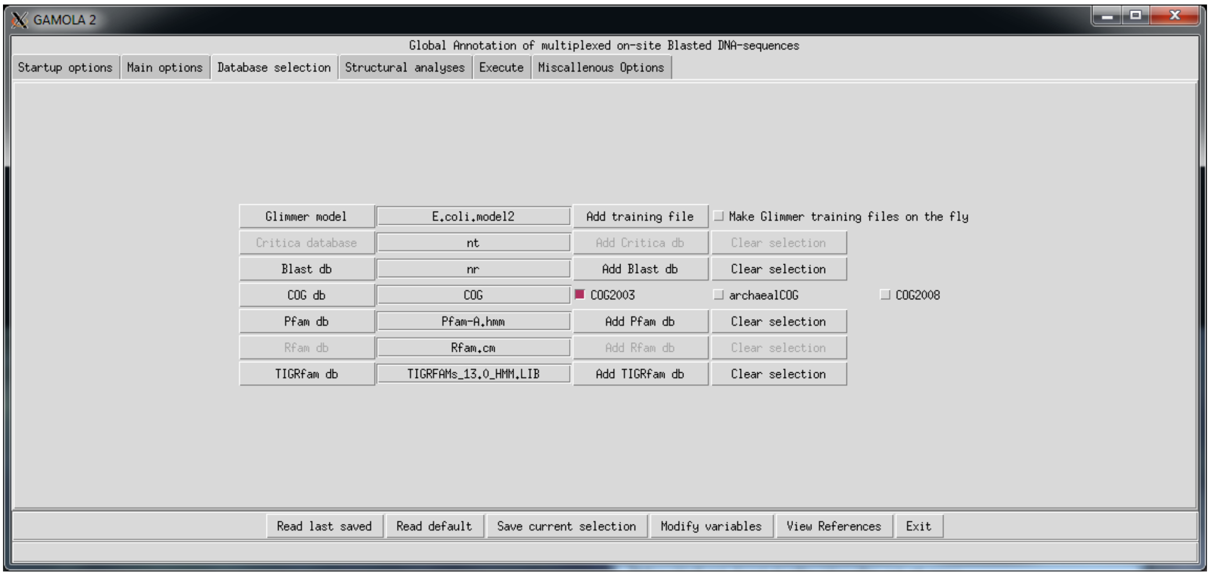

3.5.1 'Glimmer model':

Chose an existing and appropriate Glimmer2 or Glimmer3 model file by clicking on the 'Add training file' button. Alternatively, Glimmer can be self-trained on any given input sequence of sufficient length by ticking the check button 'Make Glimmer training files on the fly'.

3.5.2 'Critica Database':

The Critica gene caller requires a nucleotide database. By default, the NCBI 'nt' database is selected, however customised databases can be used to reduce runtime. To select a new Critica database, click on the 'Add Critica db' button. To clear the current selection, click on the "Clear selection" button.

3.5.3 'Blast Database'

Blast can accept a number of different amino acid or nucleotide databases, depending on the chosen Blast flavour. Blast databases can comprise both default databases such as the NIH non-redundant nr and aa databases, as well as purpose build Blast databases. Only one Blast database

per run is allowed. If more than one is selected, only the first one will be used. To select a new Blast database, click on the ‘Add Blast db’ button. To clear the current selection, click on the “Clear selection” button.

3.5.4 ‘COG Database’

Currently, six different COG databases are supported by GAMOLA2 which can be selected from the drop-down menu. These databases are installed by default and must be located in the expected directory.

3.5.5 ‘Pfam Database’

Both Pfam2 and Pfam3 databases can be chosen, dependent on the Glimmer version chosen before. Multiple Pfam databases can be selected for a single run. If more than one database is selected, then GAMOLA2 will analyse each database in the order selected until at least one significant hit below the choses e-value threshold is found or it runs out of databases to test. To select a new Pfam database(s), click on the ‘Add Pfam db’ button. To clear the current selection, click on the “Clear selection” button.

3.5.6 ‘Rfam database’

The Rfam database is installed by GAMOLA2 during installation. Other, updated versions can be chosen by clicking on the ‘Add Rfam db’ button. To clear the current selection, click on the “Clear selection” button.

3.5.7 ‘TIGRfam database’

Multiple TIGRfam databases can be selected for a single run. If more than one database is selected, then GAMOLA2 will analyse each database in the order selected until at least one significant hit below the choses e-value threshold is found or it runs out of databases to test. To select a new TIGRfam database(s), click on the ‘Add TIGRfam db’ button. To clear the current selection, click on the “Clear selection” button.

3.6 *Execute*

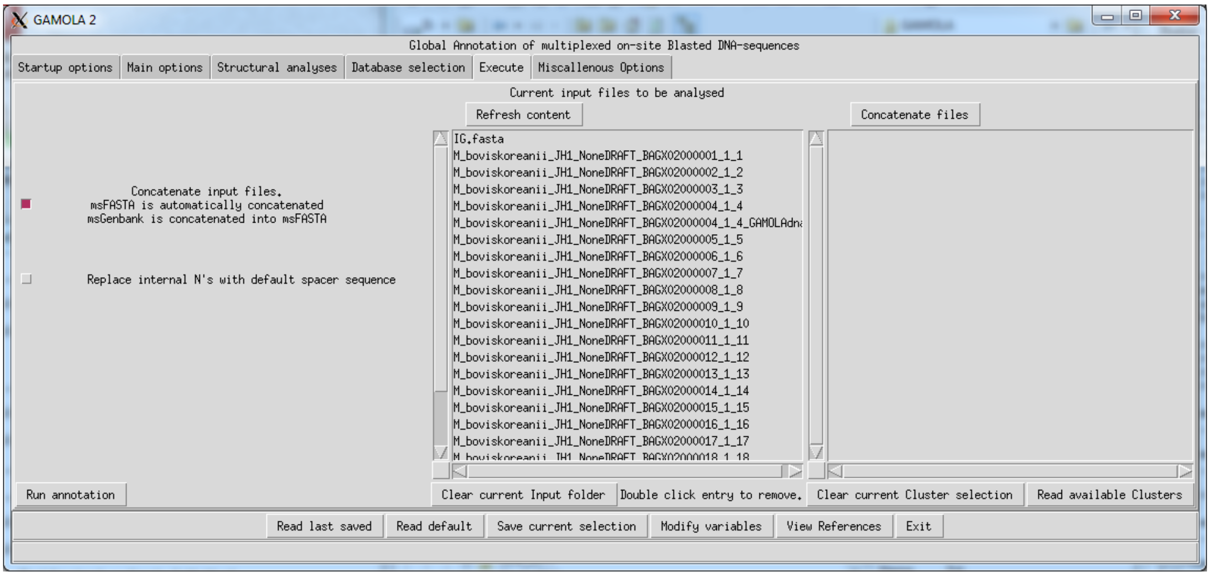

At this stage, all running parameters have been set up and it is advisable to save the setup for future re-use. To do so, click on the 'Save Current Selection' button in the bottom row, click on 'Save' and confirm to overwrite any existing setup file.

To change any e-value thresholds, click on the 'Modify variables' button in the bottom row and change e-values for Blast, COG, Pfam and TIGRfam. These thresholds determine whether a given hit is considered for annotation or not. For genomes with well characterised ORFs, lower e-values are more suited, whereas novel genomes benefit from relaxed settings (i.e. higher e-values) that allow distant hits to be shown in the annotation. Don't forget to 'Save' the changed parameters.

3.6.1 Input sequences

The middle frame shows all input files found in the './Input_sequences' folder. Accepted file formats are FASTA and Genbank. Both formats can be freely mixed and may contain multiple entries.

The folder content can be refreshed by clicking on the 'Refresh content' button above the list.

FASTA and multi-sequence FASTA (msFASTA) input files are expected to contain nucleotide sequences in the following format:

```
'>header1
ACTGTGACGATCGAAGTTTCGCGCCAAGCTAAG…
>header2
ACTGACTAGAGGCTTTAAACCCACGTCAGAC…
>…'
```

The sequence may only contain unambiguous letters (i.e. 'A, C, T, G') and 'N' and is not case-sensitive. All other characters will be converted to 'N'.

Genbank and multi-sequence Genbank (msGB) files must contain the nucleotide sequence and a header starting with 'LOCUS' and ending with 'FEATURES'. If a gene model is provided, both 'gene' and 'CDS' features are recognised.

GAMOLA2 attempts to anticipate and automatically correct for many common variations and errors in entry files.

3.6.2 Concatenate input files

GAMOLA2 offers two different ways of dealing with many input sequences (and msFASTA and msGB files).
The first option is to treat each individual entry as a separate entity and create individual annotated Genbank files. This option is best if multiple independent sequences are used within a single annotation run.
The second option is to concatenate entries into a single entity. This options is used most often when dealing with draft genomes where multiple contigs are present.

3.6.2.1 How to work with concatenation

Check the 'Concatenate input files' box on the left hand side. All input sequences will be concatenated using a non-bleeding spacer sequence. This spacer creates stop codons in all six reading frames, preventing the gene model to 'bleed' across contigs.
Some sequences contain individual or strings of 'N's – indicating known gaps in the assembly. Check the 'Replace internal 'N's with default spacer sequence' to replace any string of 'N' with the default non-bleeding spacer sequence. *Note: This will NOT be recognised as multiple contigs, but still treated as a single sequence.*

msFASTA and msGB files can be freely mixed, however, Genbank files will lose their respective gene models and a new one will be created for the resulting concatenated sequences. Each individual input file will result in a separate, annotated Genbank file, with individual entries in respective msFASTA and msGB files concatenated.

3.6.2.2 Concatenate specific groups of sequences

GAMOLA2 supports the creation of specific sequence sets to be concatenated from a pool of existing entries. This allows to carry out multiple annotations within a single run and provides flexibility in the way sequence data is analysed. Common scenarios for concatenation groups are annotating multiple draft genomes or combining chromosomes and plasmids into a single annotated sequence.
To define annotation groups, click on the 'Concatenate Files' button in the top right and a new window will appear.

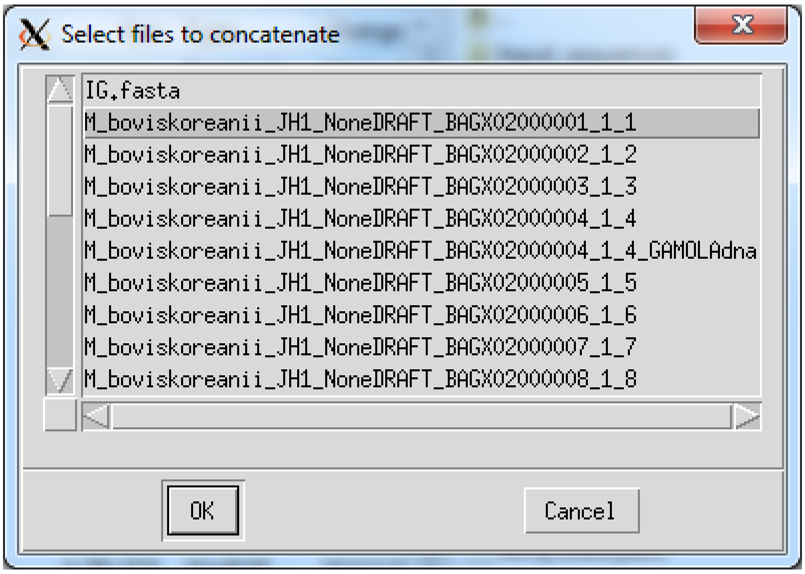

Select multiple entries, using the standard 'Shift' and 'CTRL' conventions. Selected entries are shaded.

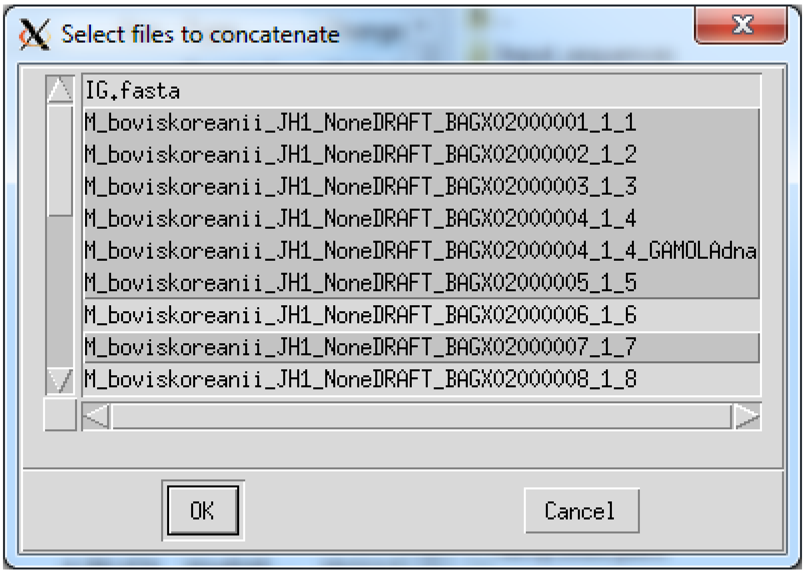

Hit the 'OK' button to create the first concatenation group.

Repeat this process until all groups have been assigned. Each concatenation group is shown as a color-coded block in the right-hand frame.

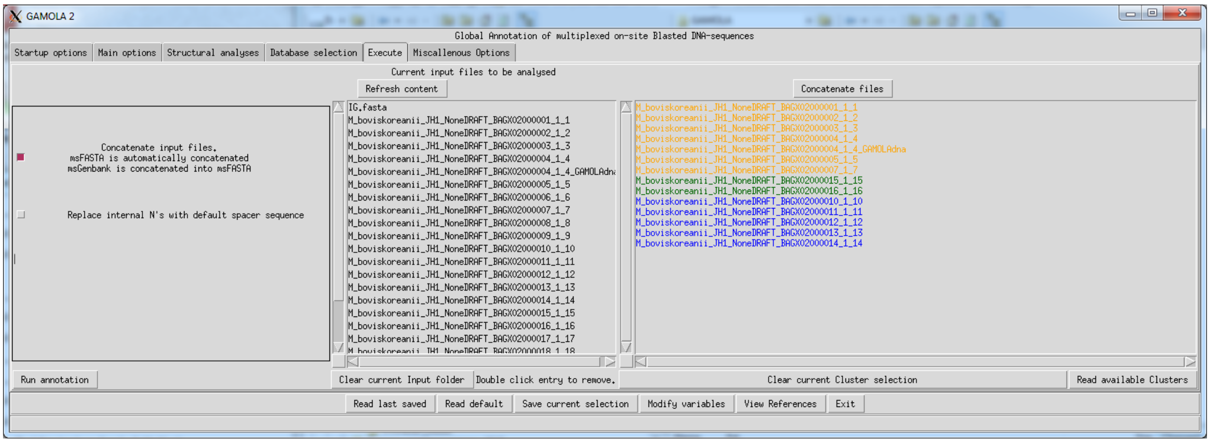

To remove the selection of concatenated sequence groups, click the 'Clear current Cluster selection' button.

3.6.2.3 Re-use of selected concatenation clusters

If a GAMOLA run fails or needs to be re-done, the previously made concatenation scheme can be re-loaded by clicking on the 'Read available Clusters' button. This option will be useful, when multiple and/or complex concatenation groups have been selected.

3.6.3 *Start the Annotation Run*

To start the annotation run, simply click on the ‘Run annotation’ button in the lower left corner.

At the start, several safety checks are being performed in the background to ensure the system has all resources required to run the annotation.

A final safety check requires user approval in case the ‘Clear existing results’ option was selected in the ‘Startup options’.

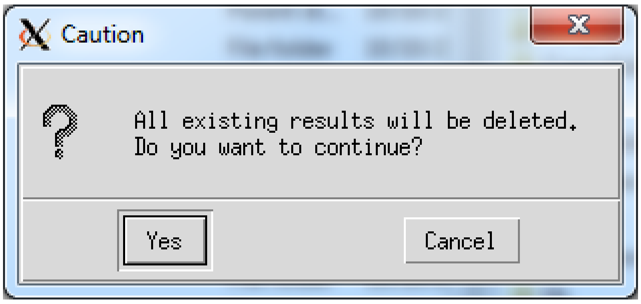

Double check that any existing results have been saved or can be safely deleted. Click on ‘Yes’ to continue with the analysis.

Depending on the number and sizes of input sequences and databases selected, an annotation run can last from a few minutes to several days.

In the case of a hardware failure, existing results can be re-used. They will be checked for corruption when a new GAMOLA run is initiated and faulty or incomplete results will be removed. The annotation run will then continue, eliminating the risk of having to start from scratch again.

4 GAMOLA2 output

The output of a GAMOLA annotation run is always a selection of text files that can be opened by most programs. There is no need for an internet connection or other resources, other than a genome browser (see below). This reflects the basic philosophy of GAMOLA to provide a highly flexibly and independent annotation system that can be used anywhere, anytime. This is especially useful when dealing with preliminary or confidential data that should not be transmitted to public services or where portability is important.

4.1 The Genbank file

The annotated Genbank file is the most important GAMOLA output. It contains the full annotation and much information on functional and structural features. The Genbank header can be customised prior to a run, otherwise a short default header is created. The Genbank file follows the common guidelines, but has a few extra feature keys added, depending on the analyses selected.

Briefly (for a more detailed guideline for annotation see below), each predicted ORF comprises both a 'gene' and a 'CDS' feature with the same start and stop positions. Respective annotations are coded within the 'gene' qualifier, where the 'gene' feature displays a short descriptor and the 'CDS' feature a verbose description, wherever possible. Each ORF is assigned a unique number, found at the very end of the 'gene' qualifier. This number must to be changed while working within the GAMOLA annotation system.

Most software packages will be able to open the GAMOLA Genbank file successfully.

4.2 Genbank associated files

There are two extra files provided in case of concatenated sequences.

(1) A file with the concatenated sequence in FASTA format. Concatenated sequences can be recognised by the '.cb' prefix (for 'contig boundaries') in the FASTA and Genbank files.
(2) A text file (.contig_order) that lists the original sequence file, contigs created and the respective start and stop position for each contig.

4.3 Genbank associated data directories

All underlying results for each ORF/sequence are saved as individual text files in their respective directories. A separate folder for Blast, COG, Pfam, TIGRfam, ncRNA, rRNA, tRNA, and terminators, CRISPRs, SignalPs and transmembrane helices as well as vector contaminations is created. For easy reference, each result file carries the unique ORF number found in the Genbank file.

4.4 Results sorted into individual directories

Initially, all results are saved in to their respective folders within the 'Results' directory, carrying the original gene model designations. When analysing multiple sequences in one run, this can easily become cumbersome due to the large number of files residing in the directories. Further, in some instances, ORF numbers may not be sequential due to changes during the gene model processing.

When the option 'Sort results into separate folders' is selected in the Startup options, then results for each input file (or concatenated cluster) will be copied to the 'Consolidated Results' directory and there into a separate folder, maintaining the original results folder structure. Further, the gene model will be rectified at this point, making all ORF designators sequential.

4.5 Archive files

Complete results for annotated sequence can easily comprise several thousand individual data files. Transfer of such a large number of individual small files can be cumbersome and time consuming. In the Startup menu, select 'Compress Results' and select an appropriate archive name.

When selected, results files will be copied into a ZIP archive:

- '.object_results.zip': contains the original result files as saved in the 'Results' folder. Use this archive to re-constitute completed runs that you want to re-run.
- '.consolidated_results.zip': if the 'Sort results into separate folders' option was selected, a second archive file is created, that contains results sorted by input sequences into separate folders. Use this archive file for further analyses and manual annotation curation.

5 Recommended Genome Browser

Artemis (14) is one of the best and fastest offline microbial genome browsers around and is still under development by the Sanger Institute (http://www.sanger.ac.uk/science/tools/artemis). Artemis is a pure Java application and can therefore be run on any Java-supported operating system. The GAMOLA annotation system has been built around the Artemis architecture with the aim to provide an easy-to-access system that is tailored to the needs and requirements of microbiologists.

We have modified the Artemis genome browser to better suit the additional features implemented by GAMOLA, in particular to correctly display functional and structural features and to retrieve the underlying results for a given ORF for Blast, COG, PFam and TIGRfam results. The current Artemis build is version 16.

The current GAMOLA distribution provides the modified Artemis genome browser.

5.1 Installation of Artemis

Artemis is a standalone JAVA application and the only requirement is the presence of a Kava distribution. Most systems have Java installed by default, however a common distribution can be found here: http://java.com/en/download/windows_xpi.jsp (make sure to disable any bloatware during the installation process).

Once Java is installed, copy the ‘Artemis.v16.GAMOLA’ folder from the distribution archive to a folder of your choice. Due to some memory limitations of the Java engine, it is highly recommended to increase the allocated memory space.

5.1.1 *Quick Guide to increase Java memory allocation (for Windows machines):*

For other operating system, the same principles apply, individual steps may vary.

- Create a shortcut to the Artemis.jar file on your desktop.
- Right-click on the icon and select ‘Properties’
- In the ‘Target’ line, modify the existing string to:
  "C:\Program Files\Java\jre7\bin\java.exe" -Xmx1000M –jar C:\Artemis\Artemis.jar
- Adjust the respective paths to your Java and Artemis file locations.
- Start Artemis by double-clicking on the shortcut icon.

5.2 Working with Artemis and a GAMOLA annotated sequence

5.2.1 Color codes for recognised GAMOLA2 features:

GAMOLA2 works with a wide range of different features to deliver a comprehensive overview of gene and non-coding features. Each feature comes with its own color code to provide an optical reference guide when analysing genomes in Artemis. Color codes used in GAMOLA2 are shown in Table 1.

Table 1: Features and their associated color codes

Feature	Color code
**RBS**	Grey
**Gene**	White
**CDS**	Cyan
**COG_match**	Red
**PFam_match**	Green
**TIGR_match**	Blue
**tRNA**	Light Green
**rRNA**	Gold
**ncRNA**	Yellow
**SignalP**	Dark Grey
**TMM**	Light Grey
**Terminator**	Magenta
**CRISPR**	Light Blue
**Vector_match**	Light Red
**Fasta_record (contigs)**	Orange and brown, alternating

5.2.2 *Feature Overview*

(1) When starting up Artemis, you will be prompted to set the working directory. Select the path to your GAMOLA annotated Genbank file. If you work on this file on a regular basis, make sure to tick 'Save between sessions' before clicking on 'OK'.

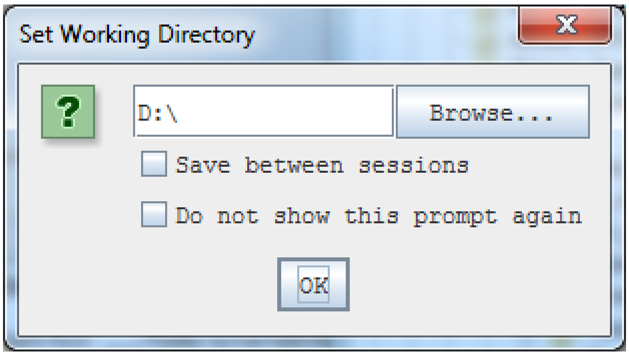

(2) Select the GAMOLA Genbank file with 'File->Open'

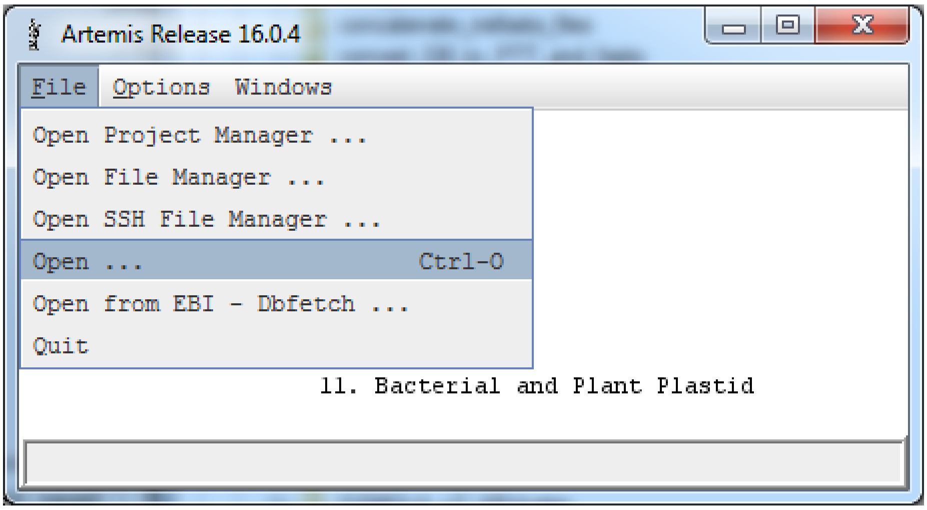

(3) The Genbank file will load up and the annotation window will pop up

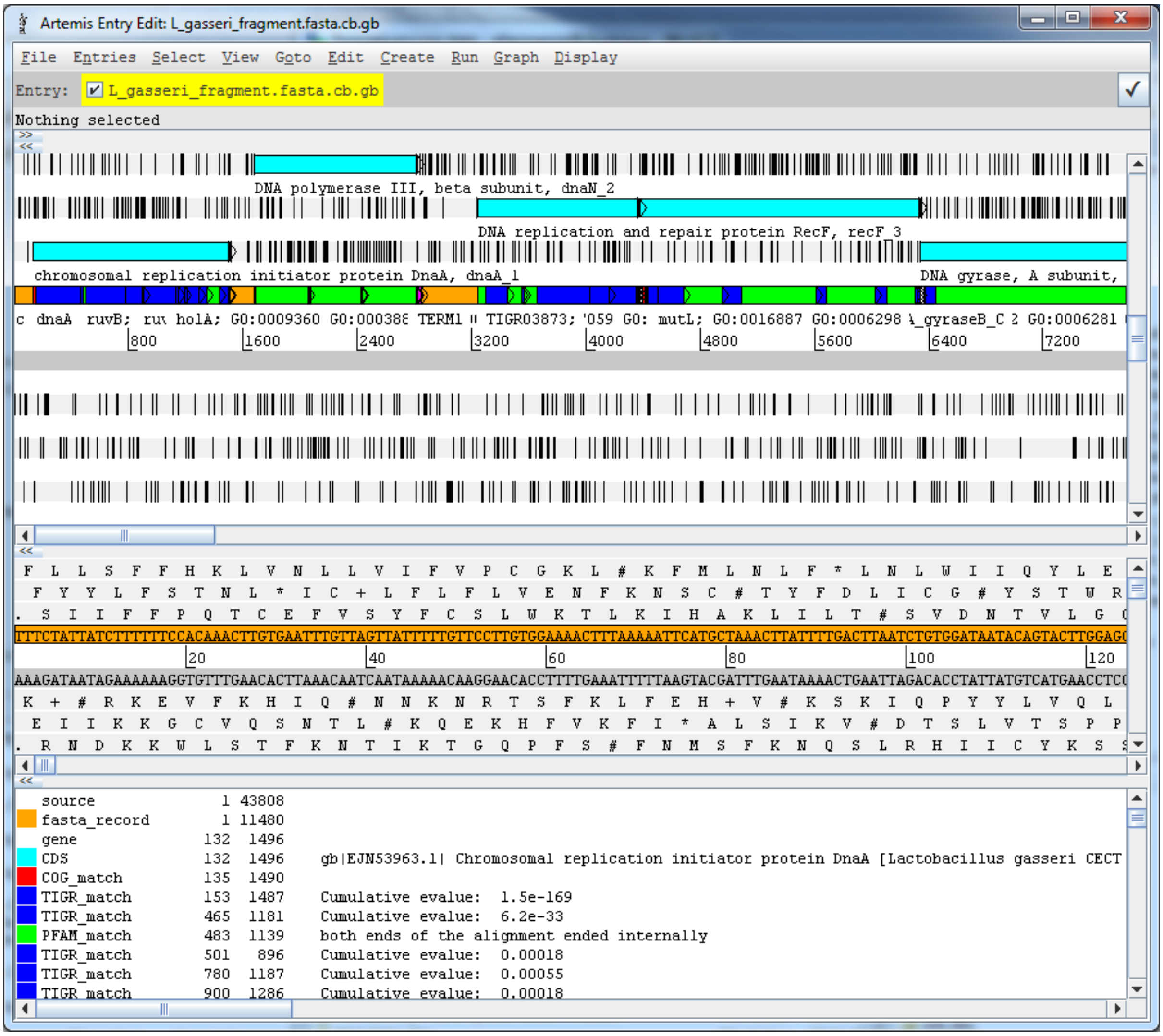

Three panels are visible:

On the top, the overview (sense, antisense strand and all six frame translations) with features and stop codons is shown.

In the middle, a zoomed view to the nucleotide level is provided

In the lower panel, a description of all features is listed.

THINGS TO DO:

The first thing you want to do is right click into the lower panel and select ‘Show products’. This will change the display to show much more useful information, such as annotation and respective Lengths, Scores and e-values.

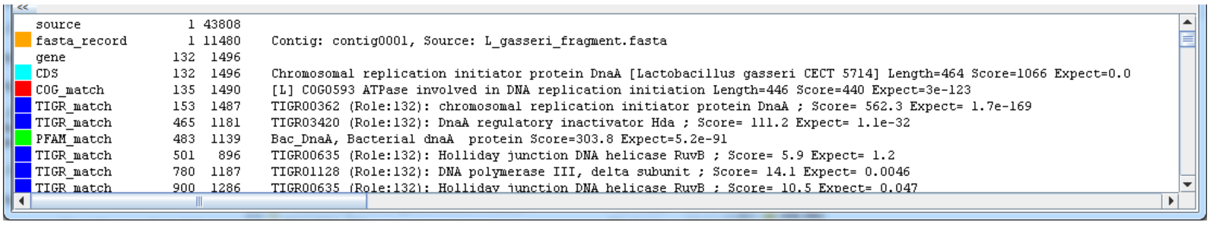

(4) How does it work? The makeup of an ORF

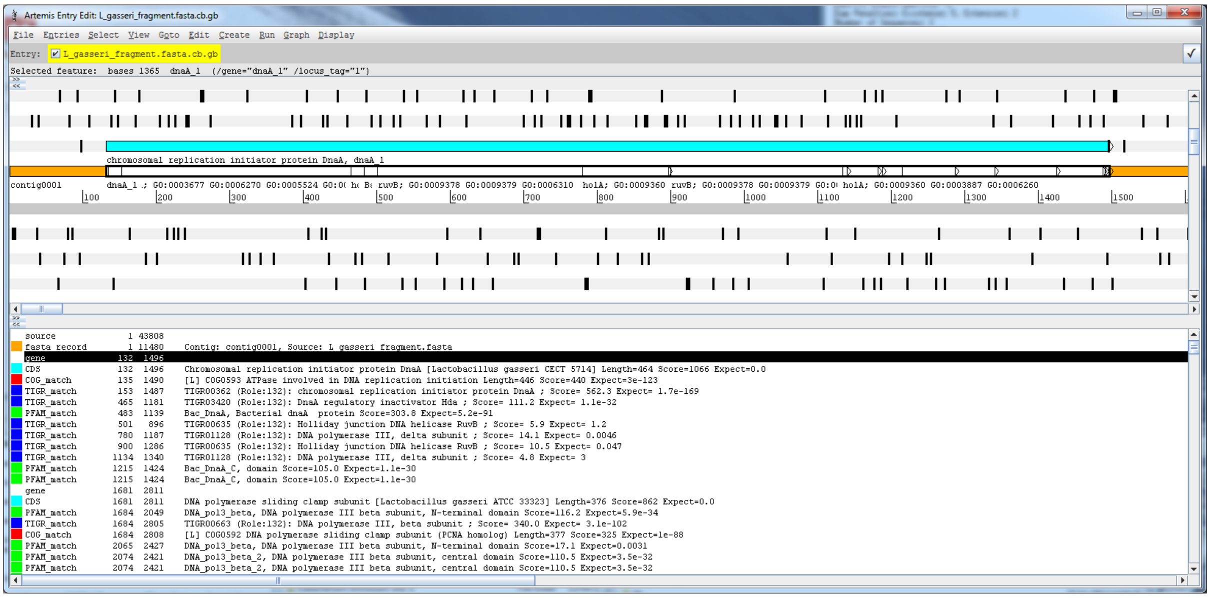

The picture above shows a zoomed in version of an ORF (for more detailed information on the Artemis GUI, please refer to the Artemis manual).

(a) Each predicted ORF consists of a ‘gene’ (white arrow-box on either the sense or antisense strand) and a ‘CDS’ feature (cyan arrow-box located in one of the six reading frames), comprising the same start and stop position

(b) all functional or structural hits that related directly to this ORFs, are found within the respective ORF boundaries. In the lower panel, the white ‘gene’ and the cyan ‘CDS’ feature always indicate a new ORF, respective associated hits (e.g. COG in red, TIGRfam in blue, Pfam in green) are shown below. A new ‘gene’ and ‘CDS’ feature pair always indicates a new ORF.

(5) Information accessible for individual features:

Information is available directly through the features. Select any feature and open up a new window via <CTRL+E> shortcut.

(a) **'contig' feature**: Contigs are shown as 'fasta_records' in alternating brown and orange colors. They serve as a visual reminder of contig boundaries and the non-bleeding spacer sequence between two contigs prevents ORF-bleeding between contigs.

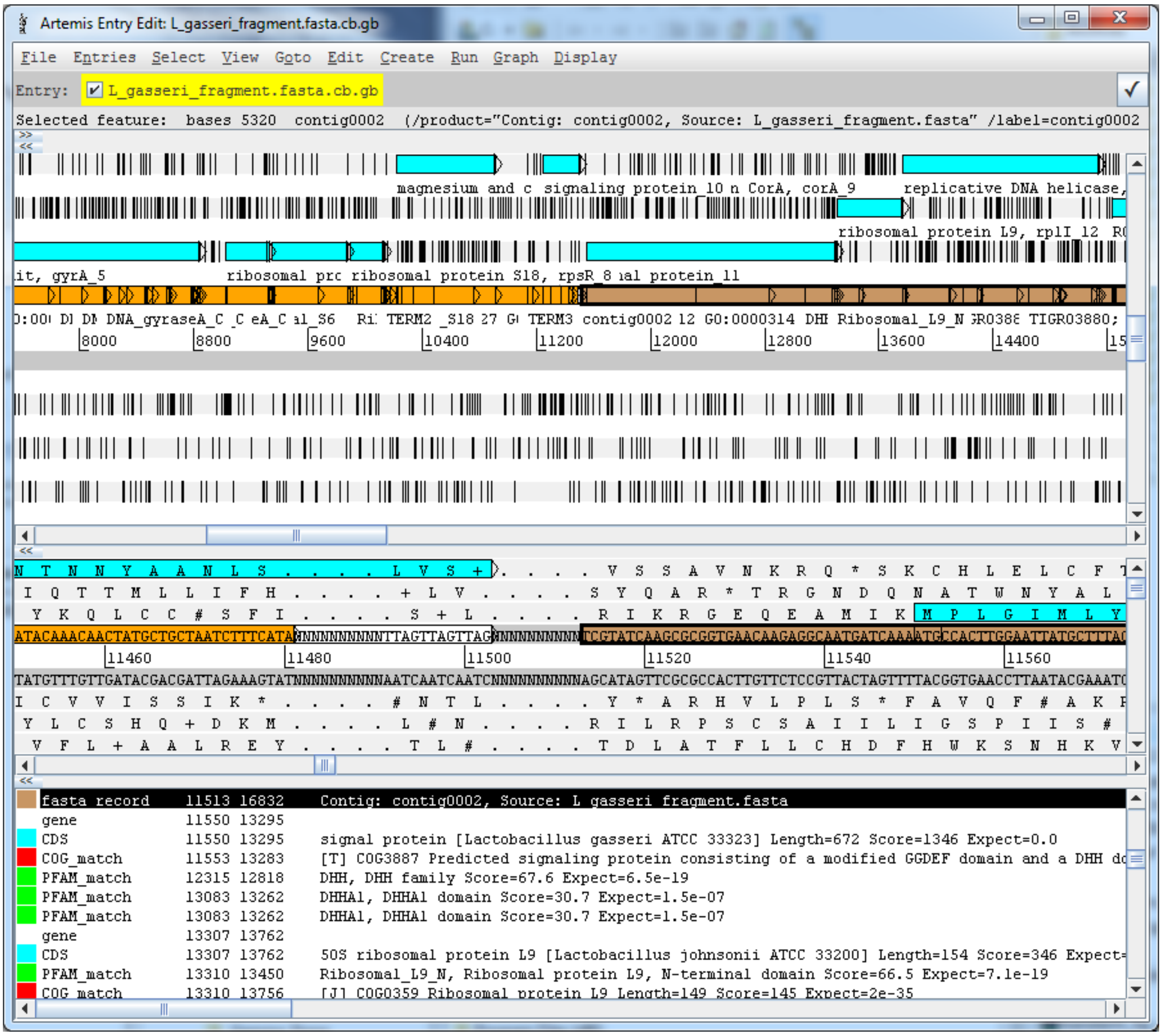

(b) **‘gene’ feature**: only contains the most basic information, the gene and locus_tag qualifiers. The ‘gene’ feature serves to provide the shortest possible name for a given ORF (e.g. ‘dnaA’).

Changing annotation is as easy as changing the ‘gene’ qualifier in the ‘gene’ and ‘CDS’ features, without changing the assigned ORF number at the end of the annotation (e.g. ‘_1’).

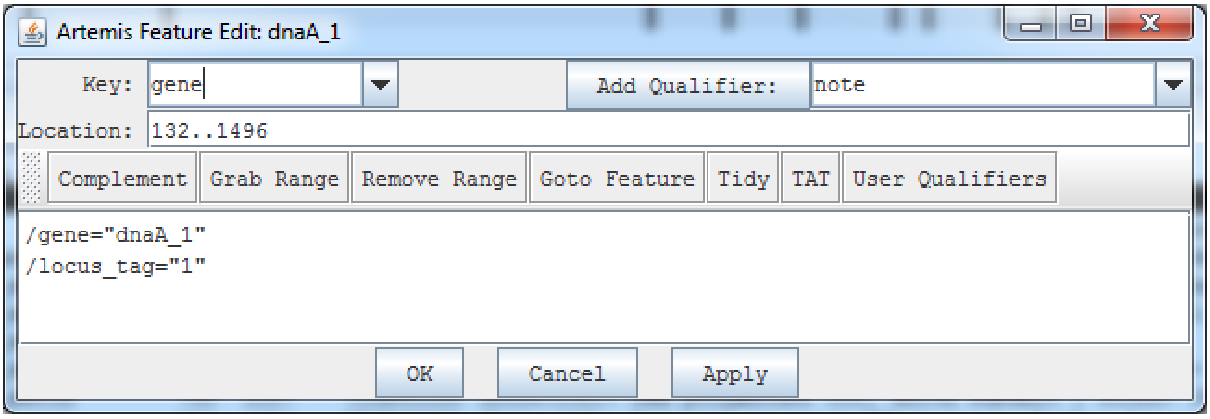

(c) **'CDS' feature**: The 'CDS' feature shows information on the best Blast hit in the 'product' qualifier, a more verbose description(e.g. DNA replication protein, DnaA) of the predicted ORF annotation in the 'gene' qualifier, the locus_tag, accession number of the best Blast hit in the 'note' qualifier and the deduced amino acid sequence in the 'translation' qualifier.

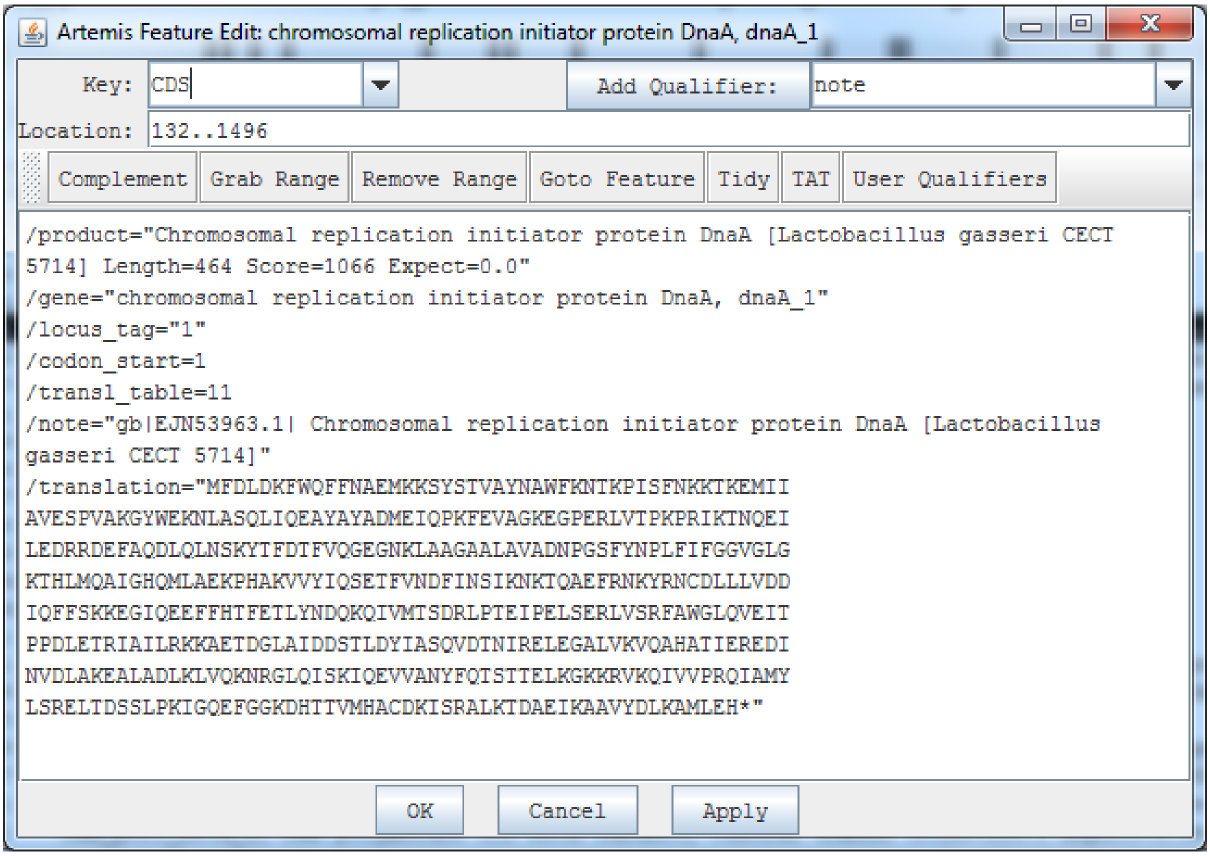

(d) **‘COG_match’ feature**: The COG_match sows the best COG hit found and provides information on the high level COG category (‘label’ qualifier’) and a human readable descriptor of the actual COG hit found, including values for Length, Score and e-value. Individual COG results for each ORF can be found in the ‘COG_database’ results folder and may be retrieved manually or from within the Artemis genome browser.

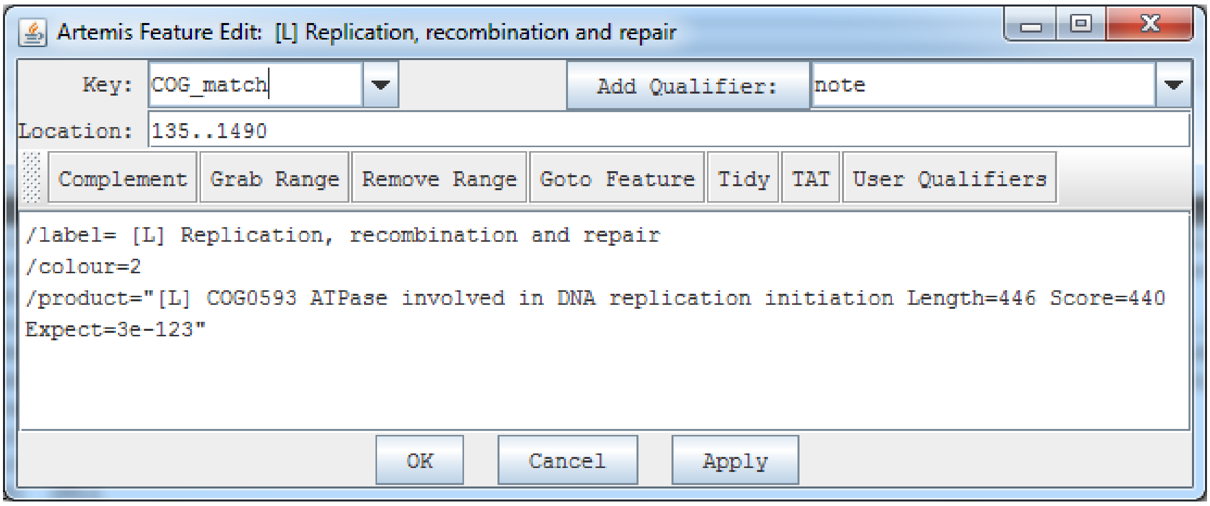

(e) **'PFAM_match' feature**: Pfam hits are shown as position specific domains. The number of Pfam domains per ORF can be modified in the GAMOLA2 setup in the 'Main Options' tab. Pfam features include a short descriptor label, notes on the quality and nature of the respective Pfam alignment, a more verbose Pfam descriptor and, when selected, a comprehensive Interpro description of the respective Pfam match. Finally, the respective quality scores are listed.

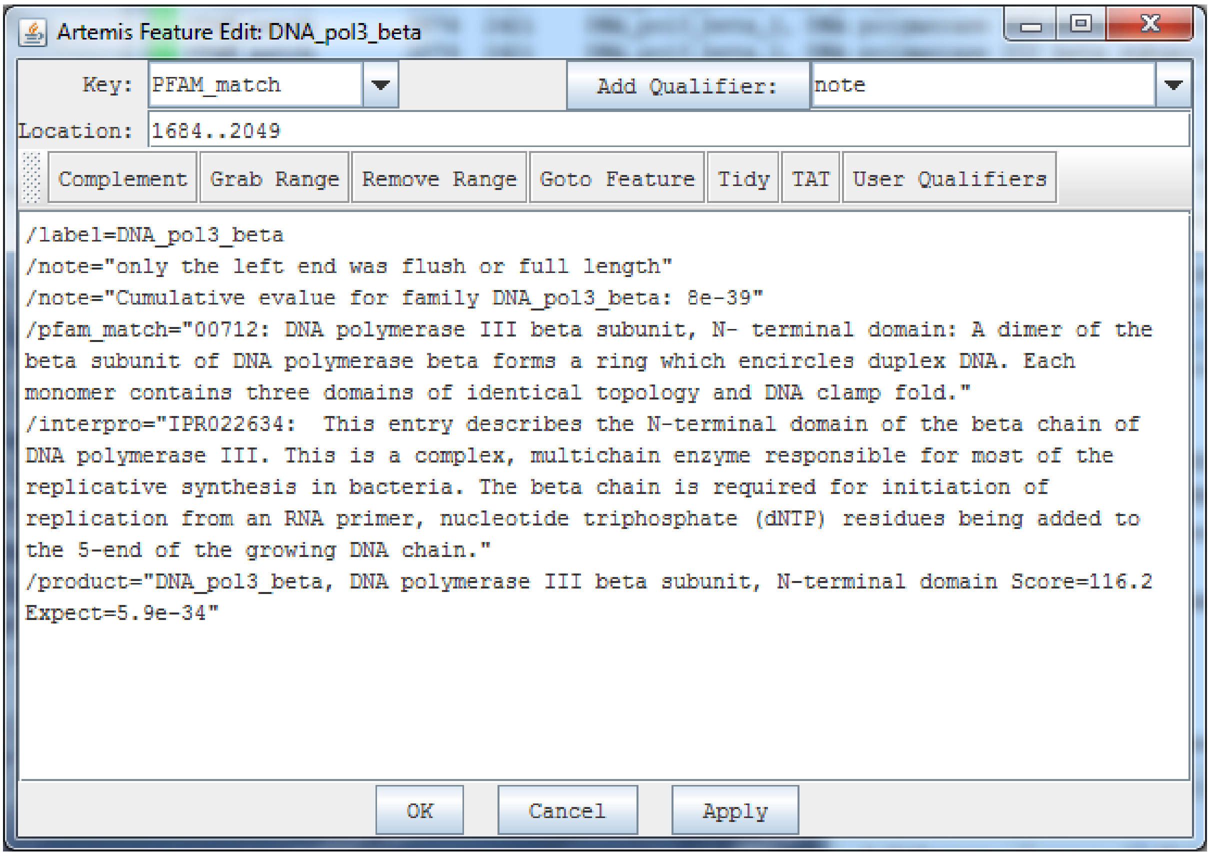

(f) **‘TIGRfam_match’ feature**: TIGRfam hits are shown as position specific domains. The number of TIGRfam domains per ORF can be modified in the GAMOLA2 setup in the ‘Main Options’ tab. The TIGRfam feature shows a short gene name and associated Gene Ontology terms, the respective E.C. number (where applicable), the respective TIGRfam hit, role number and descriptor with associated quality scores, the cumulative e-value and a verbose TIGRfam descriptor. In addition, an optional Gene Ontology definition is provided.

If selected in the ‘Main Options’ tab, the best TIGRfam feature will be used for gene annotation. Where possible, the short gene descriptor will be used as ‘gene’ feature name and the more descriptive TIGRfam term describes the ‘CDS’ feature.

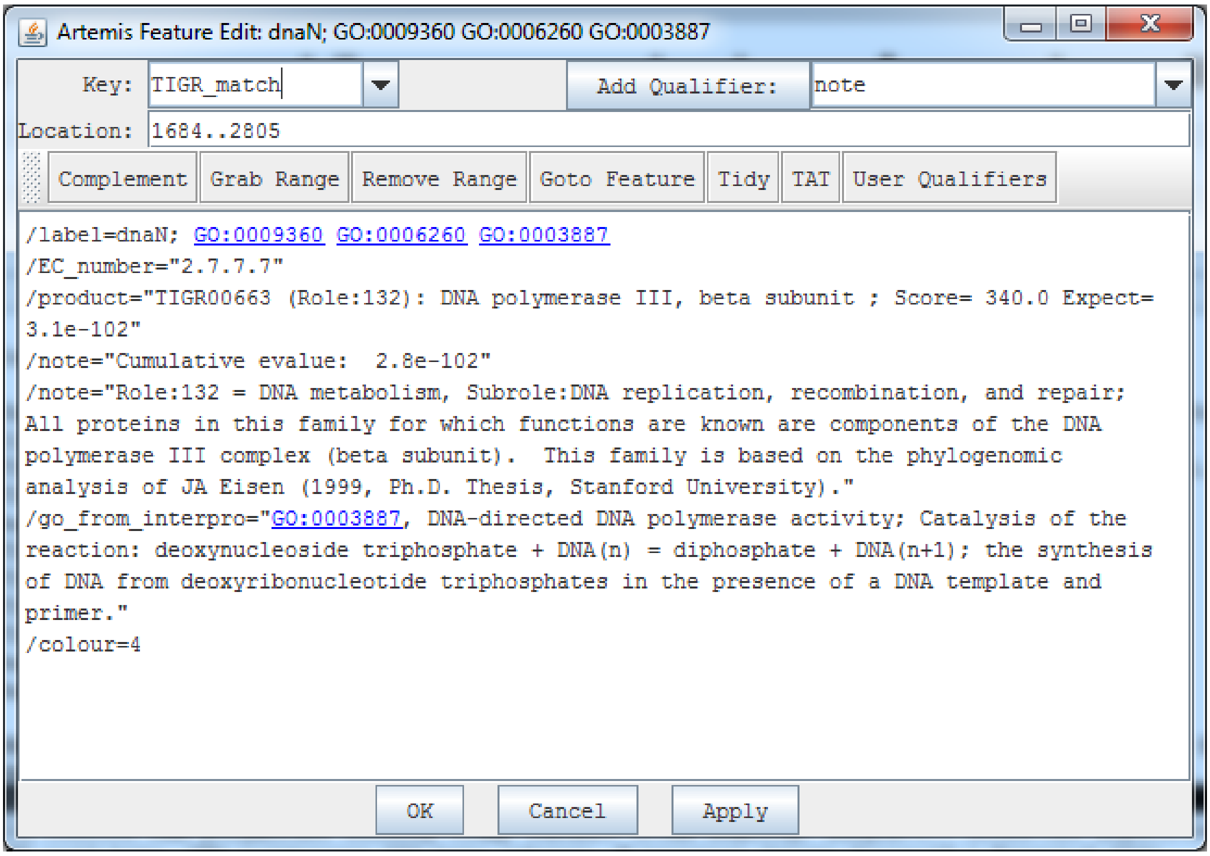

(g) **‘tRNA’ feature**: The tRNA feature provides a dedicated locus_tag and the predicted tRNA specifity. Where no specificity was predicted, the product will show ‘tRNA-OTHER’ and a ‘/pseudo’ qualifier is added to the feature entry.

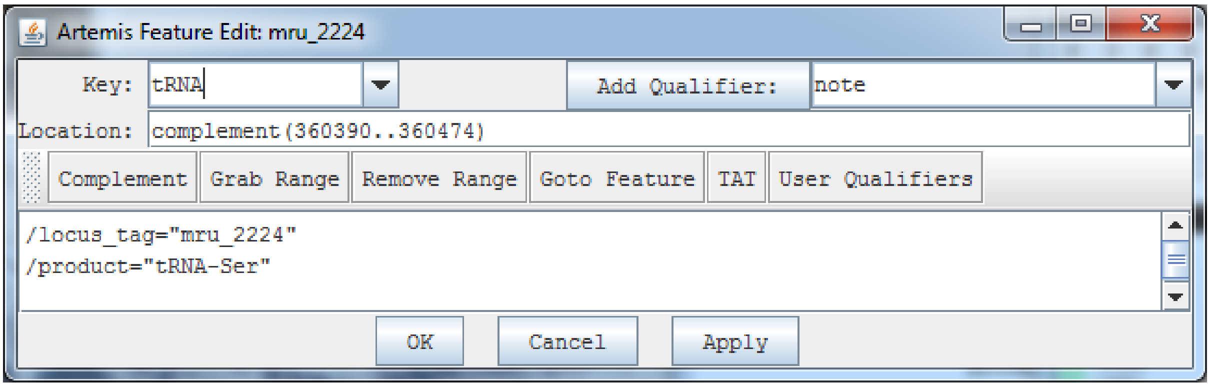

(h) **'rRNA' feature**: The rRNA feature indicates the deduced location of microbial ribosomal RNA genes (5S, 16S and 23S), based on their respective Blast alignment positions. Where incomplete alignments are found, the position of the respective full length rRNA gene will be extrapolated. This approach enables the prediction and location of partial rRNA genes in draft genomes, in particular when located at contig boundaries or in cases of misassemblies.

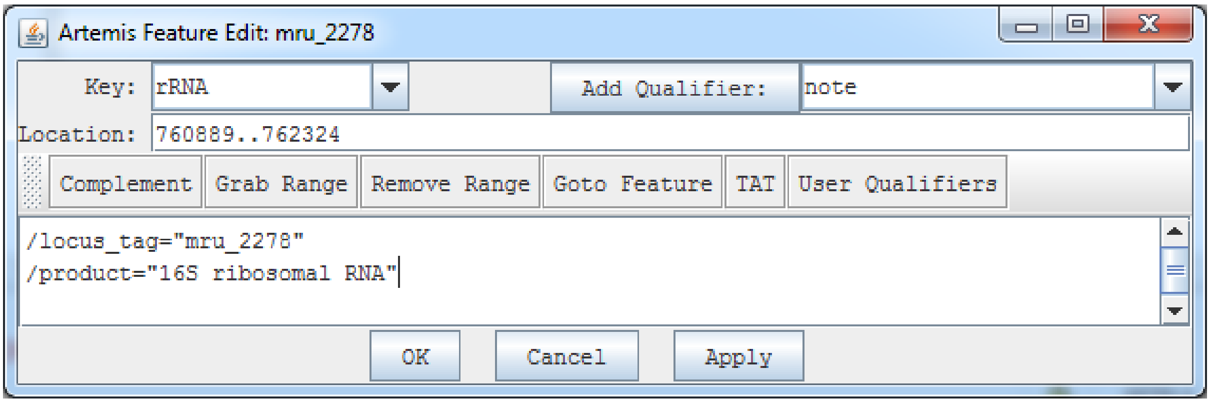

(i) **'non-coding RNAs' feature**: Non-coding RNA (ncRNA) represents a new class of RNA molecules that are not involved in protein synthesis, but often contain other information or carry out a variety of biological functions, such as forming an RNA regulatory network. While most ncRNA classes remain uncharacterised, there is an increasing number of ncRNAs with a confirmed biological role. ncRNA entries may have three different feature keys: (i) rRNA: for ribosomal RNA genes, (ii) tRNA: for transfer RNAs and (iii) ncRNA: for all other non-coding RNA classes. rRNA and tRNA ncRNA hits may be excluded to avoid duplication of hits if dedicated tRNA and rRNA analyses were selected.

The ncRNA feature lists the short feature name (label), the overall feature score (the higher the better), the RF number and associated function, detailed baseline and element scores and, where available, a verbose description of the ncRNA biological function.

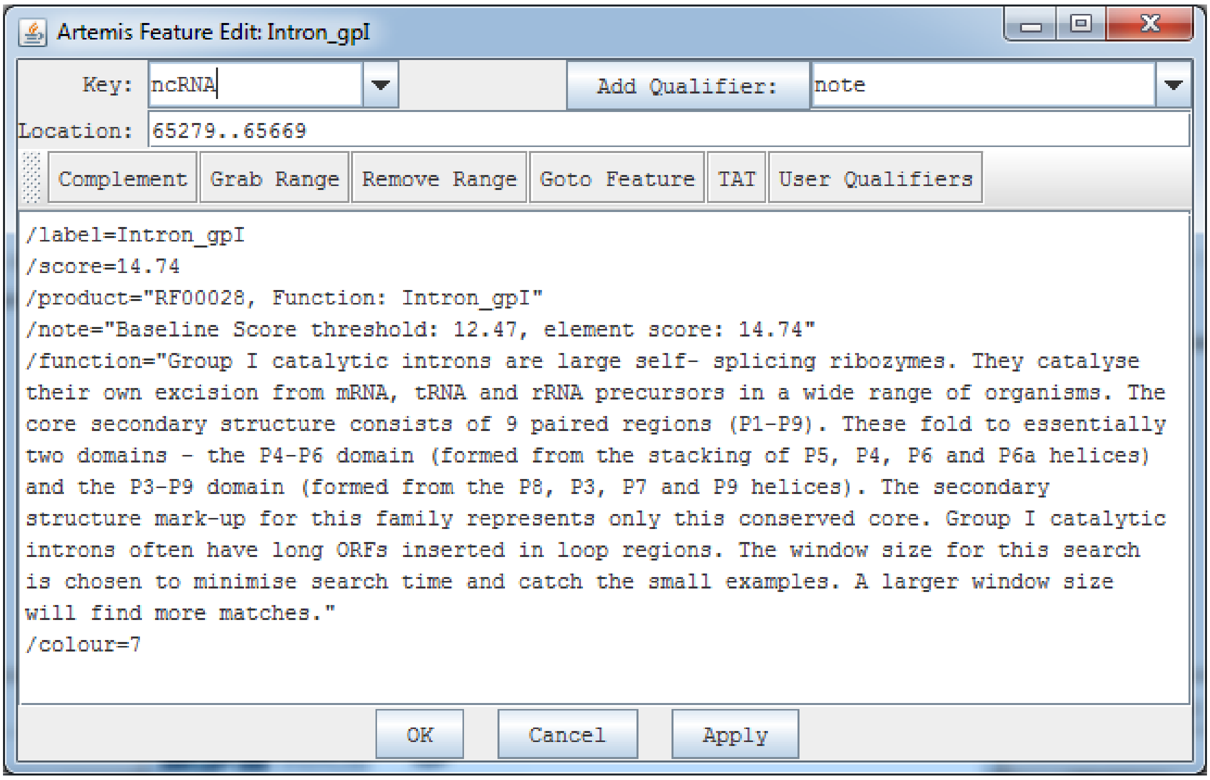

(j) **'TMM' feature**: Transmembrane helices are shown either as a single entry or as a position specific joint feature, whereby the position of individual transmembrane helices is indicated. Additional information provided is the presence of a potential N-terminal signal sequence (this information is complementary to the dedicated SignalP search) and the likelihood of the N-terminus being located in the cytoplasm. A high or low value implies an intra- or extracellular protein configuration, whereas a mid-range value indicates an uncertain orientation.

For each predicted ORF, full results can be found in the 'TMHMM_database' results folder. Text based output are shown in the '.plp' (detailed output) and '_tmhmm' (summary) files. Graphical visualisation is provided via '.eps' and '.gnuplot' files.

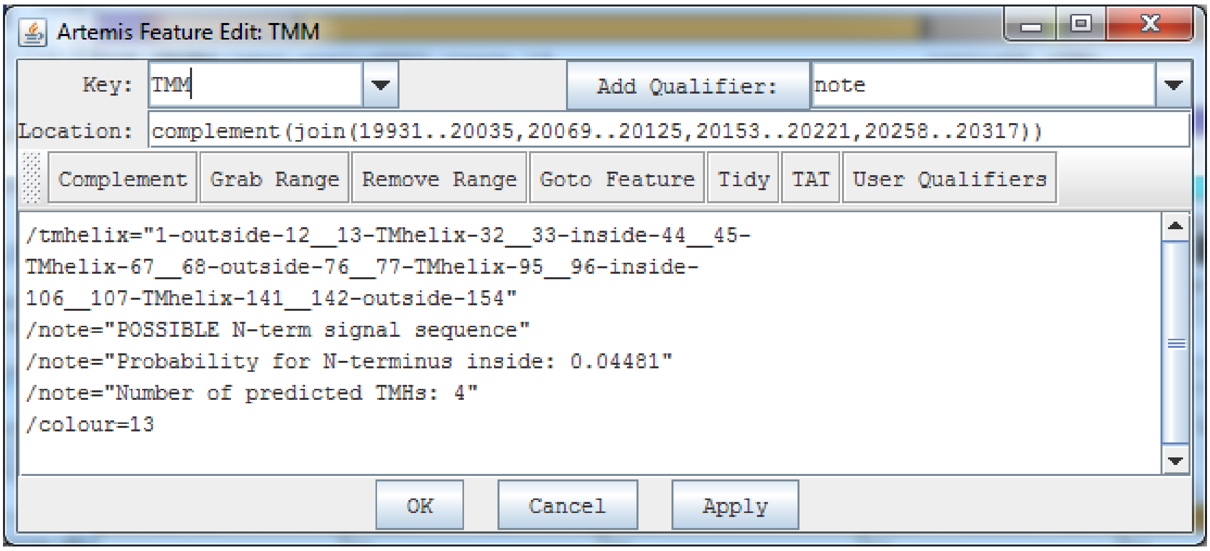

(k) **‘SignalP’ feature**: The presence and location of a predicted signal peptide cleavage site is shown. The position of the predicted cleavage site is shown as well as the likelihood of the prediction and the cleavage probability.

For each predicted ORF, full results can be found in the ‘SignalP_database’ results folder, where each ORF is given a separate directory. The amino acid sequence in FASTA format ‘.seq’ and a detailed results file ‘output.txt’ are provided.

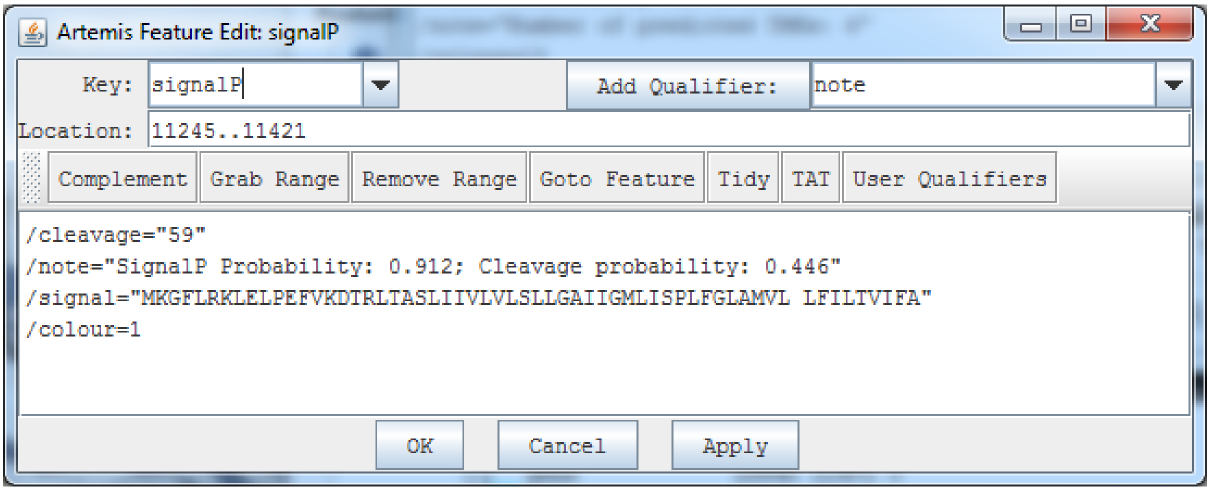

(l) **'Terminator' feature**: The presence and orientation of rho-independent terminator structures is shown, including the overall confidence score (range from 0 to 100 with 100 being the highest score). The 'label' qualifier shows the name of the terminator structure (usually 'TERM' plus a sequential number), whereas overlaps, opp_overlaps and the nucleotide sequence (including tail, stem and loop) are shown in two 'note' qualifiers.

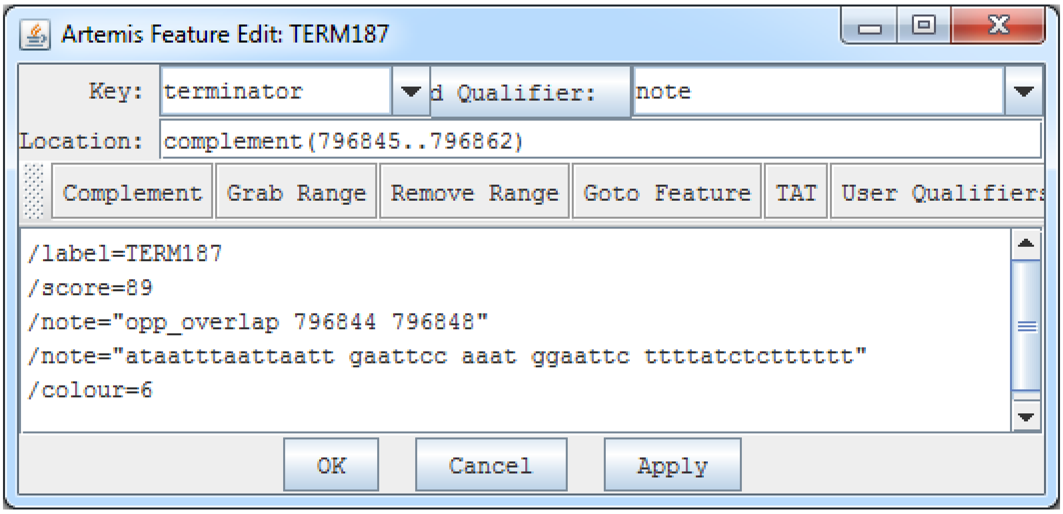

(m) **‘CRISPR’ feature**: CRISPRs have recently seen a lot of interest and are widely spread in microbial genomes. They may serve as an indicator to the extent of viral pressure encountered. CRISPRs are indicated as a joined feature, whereby individual repeats are linked. The overall CRISP structure is shown in the ‘CRISPR’ qualifier and the corresponding nucleotide sequences in the respective ‘note’. General properties such as range, number and average lengths of the repeats and spacers are indicated in a separate ‘note’ qualifier.

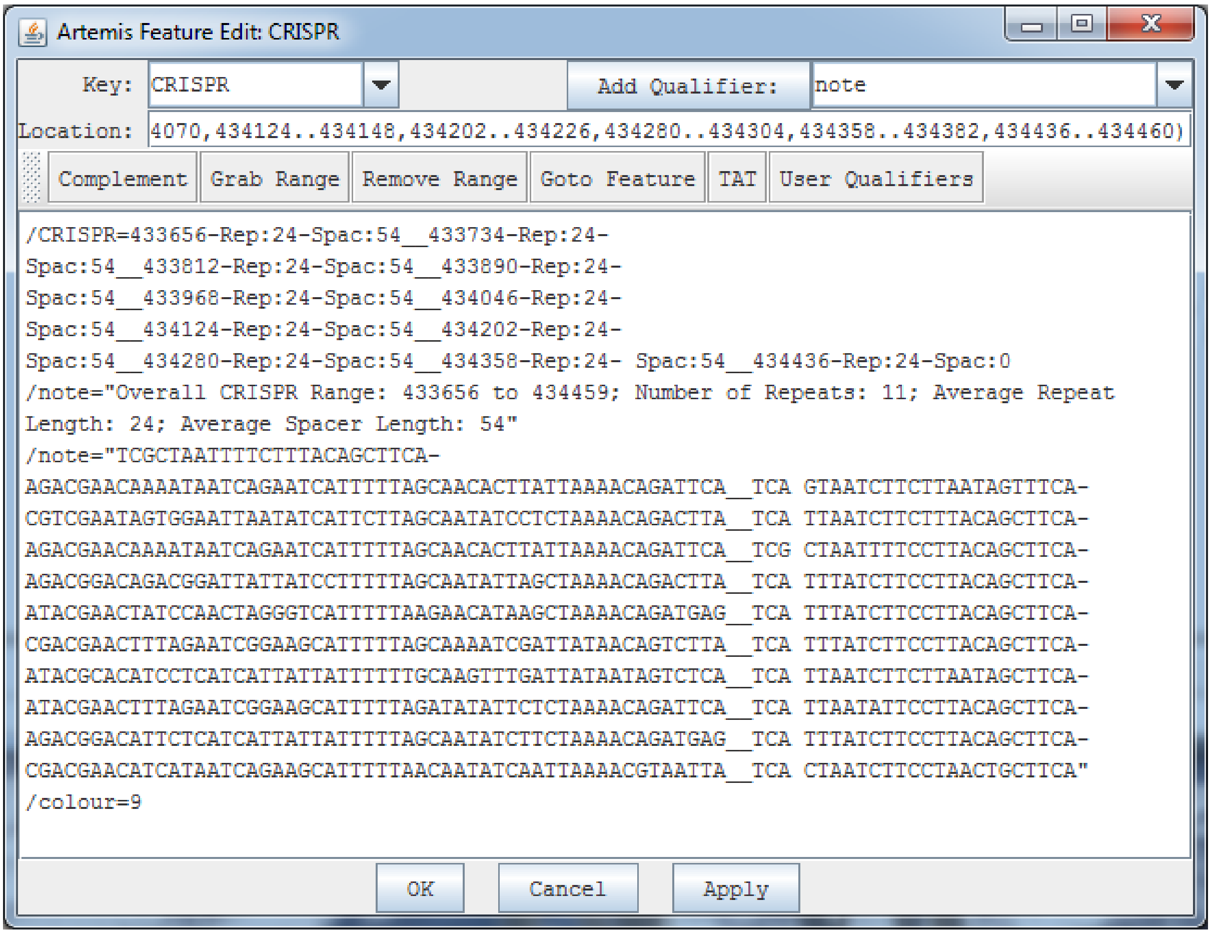

(n) **'Vector contamination' feature:** Vector contamination is particularly a problem in draft phase genomes where sequence reads may have been insufficiently cleaned. Vector matches are shown as 'Vector_match' features and a verbose description of the respective hit is shown in the 'product' qualifier. Where multiple alignment blocks were found in the BlastN analysis, a joint feature not exceeding 10,000nt is shown. Individual section scores, the total score (sum of section scores) and the resulting hit quality are shown in a 'note' qualifier. Care should be taken to eliminate false positive hits, in particular with respect to short internal (>25nt distance from contig or sequence termini) matches to vector sequences.

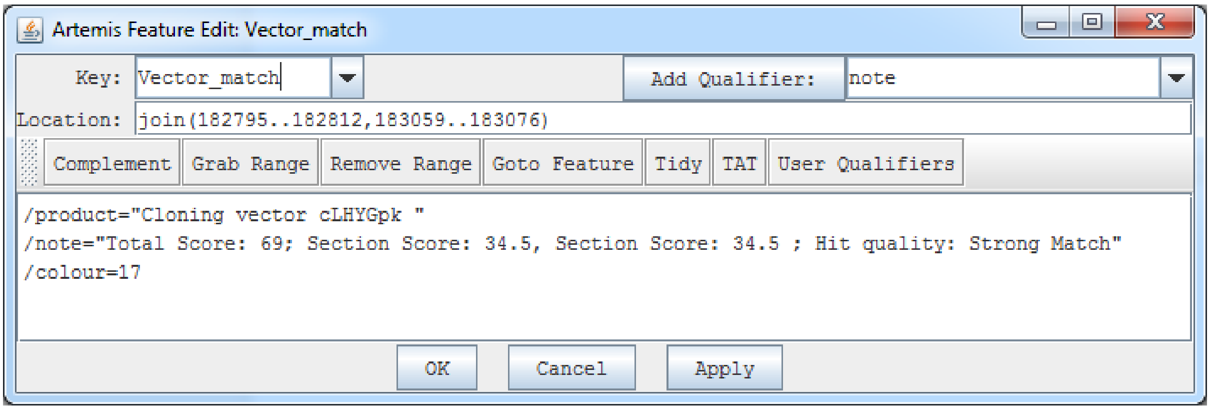

5.2.3 *Retrieving underlying Results*

GAMOLA2 integrates the respective best hits against selected databases into the Genbank file. In many instance, however, it is important to also investigate less significant hits (above the chosen thresholds) for either consistency or common functional themes. In particular for 'conservered hypothetical' ORFs, weak, but thematically consistent hits across functional (and structural) databases can give clues of the biological role of these ORFs and may often allow the designation of a tentative functional annotation.

The modified version of Artemis used here offers this functionality for Blast, COG, PFam and TIGRfam hits. Other hits may be included in future updates when requested, but experience so far has shown that expanded results of structural database hits were less frequently investigated tham those of functional databases.

This functionaliy requires the 'Genbank file – Result folder' structure to be maintained:

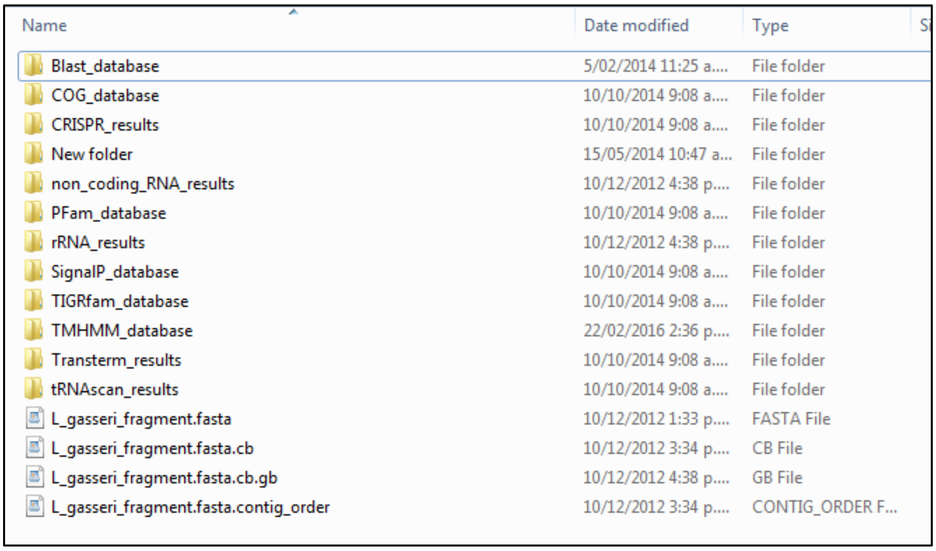

Name	Date modified	Type
Blast_database	5/02/2014 11:25 a....	File folder
COG_database	10/10/2014 9:08 a....	File folder
CRISPR_results	10/10/2014 9:08 a....	File folder
New folder	15/05/2014 10:47 a...	File folder
non_coding_RNA_results	10/12/2012 4:38 p....	File folder
PFam_database	10/10/2014 9:08 a....	File folder
rRNA_results	10/12/2012 4:38 p....	File folder
SignalP_database	10/10/2014 9:08 a....	File folder
TIGRfam_database	10/10/2014 9:08 a....	File folder
TMHMM_database	22/02/2016 2:36 p....	File folder
Transterm_results	10/10/2014 9:08 a....	File folder
tRNAscan_results	10/10/2014 9:08 a....	File folder
L_gasseri_fragment.fasta	10/12/2012 1:33 p....	FASTA File
L_gasseri_fragment.fasta.cb	10/12/2012 3:34 p....	CB File
L_gasseri_fragment.fasta.cb.gb	10/12/2012 4:38 p....	GB File
L_gasseri_fragment.fasta.contig_order	10/12/2012 3:34 p....	CONTIG_ORDER F...

When moving the result files to a new location, always copy the entire file/folder structure.

Accessing Result files

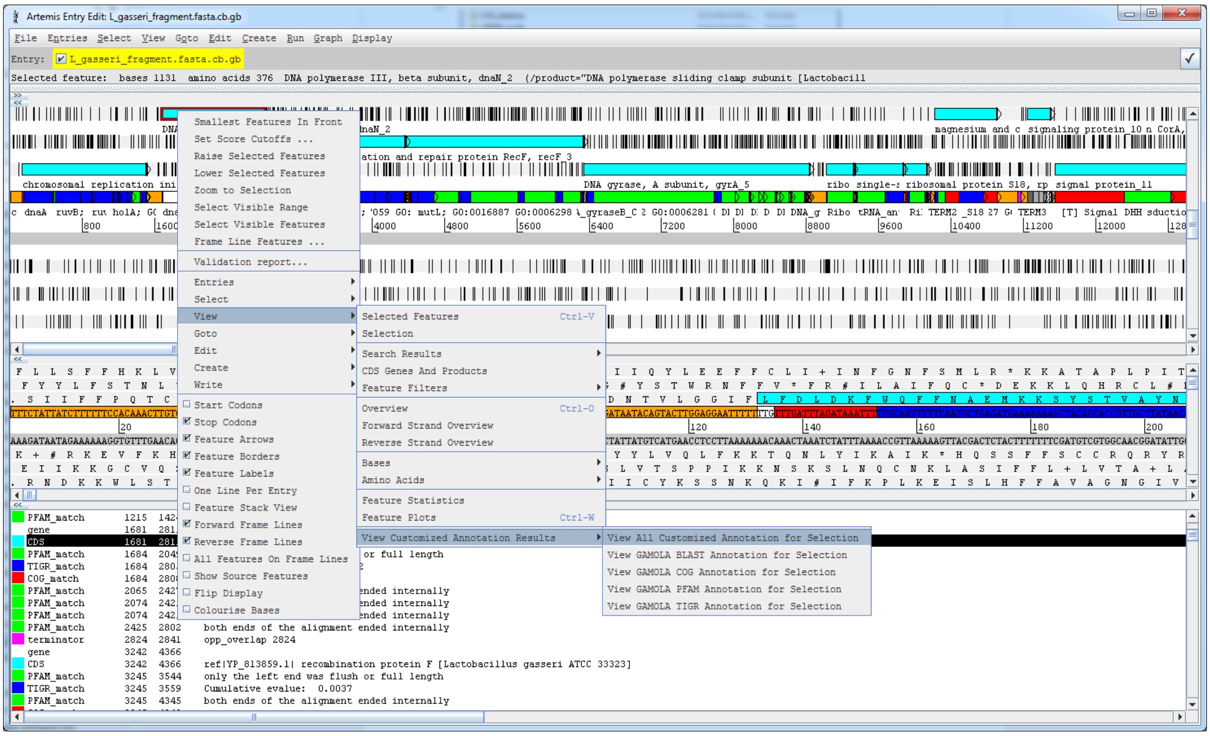

(i) Select a 'gene' or 'CDS' feature in any of the three Artemis panels.

(ii) Right-click on the selected feature and choose 'View -> View Customized Annotation Results -> View…'

(iii) 'View all results' will retrieve Blast, COG, PFam and TIGRfam results for the selected feature, while the other viewing options will retrieve results for individual functional database hits.

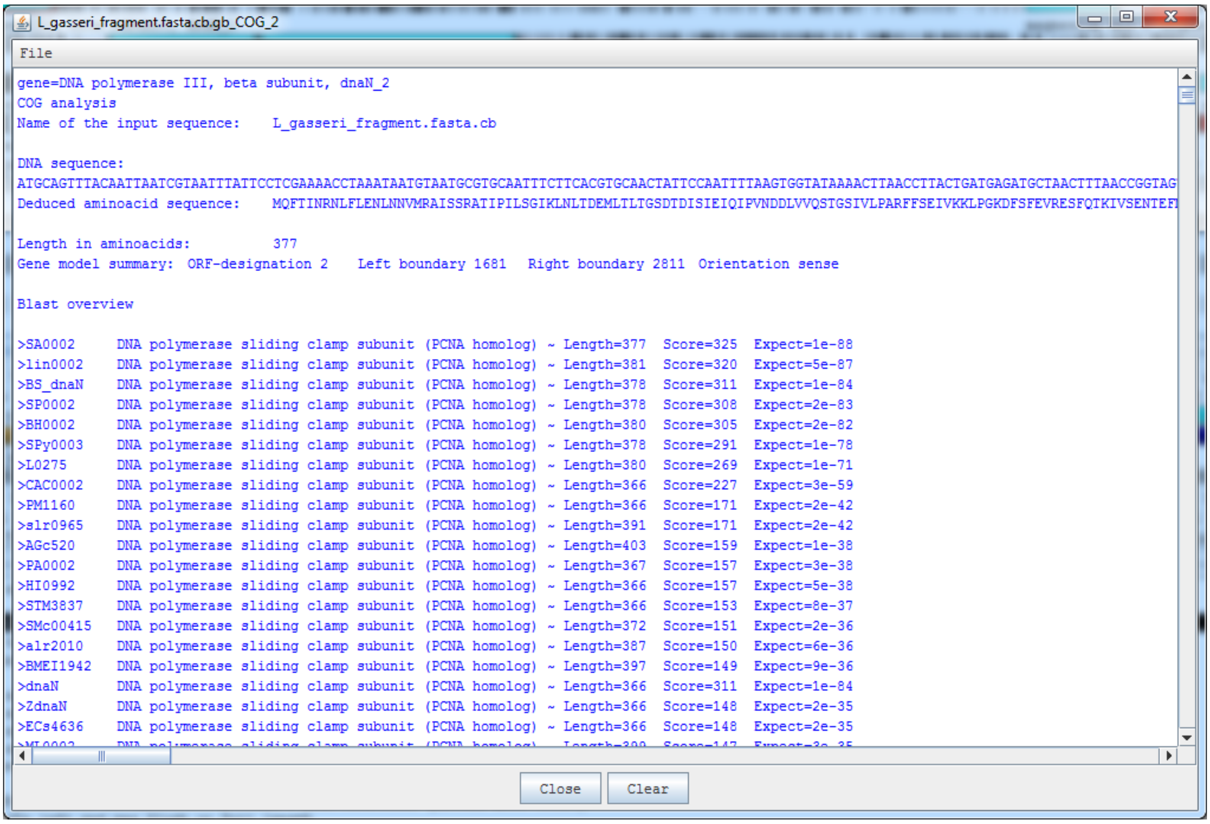

Example: COG results for a predicted DNA polymerase. The header comprises the current gene annotation and associated ORF number, the type of analysis (COG), the name of the respective input file, the DNA and amino-acid sequences of the ORF, a short gene model summary, a modified Blast summary that shows the COG accession number, the translated COG descriptor and the Blast quality parameters. Below the Blast summary, the Blast alignments are shown.

Similar result outputs are created for Blast, Pfam and TIGRfam results.

Note: At present, structural database hits can be investigated individually by manually retrieving respective result files from within their folders. Each result file name comprises the respective GAMOLA ORF number in its file name.

6 Additional GAMOLA2 functionality

Aside from providing functional annotations for draft and completed microbial genomes, GAMOLA2 offers a range of additional modules that extent its functionality.

6.1 Databases

6.1.1 Create Custom databases

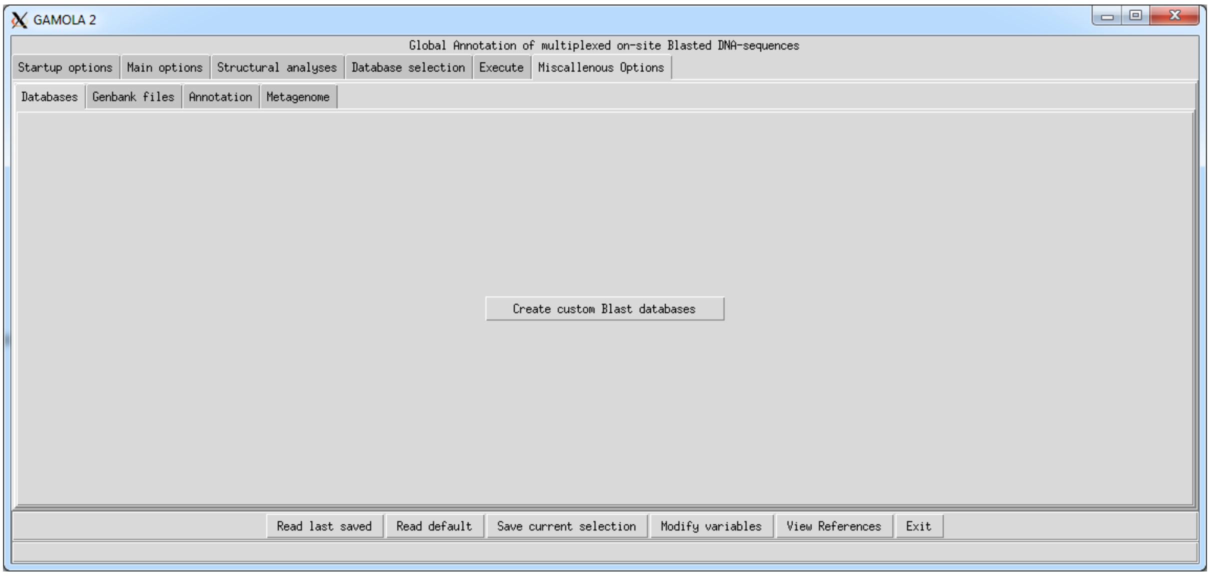

Custom Blast databases provide the opportunity to tailor analyses to defined datasets and are useful for many applications – both in GAMOLA2 Blast database and in other applications or standalone Blast.

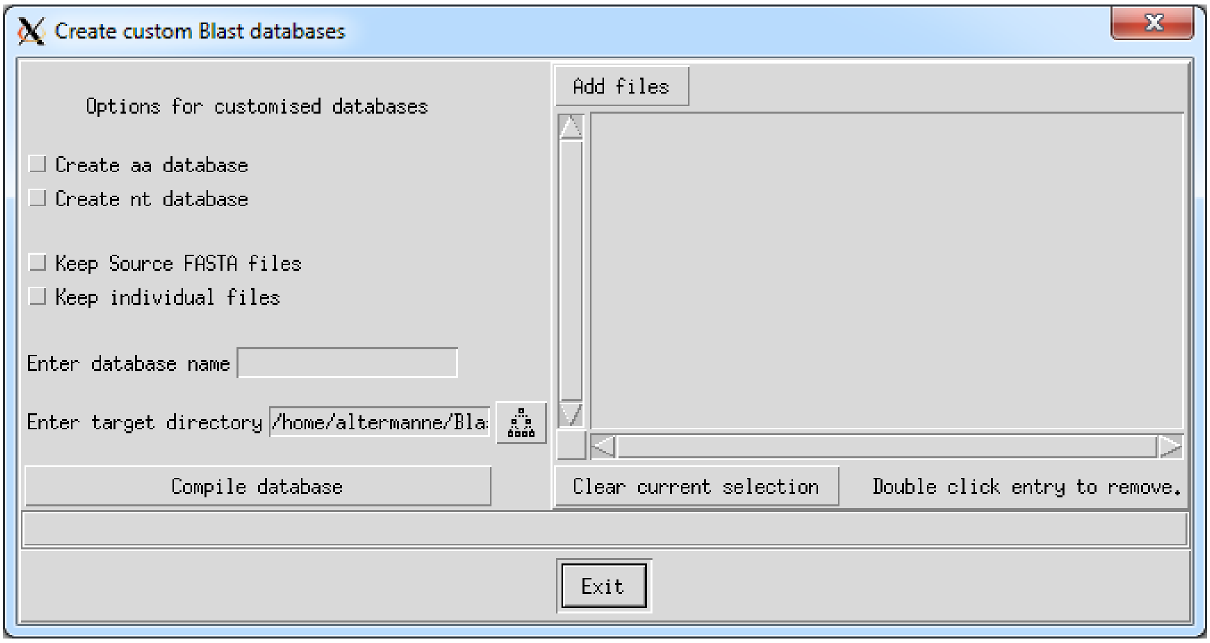

The module creates both nucleotide and amino-acid Blast databases and accepts FASTA, msFASTA and annotated Genbank files as input.

(i) Add input files by clicking on the ‘Add files’ button and select appropriate input files. (ms)FASTA files must be in the correct nt or aa format, Genbank files will be parsed as required. Multiple files can be selected and each file will be incorporated into the resulting Blast database. To remove all entries from the list, click on the ‘Clear current selection’ button. Individual entries can be removed by double click on the respective entry.

Note: Parsing entry files is relatively slow and creating large, complex custom databases can take a while. This is because GAMOLA2 carries out a number of safety and integrity checks on each sequence, including translating respective sequences from Genbank file gene models), rather than assuming that all input files are correct and without error.

(ii) Select either 'Create aa database' or 'Create nt database' option. Both options are mutually exclusive on only one can be selected. In case a nucleotide database is selected, an additional choice is required for Genbank input files:

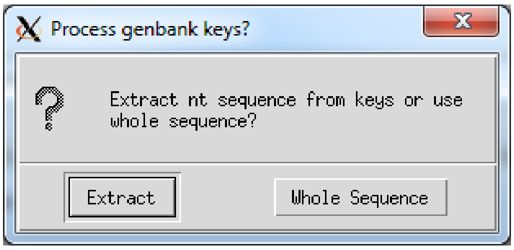

This option will either retrieve nucleotide sequences for the embedded gene model ('Extract') or the entire sequence provided in the Genbank file will be used as a single entry in the Blast database ('Whole Sequence').

(iii) Two optional parameters can be set: (a) 'Keep source FASTA files': this will leave the individual FASTA files accessible after the Blast database has been created and (b) 'Keep individual files': this option will retain original msFASTA and msGB files. If deselected, those multi-entry files will be deleted and replaced with their respective single entry equivalents.

(iv) Choose a database name by typing the desired name of the custom Blast database into the field. No file path is needed here.

(v) Select the directory where the custom Blast database will be saved. Either manually type in the path or click on the file tree button on the right to choose the appropriate directory.

(vi) Compilation of the new custom Blast database is started by clicking on the 'Compile database' button. Depending on the number and complexity of the input files, the process can take some time. This is mainly due to internal error checking and validation processes to fail-safe resulting databases.

The output will be custom, indexed Blast databases saved in the selected directory that are available for use in GAMOLA2 and other software tools.

6.2 *Genbank files*

The main purpose of GAMOLA2 is to generate high-quality, annotated Genbank files. Yet, there are common tasks, such as re-orientating a Genbank file or preparing the sequences for submission to NCBI that can be tedious and benefit from a certain level of automation.

6.2.1 *Rotate single Genbank file*

Particularly draft phase genomes are often randomly oriented, while most published genomes adhere to a common denominator for their respective start positions (e.g. the origin of DNA replication, dnaA or similar marker genes). This module enables the rotation of annotated Genbank files to a desired position of choice.

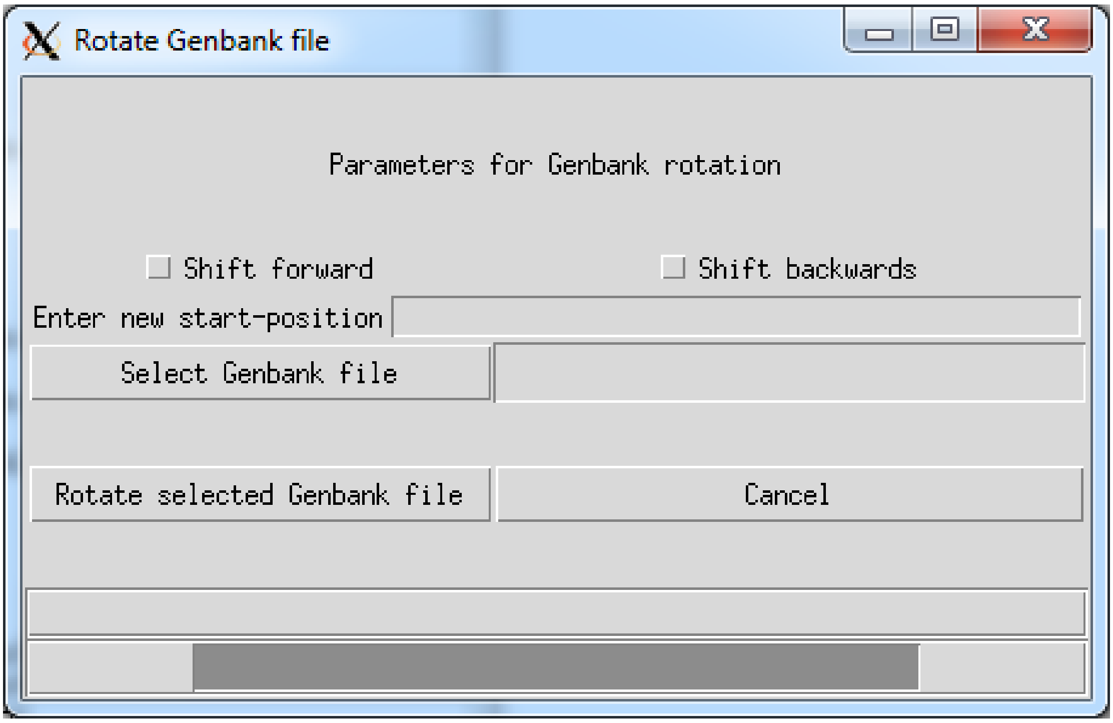

(i) The Genbank file can be rotated either clockwise or counterclockwise, select the option of preference.

(ii) Enter the new start position [nt] in the respective field

(iii) Select the target Genabank file by clicking on the 'Select Genbank file' button

(iv) Start the rotation by clicking on the ‘Rotate selected Genbank file’ button or ‘Cancel’ the operation.

The new rotated Genbank file is saved into the same directory as the original Genbank file, with the ‘_rotated’ nominator added to the Genbank file name.

6.2.2 *Prepare Genbank files for Sequin submission*

Preparing annotated microbial genomes (or other sequences) for submission to NCBI via the Sequin tool (http://www.ncbi.nlm.nih.gov/Sequin/) can be notoriously time consuming. To streamline this process and automate the entry of features into Sequin, GAMOLA2 offers a Sequin Parser module.

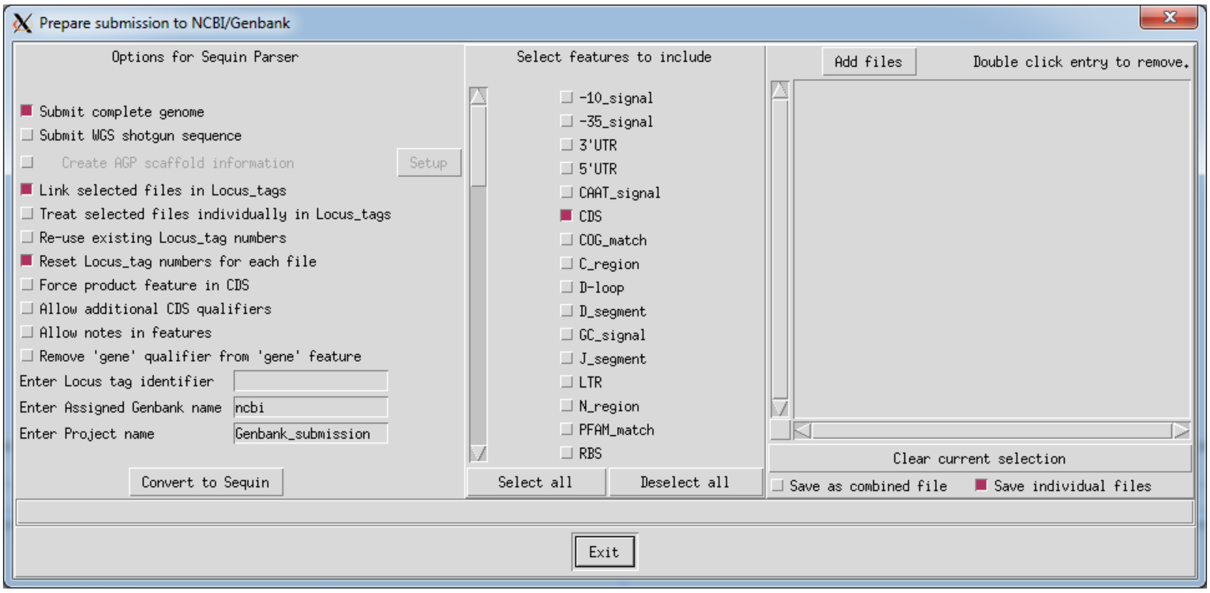

The module supports both submission of closed and draft phase sequences, as well as multiple Genbank files for submission.

Converting Genbank file(s) for Sequin:

(i) *Source files*: Select annotated Genbank files ready for submission by clicking on the 'Add files' buttons. The entire selection can be cleared via the 'Clear current selection' button,

while individual entries can be removed via double-click.

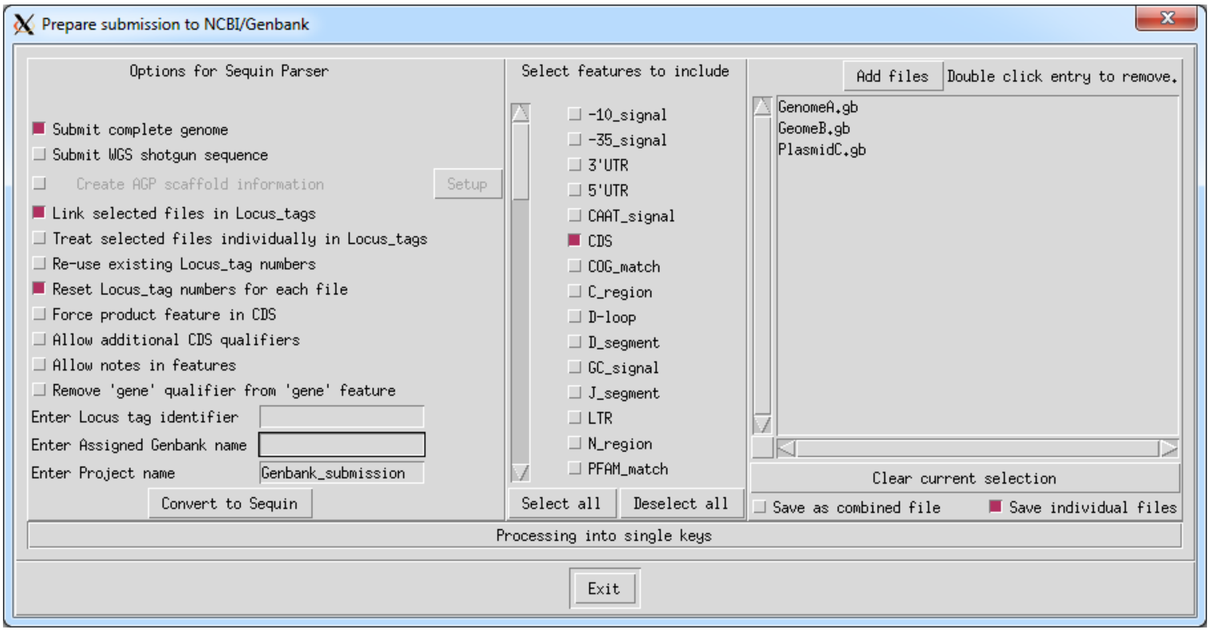

(ii) Where multiple Genbank files have been selected, the project can either treat these files as individual entities ('Save individual files' checkbox) and create respective separate output files for each input file or combine all input files into a single submission ('Save as combined file'). Combining all input files into a single submission results in one FASTA and one feature table file. Individual entries are separated by a header derived from the Genbank file.

(iii) Results comprise a FASTA sequence file and a Sequin compatible feature table. These output files will be saved in the './Results/Sequin_submission' folder. Subfolders are created as appropriate. For draft genomes ("WGS shotgun sequence") a third '.agp' table will be created and saved.

(iv) Select whether complete ('Submit complete genome') or draft ('Submit WGS shotgun sequence') genomes are submitted. Only one choice per submission is allowed.

(v) If draft genomes are submitted, scaffold information may optionally be provided in form of a simple text file that lists the names and order of contigs present in the draft genome. If the contig order is provided, tick 'Create AGP scaffold information' and select

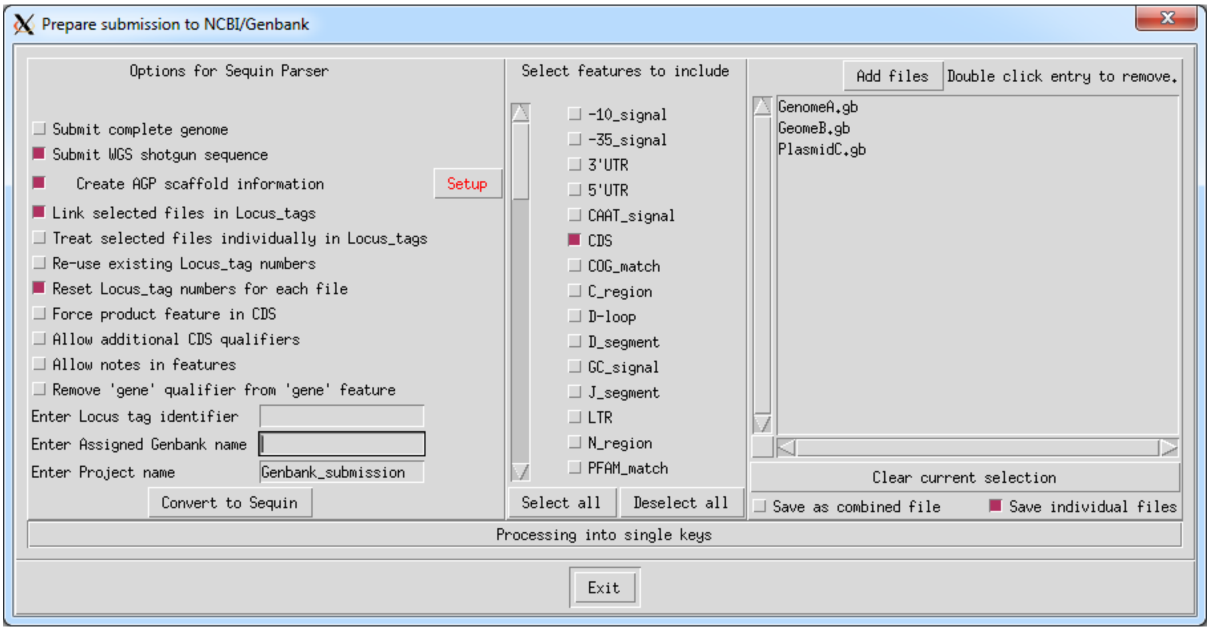

the corresponding mapping file by clicking on the 'Setup' button.

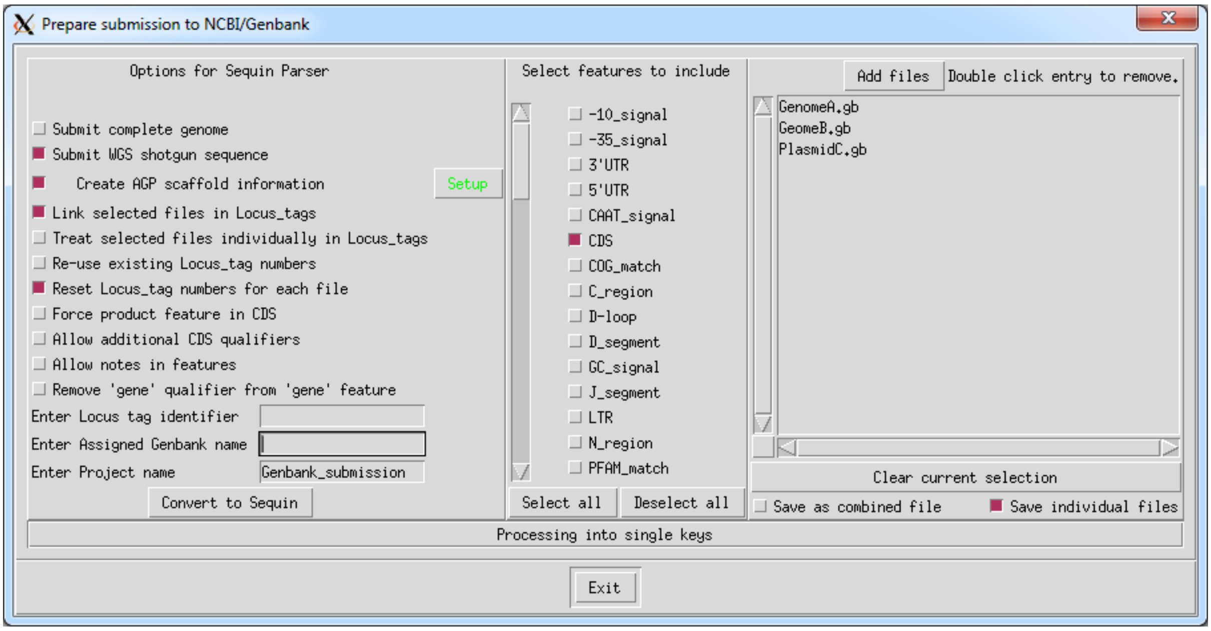

Once the mapping file has been selected, the 'Setup' button will change colour to green.

(vi) Example of AGP scaffold text file (file name: 'mapped_contig_order.txt'):

contig00001

contig00002

contig00003

contig00005

contig00010 …

<u>Current Limitations for AGP files</u>:

-- Support of only one type of link-evidence for draft genomes. If other links are used, they can be easily added by a simple search&replace function in any text editor.

-- Only single draft genome submissions are supported. Do not chose more than one input Genbank file for draft genomes with mapping files.

(vii) *Locus tags*: If multiple files are selected, locus tags can either be linked throughout all files (tick: 'Link selected files in Locus_tag') or calculated individually for each file (tick: 'Treat selected files individually in Locus_tag'). These options are mutually exclusive. When locus tags are linked, a roman numeral is added to the locus tag, sequentially increasing for each file in addition to the regular Arabic counter.

Input files will be processed in the order in which they were added.

Example:

In GenomeA, the locus tag will be: 'locus_tag'_I_0001, 'locus_tag'_I_0002, etc.

In GenomeB, the locus tag will be: 'locus_tag'_II_0001, 'locus_tag'_II_0002, etc.

In GenomeC, the locus tag will be: 'locus_tag'_III_0001, 'locus_tag'_III_0002, etc.

When input files are treated individually, the roman numeral is omitted.

Note that the same locus tag descriptor is used for all files.

(viii) *Re-using locus tags*: In some cases, Genbank files already have designated locus tags. By selecting the 'Re-use existing Locus_tag numbers' option, existing locus tags numbers will be re-used. Missing locus tags will be filled in with the expected locus tag number.

(ix) *Locus tag counting*: When multiple input files are selected, locus tags can be enumerated for each file individual (tick: 'Reset Locus_tag numbers for each file'), starting each input while with locus tag number '1'. Deselecting this option forces a continuous locus tag count across all input files.

(x) *CDS annotation*: CDS features may have both '/gene' and '/product' qualifiers. By default, the '/gene' qualifier is used to obtain a CDS annotation. However, selecting the 'Force product qualifier in CDS' option will preferentially attempt to use information in the '/product' qualifier for annotation.

(xi) *Additional CDS features*: By default, CDS features comprise 'protein_id' and 'product' qualifiers only. By selecting the 'Allow additional CDS qualifiers' option, other qualifiers (if present in the source Genbank file) can be included. Select desired additional options from the new pop-up window, then click 'OK'. To select all option, click on the 'Select all' button, then on 'OK'.

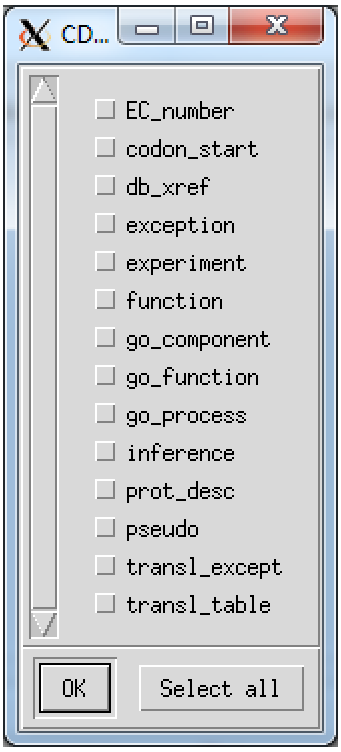

(xii) *Notes in CDS feature*: Occasionally, notes are used to add additional information to the annotation. Select 'Allow notes in CDS features' to add these notes to the Sequin table.

(xiii) *Omit gene feature annotation*: Select the 'Remove 'gene' qualifier from 'gene' feature' option to simplify the 'gene' feature annotation. This option removes the annotation and leaves only the locus tag in the Sequin table.

(xiv) *Locus tag ID*: enter the Locus tag identifier in the appropriate text field.

Note that only one ID is currently supported per Sequin submission. Multiple input files will receive the same locus tag, distinguished by a roman letter if selected above.

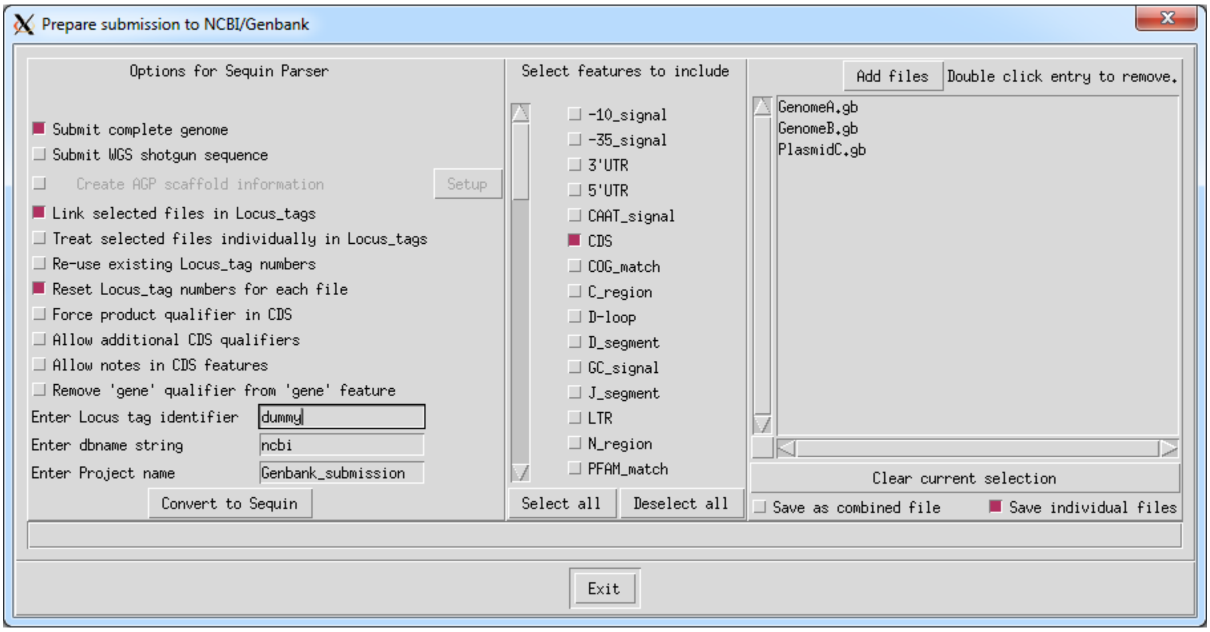

(xv) *'protein_id' unique identifier*: NCBI requires a unique tracker for all proteins which is realised via the 'protein_id'. The format for 'protein_id is 'gnl|dbname|string', whereby 'string' represents the locus tag. 'dbname' should be a unique version of your lab name. Enter the dbname into the respective textfield next to 'Enter dbname string'.

(xvi) *Project name*: If the 'Save individual files' option is selected, Sequin conversions are saved into separate directories. Enter the project name into the textbox next to 'Enter Project name'.

(xvii) *Included Features for the Sequin annotation table*:

By default, only 'gene' and 'CDS' features are selected to be included in the Sequin annotation table. When annotated, adding 'tRNA' and 'rRNA' features is recommend. A more verbose annotation can be created by adding more features in the 'Select features to include' box. To select all possible features, click on 'Select all', remove all features by clicking on 'Deselect all'.

(xviii) *Start Sequin Conversion*:

To start the Sequin conversion, click on the 'Convert to Sequin' button. A new Dialog Box will appear once the conversion is complete.

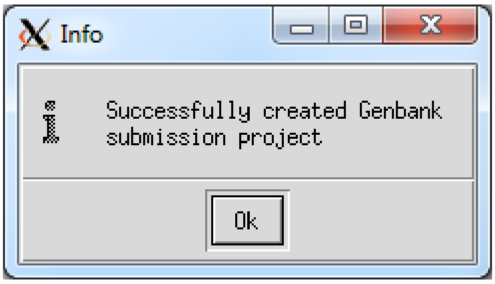

(xix) *AGP scaffold file for draft genomes*:

If draft genomes were prepared and the scaffolding information provided, an AGP file will be part of the Sequin conversion output. The AGP file follows the NCBI guidelines, but assumes certain parameters:

- The size of the gap is unknown 'U' and set to a default length of 100
- The gap type is 'contig'
- No evidence of linkage

To adapt the AGP file to the specifics of a given assembly, refer to the AGP File Format table (http://www.ncbi.nlm.nih.gov/assembly/agp/AGP_Specification/) and search&replace the respective sections in the AGP text file using a word editor.

Example of an AGP output file:

```
##compatible to agp-version 2.0
# ORGANISM: [organism=Escherichia coli] [strain=BL21]
scaffold1     1     1307  1     W     contig0013 1     1307  +
scaffold1     1308  1407  2     U     100   contig     no    na
scaffold1     1408  23774 3     W     contig0023 1     22367 +
scaffold1     23775 23874 4     U     100   contig     no    na
scaffold1     23875 30215 5     W     contig0032 1     6341  +
scaffold1     30216 30315 6     U     100   contig     no    na
```

(xx) *Sequin Submission*:

Download the latest version of Sequin (ftp://ftp.ncbi.nih.gov/sequin/) and install.

Enter Author information and import the FASTA sequence file when prompted ('Import Nucleotide FASTA').

Set the genetic code to "Bacterial, Archaeal and Plant Plastid" for all molecules.

When asked for annotation, select the 'None' option.

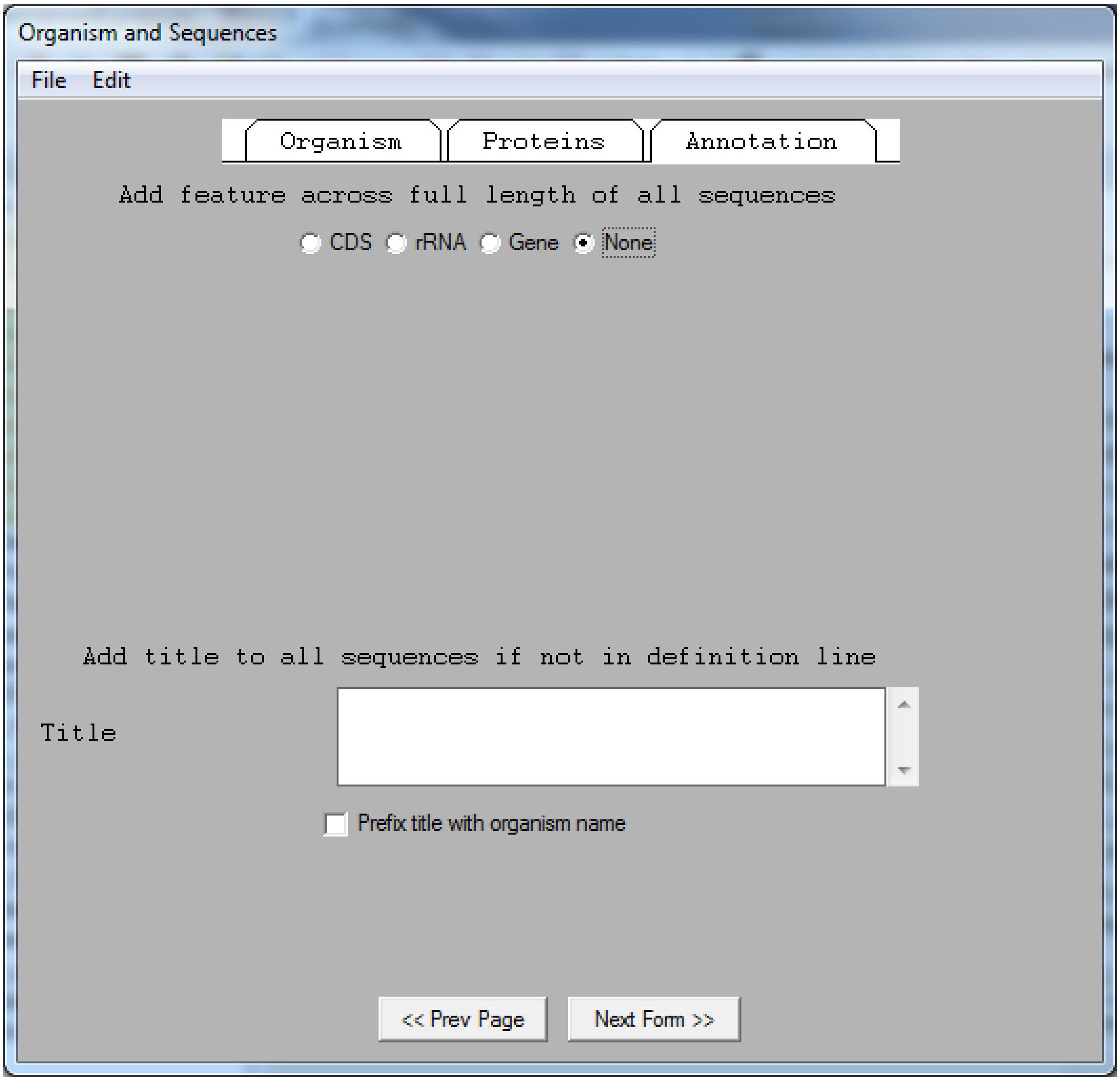

Continue to record viewer

Add annotation: goto 'File -> Open' and chose the .tbl file.

Validate the submission via 'Search -> Validate' and correct all warning and errors

Save the file and then select 'File -> Prepare submission' to create the final file for submission to NCBI.

Good luck!

6.3 *Annotation*

One of the challenges of annotating (draft phase) genomes are changes occurring to the gene model and various ORF annotations due to sequence updates and manual curations. To avoid redoing entire annotations, GAMOLA2 features a module that will transfer an existing annotation onto a new sequence version.

6.3.1 *Transfer annotation between Genbank files*

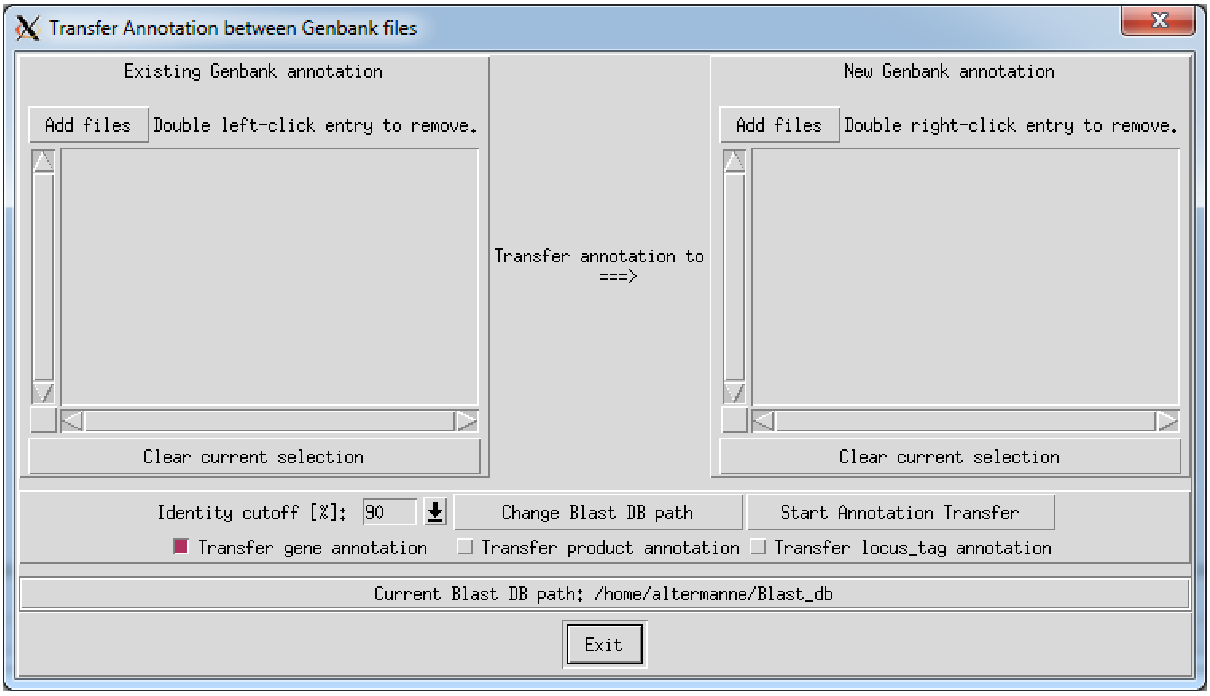

Note: The new version must have been run through GAMOLA2 prior to the annotation transfer. Input for the module are Genbank files. Multiple Genbank files for existing and new annotations are accepted.

Short description of the search algorithm:

For each query ORF GAMOLA2 first tries to find an unchanged subject ORF, based on the respective deduced amino acid sequences. When respective ORF aa sequences have changed, BLASTP is initiated, comparing the old aa sequence against a Blast database comprising all new ORF aa sequences. When a hit is found that exhibits x % aa identity over the entire alignment, the old ORF is assigned to the respective new ORF. When multiple hits are encountered above the selected

threshold (‘*Identity cutoff*’), only the best hit is used. Duplicate subject ORFs will be captured as well. Where no subject hits above the threshold are found, the query ORF is added to the query negative hit list. Once all query ORFs have been analysed and assigned to respective subject ORFs, remaining, unassigned subject ORFs are investigated with the aim to find their likely query counterpart using direct sequence and subsequent BLASTP analyses. Remaining unassigned subject ORFs are collated.

Result files are:

(a) The subject Genbank file comprising the transferred annotation.

(b) A textfile that lists the ORF numbers changes from query to subject Genbank files (‘Old_to_new_ORFnumbers.txt’).

(c) A textfile listing unassigned ORFs in the subject (new) Genbank file (‘Unassigned_ORFs_in_new_Genbank_files.txt’)

(d) A textfile listing unassigned ORFs in the query (old) Genbank file (‘Unassigned_ORFs_from_existing_annotation.txt’)

(e) A textfile listing ambiguous hits, where multiple potential ORFs were found (e.g. multiple copies of an ORF as often seen for transposases, ‘Multiple_hits_in_existing_annotation.txt’).

Annotation transfer between two Genbank files:

(i) *Query Genbank*: Select the query Genbank file(s) that host(s) the existing annotation by clicking on the ‘Add files’ button on the left (‘Existing Genbank annotation”). Double click on an entry to remove from the list. Click on ‘Clear current selection’ to remove all entries.

(ii) *Subject Genbank*: Select the subject Genbank file(s) that will receive the existing annotation by clicking on the ‘Add files’ button on the left (‘New Genbank annotation”). Double click on an entry to remove from the list. Click on ‘Clear current selection’ to remove all entries

(iii) *Identity cutoff*: In cases where the amino acid sequence of query and subject ORFs has changed this defines the minimum sequence identity ([%]) over the BLASTP alignment required to assign respective query and subject ORFs. Default value is 90%. Change this value either by clicking on the down-arrow or by directly typing into the field right to ‘Identity cutoff [%]’.

(iv) *Blast database path*: When invoked, the Annotation transfer window will display the current path where BLAST databases will be saved to. Verify that the displayed path is correct, if not, choose a different path by clicking on the ‘Change Blast DB path’ button.

(v) *Annotation transfer options*: By default, only the ‘gene’ qualifier is transferred between ‘gene’ and ‘CDS’ features (Check tickbox ‘Transfer gene annotation’). Additional choices include ‘product’ (tickbox ‘Transfer product annotation’) and ‘locus_tag’ (tickbox ‘Transfer locus_tag annotation’) qualifiers.

(vi) *Start annotation transfer*: To begin the annotation transfer, click on the ‘Start Annotation Transfer’ button. Depending on the number of ORFs and the degree of changes between query and subject files, runtime may vary considerably.

(vii) Successful run: Once the annotation transfer has finished, a pop up window will appear.

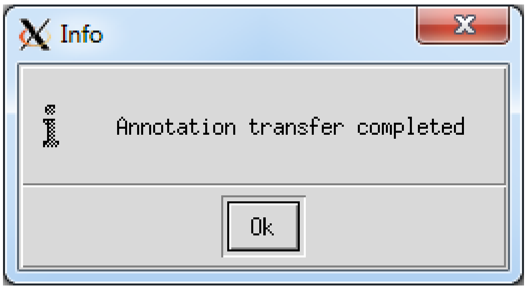

(viii) Leave the application by pressing the ‘Exit’ button.

6.4 Metagenome

6.4.1 Metagenome analysis

Note: This module is experimental and was added by a specific user request. It is a rather specialised analysis, so the use to you and the mileage you get out of this analysis may vary.

The purpose of this module is to give a fast overview on the distribution of specific functional hits within metagenome reads. This is accomplished by first creating custom Blast databases that comprise entries relevant to the biological function(s) of interest. For example, one may first download sequences of ‘glycosylhydrolase’ enzymes from NCBI in FASTA format and use the GAMOLA2 custom database module to build a Blast database. Metagenomic reads can then be searched against this database and hits below a defined e-value threshold will be retrieved and collated.

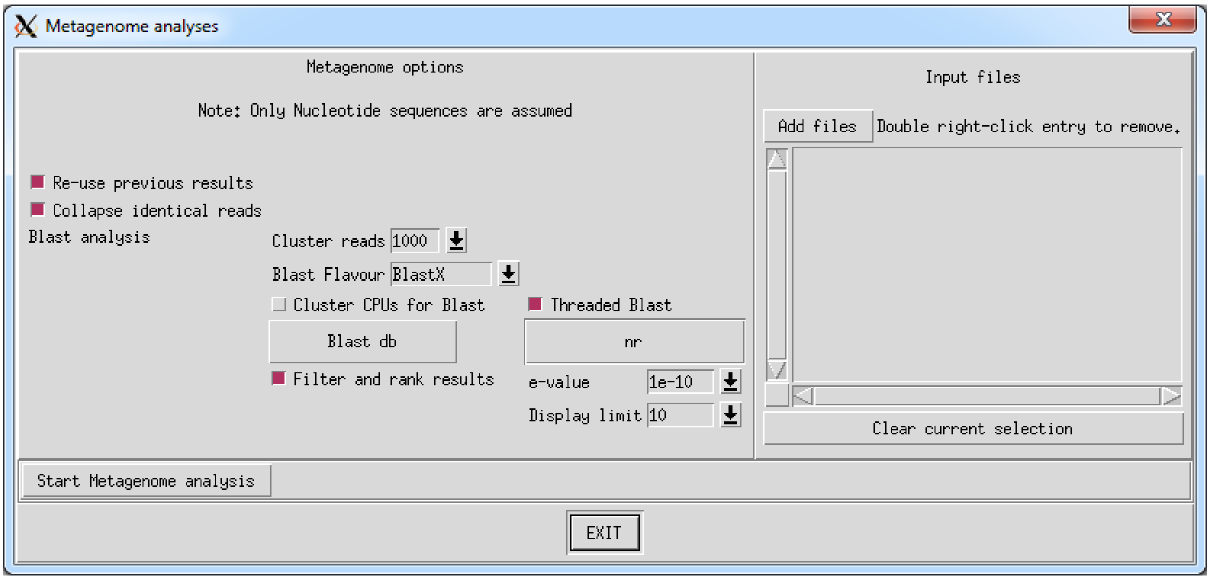

Requirements for a metagenome analysis:

(i) Metagenome reads in FASTA format

(ii) Create a nucleotide or amino-acid BLAST database of relevant sequences. Usually those can be downloaded from NCBI.

Metagenome analysis:

(i) *Input files*: Select the FASTA input file(s) that contain the metagenome sequence reads by clicking on the 'Add files' button on the right. Remove individual files from the selection via double-click. Clear the entire selection by clicking on the 'Clear current selection' button.

(ii) *Re-use results*: If a previous run was interrupted or additional metagenome reads were added to the analysis, already existing Blast results can be used if the same database is selected. This will reduce required runtimes. To enable the re-use of existing Blast results, tick the 'Re-use previous results' tickbox.

(iii) *Collapse Reads*: Metagenome reads may contain varying numbers of identical sequence reads. By identifying such identical sequences and removing duplicates from the Blast queue, runtime for the analysis can be reduced. FASTA headers of duplicate reads are combined and individual reads and their respective numbers can therefore be easily retrieved. To eliminate such read duplicates, tick the 'Collapse identical reads' tickbox.

(iv) *Blast analysis*: The number of used CPUs or threads is the same as used for the main GAMOLA2 software and can be changed in the 'System properties' tab (see above).

(v) *Cluster Reads*: Blast runtimes can be drastically reduced by submitting multiple sequences to the same instances (batch Blast). Select an appropriate number of sequences to be combined in a single instance in the filed right to 'Cluster reads'.

(vi) *Blast Flavour*: At this point, the module supports three different types of Blast: BlastN (nt database required, search nucleotide databases using a nucleotide query), BlastX (aa database required, search protein databases using a translated nucleotide query) and tBlastX (nt database required, search translated nucleotide databases using a translated

nucleotide query). Either BlastX or tBlastX are the recommended Blast flavours. Select the preferred option in the field right to 'Blast Flavour'.

(vii) *Parallel executions*: When multiple processors or cores are available, BLAST can be accelerated by either assigning all CPUs/cores to one Blast instance (select the 'Cluster CPUs for Blast' tickbox) or by running multiple Blast instances in parallel (select the 'Threaded Blast' tickbox).

(viii) *Blast database*: While generic Blast databases can be used, specialised, custom Blast databases will deliver the greatest benefit for this type of analysis. To change the current Blast database, click on the 'Blast db' button and select the appropriate Blast database.

(ix) *Filter and rank results*: In addition to the raw Blast output, results can be filtered by an upper e-value threshold and subsequently hits to databases ranked by respective frequencies. These rankings will be given for both 'collapsed' and full read datasets. The maximum number of displayed hits below the chosen e-value threshold can be set to reduce the size of the output files.

To filter and rank results, tick the corresponding checkbox.

e-value thresholds can be changed in the field right to 'e-value'

The maximum number of Blast hits per read can be limited through the 'Display limit' option.

(x) *Start of Analysis*: The metagenome analysis can be started by pressing the 'Start Metagenome analysis' button.

Results of the metagenome analysis:

(i) Raw Blast output: Blast outputs are saved as a single file ('metagenome.blast_flavour') into the 'Blast results' folder. Each hit comprises a header (listing the used Blast Flavour, the Blast database name, the Query ID) and the Blast overview, limited by the display limit chosen.
Example:

```
Blast Flavour: BlastX
Blast db:      /Blast_db/metagenome.blastx.db
Query name:    FA8DASD02AAD
Blast overview

>gb|gi_8992| 14029606|gb|AAK52749.1|AF354648_1 amylase-binding protein B [Streptococcus gordonii]~~~~~~~~~~~~ Length=652 Score=79 Expect=7e-19
>gb|gi_12296|21911299|ref|NP_665567.1| putative dipeptidase [Streptococcus pyogenes MGAS315]~~~~~~~~~~~~~~~~~ Length=499 Score=73 Expect=1e-16
>gb|gi_3956| 21905513|gb|AAM80370.1| putative dipeptidase [Streptococcus pyogenes MGAS315]~~~~~~~~~~~~~~~~~~~ Length=499 Score=73 Expect=1e-16
>gb|gi_18700|150836415|gb|ABR70391.1| glycogen debranching enzyme GlgX [Marinomonas sp. MWYL1]~~~~~~~~~~~~~~~ Length=679 Score=32 Expect=0.047
>gb|gi_13429|152995491|ref|YP_001340326.1| glycogen debranching enzyme GlgX [Marinomonas sp. MWYL1]~~~~~~~~~~ Length=679 Score=32 Expect=0.047
>gb|gi_29061|198272285|gb|EDY96554.1| hypothetical protein BACPLE_00997 [Bacteroides plebeius DSM 17135]~~~~~ Length=974 Score=25 Expect=9.9
>gb|gi_10881|198274838|ref|ZP_03207370.1| hypothetical protein BACPLE_00997 [Bacteroides plebeius DSM 17135]~ Length=974 Score=25 Expect=9.9


Blast Flavour: BlastX
Blast db:      /Blast_db/metagenome.blastx.db
Query name:    FASJXSCF1B9FR1
Blast overview
>No Blast Hits found


Blast Flavour: BlastX
Blast db:      /Blast_db/metagenome.blastx.db
Query name:    FI8JRFF0GJZ17S
Blast overview

>gb|gi_28651|89951737|gb|ABD81752.1| glycoside hydrolase family 2, sugar binding [Saccharophagus degradans 2-40]~ Length=661 Score=26 Expect=2.6
>gb|gi_14471|90022137|ref|YP_527964.1| ExsB [Saccharophagus degradans 2-40]~~~~~~~~~~~~~~~~~~~~~~~~~~~~~~~~~~~~~~ Length=661 Score=26 Expect=2.6
>gb|gi_19343|162454284|ref|YP_001616651.1| putative mannanase [Sorangium cellulosum 'So ce 56']~~~~~~~~~~~~~~~~~~ Length=554 Score=25 Expect=6.5
>gb|gi_9759| 161164866|emb|CAN96171.1| Putative mannanase [Sorangium cellulosum 'So ce 56']~~~~~~~~~~~~~~~~~~~~~~ Length=554 Score=25 Expect=6.5
>gb|gi_29364|163776808|gb|EDQ90426.1| predicted protein [Monosiga brevicollis MX1]~~~~~~~~~~~~~~~~~~~~~~~~~~~~~~~ Length=686 Score=25 Expect=6.6
>gb|gi_864|  167520276|ref|XP_001744477.1| predicted protein [Monosiga brevicollis MX1]~~~~~~~~~~~~~~~~~~~~~~~~~~ Length=686 Score=25 Expect=6.6


Blast Flavour: BlastX
Blast db:      /Blast_db/metagenome.blastx.db
Query name:    ZC8JXSSS1CWWFJ
Blast overview

>gb|gi_29927|166714148|ref|ZP_02245355.1| xylosidase; arabinosidase [Xanthomonas oryzae pv. oryzicola BLS256]~ Length=344 Score=25 Expect=9.3
>gb|gi_26886|84625833|ref|YP_453205.1| xylosidase [Xanthomonas oryzae pv. oryzae MAFF 311018]~~~~~~~~~~~~~~~~~ Length=344 Score=25 Expect=9.3
>gb|gi_22128|21110696|gb|AAM39093.1| xylosidase; arabinosidase [Xanthomonas axonopodis pv. citri str. 306]~~~~ Length=344 Score=25 Expect=9.3
>gb|gi_14181|84369773|dbj|BAE70931.1| xylosidase [Xanthomonas oryzae pv. oryzae MAFF 311018]~~~~~~~~~~~~~~~~~~ Length=344 Score=25 Expect=9.3
>gb|gi_13462|21244975|ref|NP_644557.1| arabinosidase [Xanthomonas axonopodis pv. citri str. 306]~~~~~~~~~~~~~~ Length=344 Score=25 Expect=9.3
>gb|gi_9076| 58584056|ref|YP_203072.1| xylosidase; arabinosidase [Xanthomonas oryzae pv. oryzae KACC10331]~~~~ Length=344 Score=25 Expect=9.3
>gb|gi_8541| 58428650|gb|AAW77687.1| xylosidase; arabinosidase [Xanthomonas oryzae pv. oryzae KACC10331]~~~~~~ Length=344 Score=25 Expect=9.3
```

(ii) *Sorted results*: A second output file ‘sorted_results’, lists all (collapsed) reads below the selected e-value threshold. The tabulated list is sorted by ascending e-values.

Each entry comprises 6 data fields (columns):
Entry number,
Access key (usually the read ID),
Sequence frequencies (for collapsed reads this can be > 1),
Best Blast e-value,
the used Blast database and
a Blast overview, limited to ‘n’ entries as defined by the chosen display limit.

The list can be directly imported into Excel for further analyses and data mining.

Example output:

<table>
<tr><th>Entry number</th><th>Access key</th><th>Sequence frequency</th><th>Best E-value</th><th>Used Database</th><th>Sequence</th><th>Blast overview</th></tr>
<tr><td>1</td><td>FI8JXSF01B6LI5</td><td>1</td><td>6e-33</td><td>metagenome.blastx.db</td><td>CTAACGTCTATGTGCTCGTGGCCCAGTTAG…</td><td>>gb|gi_25115|60494108|emb|CAH08900.1| putative exported beta-galactosidase [Bacteroides fragilis NCTC 9343]~~ Length=1341 Score=119 Expect=6e-33</td></tr>
<tr><td></td><td></td><td></td><td></td><td></td><td></td><td>>gb|gi_22823|53714661|ref|YP_100653.1| beta-galactosidase [Bacteroides fragilis YCH46]~~ Length=1341 Score=119 Expect=6e-33</td></tr>
<tr><td></td><td></td><td></td><td></td><td></td><td></td><td>>gb|gi_10286|52217526|dbj|BAD50119.1| beta-galactosidase [Bacteroides fragilis YCH46]~~ Length=1341 Score=119 Expect=6e-33</td></tr>
<tr><td></td><td></td><td></td><td></td><td></td><td></td><td>>gb|gi_5074| 60682674|ref|YP_212818.1| putative exported beta-galactosidase [Bacteroides fragilis NCTC 9343]~~ Length=1341 Score=119 Expect=6e-33</td></tr>
<tr><td></td><td></td><td></td><td></td><td></td><td></td><td>>gb|gi_27471|153807433|ref|ZP_01960101.1| hypothetical protein BACCAC_01713 [Bacteroides caccae ATCC 43185]~~ Length=1357 Score=117 Expect=2e-32</td></tr>
<tr><td></td><td></td><td></td><td></td><td></td><td></td><td>>gb|gi_2911| 149129795|gb|EDM21007.1| hypothetical protein BACCAC_01713 [Bacteroides caccae ATCC 43185]~~ Length=1357 Score=117 Expect=2e-32</td></tr>
<tr><td></td><td></td><td></td><td></td><td></td><td></td><td>>gb|gi_25071|29349458|ref|NP_812961.1| beta-galactosidase [Bacteroides thetaiotaomicron VPI-5482]~~ Length=1342 Score=116 Expect=6e-32</td></tr>
<tr><td></td><td></td><td></td><td></td><td></td><td></td><td>>gb|gi_17439|29341367|gb|AAO79155.1| beta-galactosidase [Bacteroides thetaiotaomicron VPI-5482]~~ Length=1342 Score=116 Expect=6e-32</td></tr>
<tr><td></td><td></td><td></td><td></td><td></td><td></td><td>>gb|gi_17266|154088759|gb|EDN87803.1| hypothetical protein PARMER_00928 [Parabacteroides merdae ATCC 43184]~~ Length=1343 Score=112 Expect=2e-30</td></tr>
<tr><td></td><td></td><td></td><td></td><td></td><td></td><td>>gb|gi_12002|154491011|ref|ZP_02030952.1| hypothetical protein PARMER_00928 [Parabacteroides merdae ATCC 43184]~~ Length=1343 Score=112 Expect=2e-30</td></tr>
<tr><td></td><td></td><td></td><td></td><td></td><td></td><td></td></tr>
<tr><td>2</td><td>FI8JXSF01A06FP</td><td>1</td><td>1e-32</td><td>metagenome.blastx.db</td><td>TTGTGGTGATAAGGTCTCCAGAGCGGATCGA…</td><td>>gb|gi_27576|160931681|ref|ZP_02079075.1| hypothetical protein CLOLEP_00512 [Clostridium leptum DSM 753]~~ Length=675 Score=118 Expect=1e-32</td></tr>
<tr><td></td><td></td><td></td><td></td><td></td><td></td><td>>gb|gi_15265|156869326|gb|EDO62698.1| hypothetical protein CLOLEP_00512 [Clostridium leptum DSM 753]~~ Length=675 Score=118 Expect=1e-32</td></tr>
<tr><td></td><td></td><td></td><td></td><td></td><td></td><td>>gb|gi_22953|76882735|gb|ABA57416.1| 1,4-alpha-glucan branching enzyme [Nitrosococcus oceani ATCC 19707]~~ Length=749 Score=118 Expect=1e-32</td></tr>
<tr><td></td><td></td><td></td><td></td><td></td><td></td><td>>gb|gi_17674|118572375|sp|Q3JCN0.1|GLGB_NITOC 1,4-alpha-glucan-branching enzyme (Glycogen-branching enzyme) (BE) (1,4-alpha-D-glucan:1,4-alpha-D-glucan 6-glucosyl-transferase)~~ Length=749 Score=118 Expect=1e-32</td></tr>
<tr><td></td><td></td><td></td><td></td><td></td><td></td><td>>gb|gi_3667| 77164421|ref|YP_342946.1| glycogen branching enzyme [Nitrosococcus oceani ATCC 19707]~~ Length=749 Score=118 Expect=1e-32</td></tr>
<tr><td></td><td></td><td></td><td></td><td></td><td></td><td>>gb|gi_26400|167656498|gb|EDS00628.1| hypothetical protein EUBSIR_01548 [Eubacterium siraeum DSM 15702]~~ Length=702 Score=117 Expect=2e-32</td></tr>
<tr><td></td><td></td><td></td><td></td><td></td><td></td><td>>gb|gi_19238|167750572|ref|ZP_02422699.1| hypothetical protein EUBSIR_01548 [Eubacterium siraeum DSM 15702]~~ Length=702 Score=117 Expect=2e-32</td></tr>
<tr><td></td><td></td><td></td><td></td><td></td><td></td><td>>gb|gi_24996|167769704|ref|ZP_02441757.1| hypothetical protein ANACOL_01038 [Anaerotruncus colihominis DSM 17241]~~ Length=672 Score=116 Expect=6e-32</td></tr>
<tr><td></td><td></td><td></td><td></td><td></td><td></td><td>>gb|gi_7159| 167668065|gb|EDS12195.1| hypothetical protein ANACOL_01038 [Anaerotruncus colihominis DSM 17241]~~ Length=672 Score=116 Expect=6e-32</td></tr>
<tr><td></td><td></td><td></td><td></td><td></td><td></td><td>>gb|gi_24328|118572372|sp|Q1D654.1|GLGB_MYXXD 1,4-alpha-glucan-branching enzyme (Glycogen-branching enzyme) (BE) (1,4-alpha-D-glucan:1,4-alpha-D-glucan 6-glucosyl-transferase)~~ Length=734 Score=115 Expect=8e-32</td></tr>
<tr><td></td><td></td><td></td><td></td><td></td><td></td><td></td></tr>
<tr><td>3</td><td>FI8JXSF01BQUL2</td><td>1</td><td>8e-31</td><td>metagenome.blastx.db</td><td>GCCCCGCCCGCGGCGTACCAGGACGGCATGA…</td><td>>gb|gi_22725|156869166|gb|EDO62538.1| hypothetical protein CLOLEP_00660 [Clostridium leptum DSM 753]~~ Length=669 Score=112 Expect=8e-31</td></tr>
<tr><td></td><td></td><td></td><td></td><td></td><td></td><td>>gb|gi_3452| 160931829|ref|ZP_02079222.1| hypothetical protein CLOLEP_00660 [Clostridium leptum DSM 753]~~ Length=669 Score=112 Expect=8e-31</td></tr>
<tr><td></td><td></td><td></td><td></td><td></td><td></td><td>>gb|gi_15176|60682369|ref|YP_212513.1| hypothetical protein BF2899 [Bacteroides fragilis NCTC 9343]~~ Length=1200 Score=62 Expect=9e-13</td></tr>
<tr><td></td><td></td><td></td><td></td><td></td><td></td><td>>gb|gi_7342| 60493803|emb|CAH08593.1| conserved hypothetical protein [Bacteroides fragilis NCTC 9343]~~ Length=1200 Score=62 Expect=9e-13</td></tr>
<tr><td></td><td></td><td></td><td></td><td></td><td></td><td>>gb|gi_4566| 53714351|ref|YP_100343.1| hypothetical protein BF3063 [Bacteroides fragilis YCH46]~~ Length=1253 Score=62 Expect=9e-13</td></tr>
<tr><td></td><td></td><td></td><td></td><td></td><td></td><td>>gb|gi_2466| 52217216|dbj|BAD49809.1| hypothetical protein [Bacteroides fragilis YCH46]~~ Length=1253 Score=62 Expect=9e-13</td></tr>
<tr><td></td><td></td><td></td><td></td><td></td><td></td><td>>gb|gi_27641|153807504|ref|ZP_01960172.1| hypothetical protein BACCAC_01784 [Bacteroides caccae ATCC 43185]~~ Length=601 Score=61 Expect=2e-12</td></tr>
<tr><td></td><td></td><td></td><td></td><td></td><td></td><td>>gb|gi_10826|149129866|gb|EDM21078.1| hypothetical protein BACCAC_01784 [Bacteroides caccae ATCC 43185]~~ Length=601 Score=61 Expect=2e-12</td></tr>
<tr><td></td><td></td><td></td><td></td><td></td><td></td><td>>gb|gi_25045|154486721|ref|ZP_02028128.1| hypothetical protein BIFADO_00545 [Bifidobacterium adolescentis L2-32]~~ Length=680 Score=55 Expect=1e-10</td></tr>
<tr><td></td><td></td><td></td><td></td><td></td><td></td><td>>gb|gi_891| 154084584|gb|EDN83629.1| hypothetical protein BIFADO_00545 [Bifidobacterium adolescentis L2-32]~~ Length=680 Score=55 Expect=1e-10</td></tr>
<tr><td></td><td></td><td></td><td></td><td></td><td></td><td></td></tr>
</table>

(iii) *General binned overview files*: Finally, two tab-delimited overview files are provided, displaying the number of (collapsed) hits within a certain e-value range. These files are useful for generating a graphical overview of hit frequencies and respective levels of similarities within a given metagenome against a functional database (see below).

The first file ‘binning.txt’ provides a high-level overview, with e-value thresholds of 1, 1e-10, 1e-40 and 1e-100.
Example output:

e-value range	Distribution	Uncollapsed frequency
1	62256	70889
1e-10	146	174
1e-40	0	0
1e-100	0	0

```
Total number of sequence reads71078
```

‘Distribution’: number of collapsed hits, ‘Uncollapsed frequency’: actual number of read hits

The second file ‘detailed_binning.txt’, expands the e-value thresholds and provides a more finely granulated stepping. E-value thresholds used are 1, 1e-10, 1e-20, 1e-30, 1e-40, 1e-50, 1e-60, 1e-70, 1e-80, 1e-90, 1e-100, 1e-110, 1e-120, 1e-130, 1e-140, 1e-150, and 1e-160.
Example output:

e-value range	Distribution	Uncollapsed frequency
1	62256	70889
1e-10	133	174
1e-20	10	12
1e-30	3	3
1e-40	0	0
1e-50	0	0
1e-60	0	0
1e-70	0	0
1e-80	0	0
1e-90	0	0
1e-100	0	0
1e-110	0	0
1e-120	0	0
1e-130	0	0
1e-140	0	0
1e-150	0	0
1e-160	0	0

```
Total number of sequence reads      71078
```

(iv) *Graphical representation*: A possible application is the comparison across multiple metagenomes over multiple specialised databases.

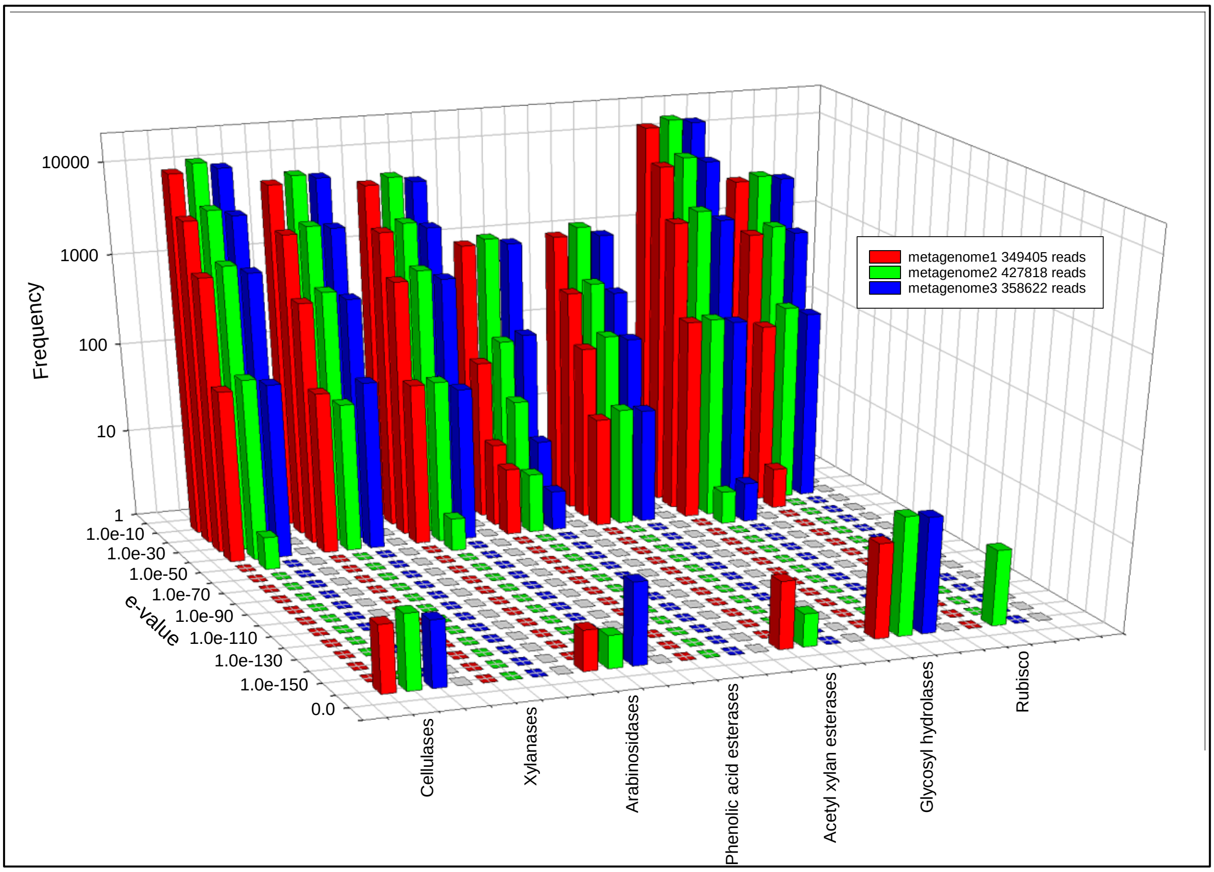

This graph was created with Sigma Plot and visualises the hit frequency distribution across 7 custom Blast databases while comparing three different metagenomes. Hit frequencies and levels of homology (via e-value) can be directly compared. (X-axis: custom Blast databases, Y-axis: e-value threshold levels, Z-axis: hit frequencies at a given e-value threshold).

7 *Annotation Guidelines (suggestion only)*

Every group working with and analysing/comparing (draft)genomes has their own time-proven system to deal with manal curation of computer based annotations, version tracking and data handling. Below is a short list of recommended standards we have developed in our own team that works well for us (applied microbiology). Feel free to adopt these guidelines – or adapt/modify/ignore to make things work best for your team. The annotation guidelines are written to coordinate a team of multiple annotators, but work equally well for individuals taking on the task.

(1) Use your own copy of the Genbank file on your local computer. Master files on the server (or wherever you store them) are not to be modified. Once the GOLD standard has been established changes to the annotation will be implemented by the curator and the updated file will be copied onto the server for everybody. This ensures consistency throughout the process. (You can do whatever you like to your local copy ☺)

(2) Keys/features comprise: CDS, gene, TIGRfam,…
Qualifiers within features comprise: notes, gene, product,…

(3) NEVER modify/delete the "_ORFnumber" ending of gene qualifiers

(4) Select "show products" in the Artemis::feature list

(5) Keep the original Genbank file / folder structure – always copy the entire set if you want to retain the ability to look up underlying results.

(6) Retrieving underlying results: Select a 'gene' or 'CDS' feature, right-click and select 'View -> View Customised Annotation Results -> …' to investigate Blast, COG, PFam or TIGRfam hits for a respective ORF.

(7) Modify features in Artemis by using the <CTRL+E> shortcut

(8) Save Genbank file by pressing <CTRL+S> shortcut – never otherwise

(9) CDS::gene has a verbose description of function, including gene symbol if possible (i.e. DNA polymerase, DnaA (→ capitalized DnaA!))

(10) Gene::gene is the shortest possible symbol (i.e. dnaA (→ lower case, gene symbol) or cons. hypo)

(11) Use a common descriptor for functional groups in gene::gene (i.e ABCtransporter) and the verbose descriptor in CDS::gene (i.e. cobalt ABC transporter, permease subunit)

(12)Symbols for annotation:

“[…..]_1” if functional annotation is unsure

”>>….<< to 2_1” for frameshifts. Smaller/less significant part refers to main ORF.

”>> <<_1” for truncated ORFs (premature stop codon, potential, not verified frameshift)

”cons. hypo” and “unknown” ORFs automatically get “[…]” tags

combinations of tags are possible

(13) - Use “cons. hypo” for ORFs with non-classified homologs

- Use “unknown” for ORFs without any homologs or all homologs >1e-10 (maybe select a lower/higher threshold for specific purposes?)

(14)Preferrably use TIGRfam to find gene symbols: try to find the most common name

(15)Gene and CDS keys always have the same start/stop positions

(16)Start/stop points of any feature can be changed by drag-n-slide in Artemis. Take care to not shift frames!

(17)If modifying start position of gene/CDS, make sure to manually modify aa sequence in CDS::translation. Artemis does not automatically update ‘translation’ qualifiers.

(18)Verify start position of ORFs by checking CDS::product (best Blast hit): score and length in aa and subsequently aa alignment in Blast-result file (using new modArtemis8)

(19)Verify RBS position – a widely used consensus is “AGGAGGAG”, you may want to use a more specific one for your sequences.

(20)If features need to be added, only “free” regions can be used as start point. If necessary, select region on opposite strand and select “complement” in the feature window.

(21)Verify error.log for your sequences when transferring annotations -> any ORFs with multiple or ambiguous assignments?

(22)Track your gene model changes in an Excel file (old gene model -> new gene model); this will later also incorporate changes in nt sequence, functional gene re-classification, adding/removing of features, etc (after the first GOLD standard has been established)

(23)Using the weekly annotation meeting to discuss progress and help with difficult ORFs

Reference list

1. **Altermann, E., and T. R. Klaenhammer.** 2003. GAMOLA: a new local solution for sequence annotation and analyzing draft and finished prokaryotic genomes. Omics **7:**161-169.
2. **Altschul, S. F., T. L. Madden, A. A. Schaffer, J. Zhang, Z. Zhang, W. Miller, and D. J. Lipman.** 1997. Gapped BLAST and PSI-BLAST: a new generation of protein database search programs. Nucleic acids research **25:**3389-3402.
3. **Badger, J. H., and G. J. Olsen.** 1999. CRITICA: coding region identification tool invoking comparative analysis. Molecular biology and evolution **16:**512-524.
4. **Bland, C., T. L. Ramsey, F. Sabree, M. Lowe, K. Brown, N. C. Kyrpides, and P. Hugenholtz.** 2007. CRISPR recognition tool (CRT): a tool for automatic detection of clustered regularly interspaced palindromic repeats. BMC Bioinformatics **8:**209.
5. **Camacho, C., G. Coulouris, V. Avagyan, N. Ma, J. Papadopoulos, K. Bealer, and T. L. Madden.** 2009. BLAST+: architecture and applications. BMC Bioinformatics **10:**421.
6. **Delcher, A. L., K. A. Bratke, E. C. Powers, and S. L. Salzberg.** 2007. Identifying bacterial genes and endosymbiont DNA with Glimmer. Bioinformatics.
7. **Dyrlov Bendtsen, J., H. Nielsen, G. von Heijne, and S. Brunak.** 2004. Improved Prediction of Signal Peptides: SignalP 3.0. Journal of Molecular Biology **340:**783-795.
8. **Eddy, S. R.** 2009. A new generation of homology search tools based on probabilistic inference. Genome Inform **23:**205-211.
9. **Griffiths-Jones, S., A. Bateman, M. Marshall, A. Khanna, and S. R. Eddy.** 2003. Rfam: an RNA family database. Nucl. Acids Res. **31:**439-441.
10. **Hyatt, D., G. L. Chen, P. F. Locascio, M. L. Land, F. W. Larimer, and L. J. Hauser.** 2010. Prodigal: prokaryotic gene recognition and translation initiation site identification. BMC Bioinformatics **11:**119.
11. **Kall, L., A. Krogh, and E. L. Sonnhammer.** 2004. A combined transmembrane topology and signal peptide prediction method. J Mol Biol **338:**1027-1036.
12. **Kingsford, C. L., K. Ayanbule, and S. L. Salzberg.** 2007. Rapid, accurate, computational discovery of Rho-independent transcription terminators illuminates their relationship to DNA uptake. Genome Biol **8:**R22.
13. **Lowe, T. M., and S. R. Eddy.** 1997. tRNAscan-SE: a program for improved detection of transfer RNA genes in genomic sequence. Nucleic acids research **25:**955-964.
14. **Rutherford, K., J. Parkhill, J. Crook, T. Horsnell, P. Rice, M. A. Rajandream, and B. Barrell.** 2000. Artemis: sequence visualization and annotation. Bioinformatics **16:**944-945.
15. **Suzek, B. E., M. D. Ermolaeva, M. Schreiber, and S. L. Salzberg.** 2001. A probabilistic method for identifying start codons in bacterial genomes. Bioinformatics **17:**1123-1130.
